# Cannibalism, Kuru, and Mad Cows: Prion Disease As a “Choose-Your-Own-Experiment” Case Study to Simulate Scientific Inquiry in Large Lectures

**DOI:** 10.1371/journal.pbio.1002351

**Published:** 2016-01-20

**Authors:** Antonio Serrano, Jeffrey Liebner, Justin K. Hines

**Affiliations:** 1 Department of Chemistry, Lafayette College, Easton, Pennsylvania, United States of America; 2 Department of Mathematics, Lafayette College, Easton, Pennsylvania, United States of America

## Abstract

Despite significant efforts to reform undergraduate science education, students often perform worse on assessments of perceptions of science after introductory courses, demonstrating a need for new educational interventions to reverse this trend. To address this need, we created *An Inexplicable Disease*, an engaging, active-learning case study that is unusual because it aims to simulate scientific inquiry by allowing students to iteratively investigate the Kuru epidemic of 1957 in a choose-your-own-experiment format in large lectures. The case emphasizes the importance of specialization and communication in science and is broadly applicable to courses of any size and sub-discipline of the life sciences.

## Abstract 2

This piece from our Education series uses prion disease as the basis for an active-learning case study that simulates scientific inquiry with a choose-your-own-experiment design. Applicable to courses of any size and subdiscipline of biology.

## Introduction and Scope

In recent years, there has been substantial effort to reform undergraduate science education because of high rates of attrition of science majors often attributed to courses and assessments that students perceive as overly focused on memorization and regurgitation of facts [1–5]. In response to these trends, the National Academies of Sciences, Engineering, and Medicine advocated that science education should integrate scientific principles with real-world problems in order to create students that can critically think and problem-solve [3]. Likewise, the 2011 *Vision and Change* report called for student-centered classrooms that are “interactive, inquiry driven, cooperative, collaborative, and relevant” and curriculum that cultivates informed citizens by teaching core skills required by all scientists, including data interpretation and integration, the application of the scientific method, and the ability to communicate across scientific disciplines [4]. Despite these ambitious goals, students in physics, chemistry, and biology surprisingly perform worse on assessments of student perceptions of these subjects at the end of an introductory course, underscoring the need for new educational interventions to reverse this trend [6–8]. To directly address this problem, we created *An Inexplicable Disease*, a 75-minute, in-class case study designed to be (1) broadly applicable to multiple sub-disciplines of the life sciences, (2) implementable in large lecture courses with minimal course time required, and (3) to simulate scientific inquiry in a way that positively alters students’ perceptions and understanding of biology. In addition to these design goals, we also aimed to create an activity that addressed four of the six core competencies from the *Vision and Change* report, these being: the societal context of science, the collaborative nature of science, the interdisciplinary nature of science, and the evidence-based process of science [4].

The purpose of *An Inexplicable Disease* is to engage students in the process of scientific inquiry through simulated investigation and directed collaboration (see Box 1: Concepts at a Glance). Students work in groups to unknowingly assume the roles of the initial investigators of the Kuru epidemic of the Fore tribe of Papua New Guinea in 1957 by conducting simulated investigations. The activity culminates in a mock-scientific conference, in which groups collaborate by sharing findings, mirroring the real events that led to the discovery of prion diseases (see Box 2: Activity Timeline). Students explore the importance of collaboration and communication between scientists, the difficulty and uncertainty of scientific investigations, and the often unsavory but important utility of animal research. The inquiry-driven activities provide students with opportunities to generate their own insights into how scientific knowledge is generated, tested, disseminated, and used. In addition, students are introduced to relevant biological concepts such as the hierarchy of epidemic disease causes and the role of protein misfolding in amyloid and prion diseases.

**Box 1: Concepts at a Glance**

The activity is broadly applicable across many disciplines of the life sciences in introductory or upper-division courses. Students will practice important skills including critically evaluating data and working in groups.

The case study is designed to engage students in learning:

- How scientific investigations are conducted, including the impact of finite amounts of time and money.
- The importance of specialization in science and the importance of communication and collaboration between scientists of differing specialties.
- Examples in which ethical considerations have factored into important decisions regarding “what can be known.”

**Target age group:** To enable the broadest possible use, the activity was intentionally designed to be useable with first-year students in both majors and non-majors courses. Instructors in advanced courses can add additional course content to “scale up” the scientific content as appropriate for their students and course; e.g., in advanced biochemistry courses, additional emphasis and focus on protein structure and folding can be easily incorporated into the final mini-lecture presentation.

**Box 2: Activity Timeline**

**75 minutes total—**can be continuous or broken into two periods:

**Period 1 (50 minutes):**

 10′ Introduction and disease-causes mini-lecture

 15′ Inquiry-driven activity: Student investigations

 10′ Inquiry-driven activity: Mock-conference

 5′ Class discussion of evidence

 10′ Revelations mini-lecture

**Period 2 (25 minutes):**

 25′ Prions mini-lecture and final class discussion

Part of the novelty of the design of the *Inexplicable Disease* case is the use of an in-class iterative cycle whereby students must choose among a limited set of possible experimental options, followed by the immediate acquisition of new data and the subsequent group discussion of said data, before repeating the cycle with another decision. This approach, which in the context of this in-class activity we call “choose-your-own-experiment” for its similarity to the popular books of similar moniker, differs from most case studies that typically present problems in narrative form followed by questions, brief activities, and opportunities for discussion. Cases involving iterative decision-making and evidence-gathering are common in medical education (and in computer games), but far less common in undergraduate science education and even less so at the introductory level. More importantly, cases of this type are typically implemented either online or offline using a computer or in a laboratory setting or clinical simulation. What is most unusual about our case is that this game-like experience of the case is implementable in the context of a regular lecture course by use of a limited set of authentic, but pre-determined, experiment choices and results. An additional layer of complexity is added by limiting the ability of student groups to gather all the necessary data to complete the case, creating an opportunity for genuine collaboration and discussion with shared interest in a common goal.

The activity has been successfully implemented by numerous early adopters at diverse institutions and in a wide variety of undergraduate courses which, in addition to introductory-level biology, span the fields of cell biology, microbiology, genetics, zoology, and biochemistry. Aggregated data from five courses reveals highly positive responses from students. Additionally, data gathered from two sequential offerings of Introductory Biology indicate that the activity may be successful in producing changes in perceptions of biology in students at the introductory level. We hope that both the activity and the “choose-your-own-experiment” approach to in-class case design will be broadly useful to life sciences educators of many disciplines.

## Engagement: Case Introduction

**Fig 1:**
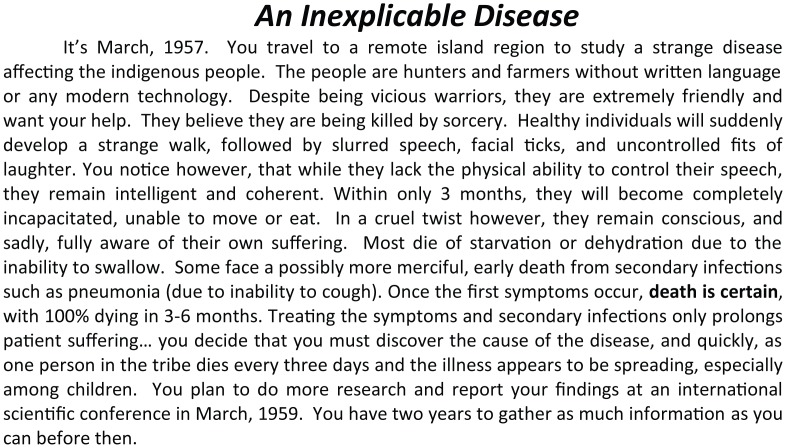
Case introduction to be read aloud in class to begin the activity. Students are alerted in advance that this is a true story as part of the initial case study PowerPoint (S1 PowerPoint).

To facilitate implementation, a detailed description of the case and suggestions for implementation are supplied (see S1 Text). Rules for the activity (S2 Text) are distributed in advance of class with students forming groups of approximately three to five people, depending on course size. Groups receive information packets (S3 and S4 Texts) that allow them to assume the role of a particular type of scientific investigator (physicians or anthropologists). Each group’s specialization then determines the additional information with which they begin the exercise, as well as the specific additional investigation options that are available to them. Groups are charged with answering a basic question regarding the new disease: is it infectious, genetically inherited, or environmentally–behaviorally caused? The instructor gives a brief mini-lecture (S1 PowerPoint) to clarify this question further before beginning the activity by reading the case introduction aloud (Fig 1).

## Inquiry: Student Investigations and Mock Conference

**Fig 2:**
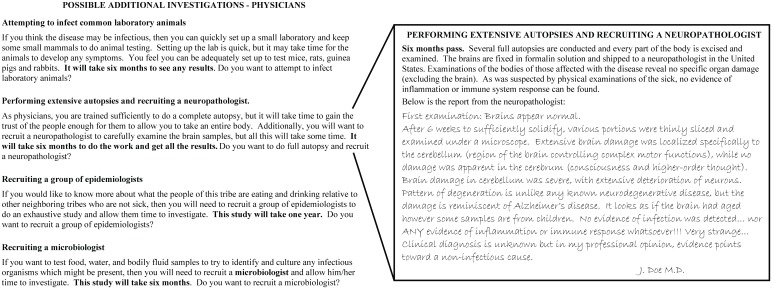
Additional investigations available to “physician” groups. Each group type has a different set of investigation choices available. Group-specific additional case information as well as investigation options are listed in the group packet files (S3 and S4 Texts).

Following the case introduction, student groups discuss and evaluate the available information and choose from a list of additional investigations (Fig 2, left), each deliberately written to emphasize the importance of differing scientific specializations and requiring finite “game time” to conduct. Pre-printed results of investigation choices (S5 Text) are provided one at a time by the instructor as requested by each group (Fig 2, right), allowing groups to iteratively gather new experimental data from the instructor and discuss the results before repeating this cycle of experimentation and discussion until the available game-time is exhausted. The activity gradually reveals conflicting evidence regarding the nature of the disease. Notably, due to some non-overlapping investigation options between investigator types, and insufficient game-time to complete all available investigations, no single group can acquire all the information about that case. Additionally, depending on investigator type and choice of experimentation, groups and individual students typically come to different and conflicting conclusions regarding the nature of the disease, as did the actual investigators; the activity then takes the form of a “jigsaw” in which students share information and ideas with their peers [9]. In a simulation of a real series of meetings in 1959, representatives from each group report their data to other groups, creating opportunities for students to explore why, despite all studying the same disease, they and other groups may have reached conflicting conclusions. Finally, the instructor calls the class together for a final discussion of the evidence and asks for student opinions regarding its interpretation.

## Real Case Revelations: Mini-lectures and Additional Class Discussion

While the ways students acquire knowledge in this activity are clearly simulated, the disease and most of the facts gathered by the students are real. The activity attempts to simulate the difficulties faced by Nobel Laureate Dr. Carleton Gajdusek and coworkers in their initial studies of Kuru (a human prion disease) among the people of the Fore tribe of Papua New Guinea from 1957 to 1959. This information is now finally shared with the students. The instructor explains the factual story behind the activity, including a critical interaction with veterinarian William Hadlow as the result of a scientific meeting in 1959 that led to the eventual resolution of this conundrum by the demonstration that Kuru is an infectious disease via experimentation with chimpanzees. The instructor ends by explaining that, although the nature of the epidemic and the mode of transmission (endocannibalism) have been determined, unbeknownst to the actual investigators at the time, Kuru is in fact a prion disease. The remaining time (or following class period, see Box 2 for activity timeline) consists of a mini-lecture by the instructor (S2 PowerPoint) explaining additional experiments, spanning more than 50 years, which have led to the current state of knowledge regarding prions. This presentation serves as a branch point at which the content coverage can be tailored by instructors to fit various courses. This topic also serves as a timely example of cutting-edge biology, as many questions remain unanswered to this day. Instructors may end the activity with a final class discussion of the topic and an opportunity for student questions.

## Course Context, Scalability, and Other Implementation Issues

The case introduces a large variety of topics that are relevant to various portions of an introductory biology course, and/or are relevant to various upper-level courses in the life sciences. Some of these topics include: protein misfolding, disease agents, genetics, epidemiology, and research ethics. Early adopting instructors in introductory biology courses have often implemented the activity either shortly before or shortly after discussing protein structure, while others have used it in courses to talk about different types of disease agents. Another common use for the activity has been to use it on day one as an introduction to active learning and group work to set the stage and break the ice for students to do additional group and hands-on activities.

Although the activity was explicitly designed for use in lecture-type courses and is fully scalable to large courses, which have included classes of more than 175 students to date, additional students obviously create some logistical issues. The activity is implementable without assistance in sections of at least 60 students, but early adopting instructors with more than this have often done so with the aid of teaching assistants to help answer student questions and facilitate the distribution of experimental results during the activity. However, with proper assistance, the activity is, in principle, infinitely scalable to course size. Another potential issue is reuse from year to year. Instructors should be certain to emphasize that part of the fun of the activity is not knowing the answer. Akin to spoiling a book or a movie, students shouldn’t “spoil” the activity for the next year’s class. With this argument made to students, we have had no issues with reusability in four years of consecutive use in Introductory Biology at our institution and have heard no issues from early adopting instructors regarding this issue.

## Student Feedback

**Fig 3:**
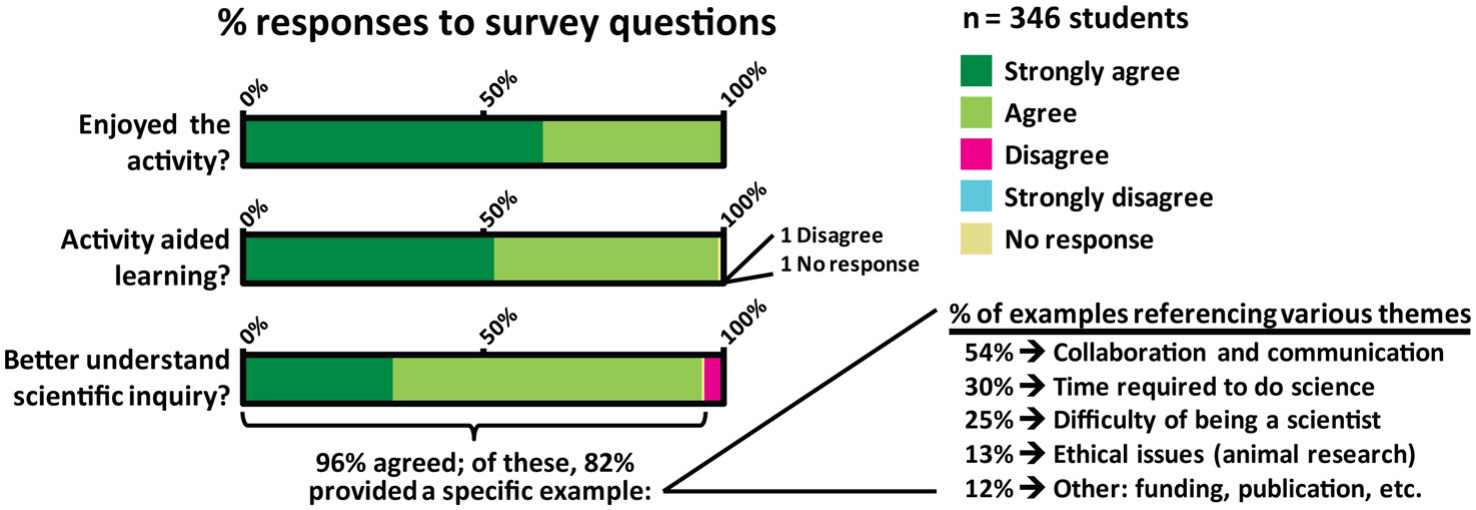
Summary statistics of student reactions and self-reported learning following the activity. Student affective and self-reported learning data were collected and pooled from five courses at three schools. See S6 Text for survey questions and S8 Text for additional details regarding data collection and analysis; a representative listing of various student responses for each theme shown is given in S8 Text.

Student survey data were collected immediately following the administration of the activity and pooled from five courses (see S8 Text for additional details); in total, 346 students responded to the survey (S6 Text). Student responses to the activity were uniformly positive, with 100% reporting that they enjoyed the activity and more than 99% (344 of 346 students) indicating that the activity was useful to their learning (Fig 3). Students were generally emphatic about their enjoyment of the case, which they typically found both exciting and intellectually challenging. Many students commented on the unusual ability of the activity to actually make them feel like a scientist, saying, for example: “…*it would be hard to be real scientists for a day*, *I think this activity was the next best thing*,” and “…*we were on the same track as the scientist*. *We were determined*, *confused*, *but shocked from beginning to end*.”

To begin to clarify what, specifically, students felt that they had learned, we asked whether their understanding of scientific inquiry had improved and, if so, how. More than 96% (333 of 346 students) reported that their understanding of scientific inquiry had changed. Importantly, of the students who agreed, 82% gave at least one specific and relevant example when prompted by the survey. The themes of the most frequently cited examples are shown in Fig 3 and include the importance of communication and collaboration among scientists, the amount of time needed to conduct experiments and collect data, ethical issues surrounding animal experimentation, and other challenges commonly faced by scientists; a representative listing of various student responses for each theme is given in S8 Text. 83% of students also indicated on the survey that they felt that the activity helped them to improve in one or more skills relevant to scientific inquiry, which included critical thinking, evaluating data, and working in groups.

**Fig 4:**
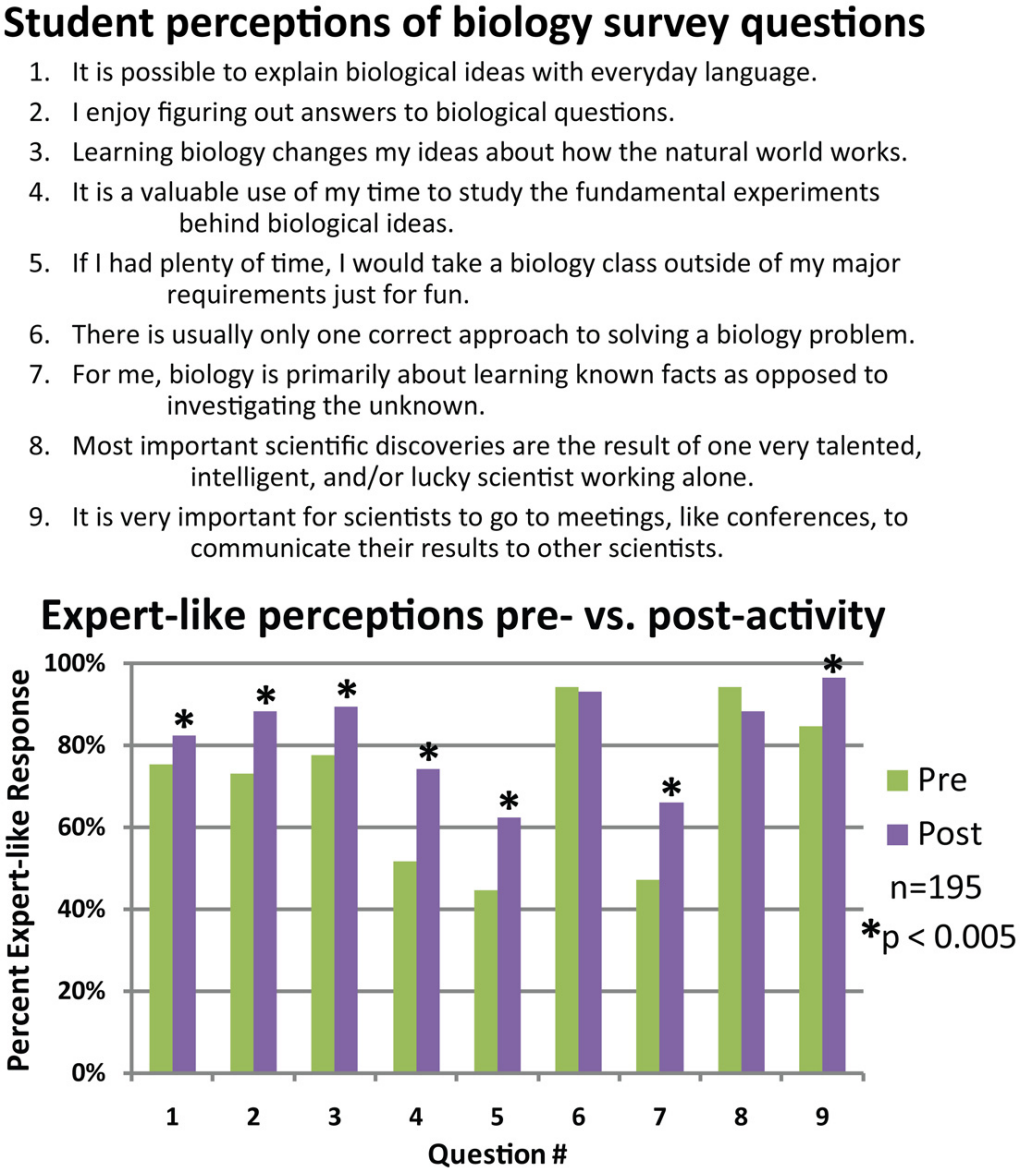
Changes in student perceptions about biology. Student survey data were collected in five independent sections of Introduction to Biology over two years (*n* = 195). Students were given a nine-question survey (upper panel) immediately before and following the activity. Questions 1–7 were selected for relevance from the CLASS-Bio survey [8], while questions 8 and 9 were added to address issues that are specifically relevant to the activity but not present in CLASS-Bio. Expert responses are affirmative for questions 1–5 and 9, and negative for questions 6–8. Shifts in student responses were analyzed using a Wilcoxon signed-rank test. Individual test results with *p*-values less than 0.005 are indicated. See S8 Text for additional details regarding data collection and analysis.

In a second survey (S7 Text), we also assessed changes in self-reported perceptions of biology as a result of the activity in five independent sections of the course Introduction to Biology at a small, selective liberal arts college (see S8 Text for details) across two years (2012 and 2013). Students were given a nine-question survey, with seven questions selected from the CLASS-Bio survey and two researcher-generated questions, immediately before and following the activity [8]. The CLASS-Bio survey rates student perceptions of biology by scoring students’ ability to give “expert-like” answers, as compared to science practitioners [8]. Notably, student perceptions of biology, as measured by the survey, improved on seven of nine questions (Fig 4). For the two questions for which there were no improvements, more than 90% of students gave the expert response in the pre-survey, indicating that ceiling effects may explain the lack of a statistically significant change, i.e., the students in this cohort already held expert-like perceptions related to these two statements prior to administering the activity [10]. See S8 Text for additional details regarding assessments.

## Discussion

### Practical Considerations in Activity Design and Educational Themes

We intentionally designed *An Inexplicable Disease* to enable the broadest possible use among life sciences instructors. Early adopters have commented favorably regarding the ease of use of the activity materials, which include a detailed description of the activity and PowerPoint presentations with usage notes (S1 Text, S1 and S2 PowerPoints). The activity was kept as short as possible to facilitate implementation in courses where the use of lengthy case studies is often hindered due to the pressures experienced by instructors to cover large amounts of canonical material. Additionally, the activity was intentionally written at the level of introductory or non-majors biology with flexible content learning goals to enable broad use in a variety of courses in the biological sciences.

The activity explicitly emphasizes the importance of specialization, collaboration, and communication in science. Specifically, the groups are specialized and investigation options intentionally focus on involving scientists with divergent specializations. Additionally, by concluding with a jigsaw, the activity emphasizes cooperation by students, acting as scientists, to solve a scientific mystery. The activity also raises other practical and ethical issues that are often unappreciated by students, such as the challenges of limited time and money to conduct research and the ethical issues involved with human and animal research. In doing so, the activity addresses four of six core competencies outlined in the *Vision and Change* report, as noted earlier [4].

One additional important aspect in designing this case was that we purposely avoided a common “preordained destiny” aspect of case studies in which every step reveals information that deliberately leads students to the correct answer. The activity was intentionally written to simulate the actual experiences of the original investigators as accurately as possible. As such, in what we term an “authentic inquiry approach” to case writing, the data is as real as possible, which means it does not always help the student, and, in fact, not all investigation options are successful. Like a real investigation, much of the data is unhelpful but must still be considered. We hope the activity can serve as a useful model for creating additional, authentic, inquiry-driven activities by others in the future, both within and outside the life sciences.

### Impact on Student Perceptions of Science

Changes in student perceptions of science are typically quantified using pre- and post-surveys or interviews of students administered at the beginning and end of a full-semester or year-long course [7,8,11,12]. In contrast, here we measured changes in self-reported student perceptions of biology as the result of only a single, short, course intervention. This distinction is important because short interventions can be more easily and widely adopted by practitioners than whole-course approaches. The seven statements selected from the CLASS-Bio survey span five of the seven different categories from the survey; statements from all five of these categories saw improvements as a result of the activity. The greatest improvements were in statements 4, 5, and 7 (Fig 4), which fell into the following four categories from the original survey: Real World Connection, Enjoyment (Personal Interest), Problem Solving: Reasoning, and Conceptual Connections/Memorization. Shifts in student responses to two questions (Fig 4, questions 4 and 7) were particularly exciting to us because they represented large shifts in student understanding of science as a process of experimentation rather than a collection of facts to be known or memorized, a significant issue that has been cited as a primary cause of student attrition from science programs during college [1]. Additionally, improvements in opinions toward biology were perhaps best demonstrated by the relatively large shift in response to question 5, which asked students whether they would like to take another biology course outside their major for fun, indicating that the activity may improve student “buy-in” in life science courses. Future assessments of the activity could further explore and define its efficacy as a teaching tool. For example, subsequent studies with greater numbers of students and courses could consider course, gender, or other demographics as random effects to explore how students from different backgrounds and/or courses respond to the activity. Future use of a full, validated instrument like the full CLASS-Bio survey itself would enable more direct comparisons of this and other case study interventions to one another or to traditional teaching approaches. Likewise, a more involved research design using student pre- and post-interviews to investigate student understanding of the scientific process would reveal greater detail as to the effectiveness of this activity in promoting learning.

### Facilitating Student Participation in Active Learning and Group Work

Like many instructors, we have often experienced difficulty motivating introverted students to participate in active learning exercises, particularly those involving group work. While beneficial to all students, active learning is perhaps even more important for underrepresented minority students [5,13,14]. We think it is worth noting that we have received significant feedback from multiple instructors who have adopted the activity in their courses, commenting that it was particularly useful as an introduction for their students to active learning and group work. Encouragingly, many students expressed similar sentiments in the open-comment section of our survey. As one student elaborated: “*It brought me out of my shell and pushed me to do some critical thinking and brainstorming while in a group setting*.”

## Conclusion

We hope that, because of broad course applicability and ease of adoption, the activity will be beneficial to instructors in a wide array of life science courses. Student responses from our surveys indicated that an important aspect of the activity was its efficacy in creating experiences in which students felt like they were experiencing scientific inquiry first-hand, which includes substantial confusion and frustration in the course of scientific exploration. Rather than protecting students from confusing material, we think that allowing students to experience the profound frustration of discovery actually improved their opinion of biology, which they better understood to be more about a process than known facts. One strength of this particular activity is that it can be accomplished in a lecture, rather than requiring a laboratory. Students responded to this combined “choose-your-own-experiment” and “authentic inquiry” approach with unexpected enthusiasm; perhaps one student summed up student response to the activity best by exclaiming: “*It was* *cool* *to be Gregory House for a day*!”

## IRB Statement

Approval to evaluate student survey responses (exempt status) was granted by the Institutional Review Boards of each of the institutions described herein.

## Supporting Information

S1 PowerPoint Part I PowerPoint presentation. Notes are included with each slide containing recommendations for presentation and additional information regarding the actual case.
(PPT)

S2 PowerPoint Part II PowerPoint presentation. Notes are included with each slide containing recommendations for presentation and additional information regarding the actual case.
(PPT)

S1 Text Description of the activity for instructors. To be read by the instructor only.
(PDF)

S2 Text Rules and guidelines handout. To be given to students prior to the activity (one per student).
(PDF)

S3 Text Group packet (physicians). To be handed out at the beginning of the activity (one per group; optimally equal number of groups with each type).
(PDF)

S4 Text Group packet (anthropologists). To be handed out at the beginning of the activity (one per group; optimally equal number of groups with each type).
(PDF)

S5 Text Additional investigation sheets. To be kept by the instructor and handed out to groups during the “student investigation” phase of the activity.
(PDF)

S6 Text Post-activity written survey. To be completed after the activity at the instructor’s discretion.
(PDF)

S7 Text Pre-/Post- student opinion survey. To be completed before and after the activity at the instructor’s discretion.
(PDF)

S8 Text Supplemental Methods. Additional information regarding data collection, evaluation, and analysis.
(PDF)
