# Correction: Cannibalism, Kuru, and Mad Cows: Prion Disease As a "Choose-Your-Own-Experiment" Case Study to Simulate Scientific Inquiry in Large Lectures

**DOI:** 10.1371/journal.pbio.1002425

**Published:** 2016-03-18

**Authors:** The PLOS Biology Staff

## Notice of Republication

This article was republished on February 23, 2016 to remove or replace copyrighted material and images with privacy issues in two Supporting Information files, S1 PowerPoint and S2 PointPoint. In S2 PowerPoint, the spelling of “Stanley Prusiner” was also corrected. The publisher apologizes for the errors. Please download these files again to view the correct version. The republished, corrected files are provided here for reference.

## Supporting Information

S1 PowerPoint Republished, corrected file. (PPT)

S2 PowerPoint Republished, corrected file. (PPT)
