# Supplementary material for: Cannibalism, Kuru, and Mad Cows: Prion Disease As a “Choose-Your-Own-Experiment” Case Study to Simulate Scientific Inquiry in Large Lectures
Source: PLoS Biol. 2016 Jan 20;14(1):e1002351. doi: 10.1371/journal.pbio.1002351 (PMC4720379; doi:10.1371/journal.pbio.1002351)
Supplement: S1 PowerPoint — Notes are included with each slide containing recommendations for presentation and additional information regarding the actual case. (PPT) [file pbio.1002351.s001.ppt]

## Slide 1
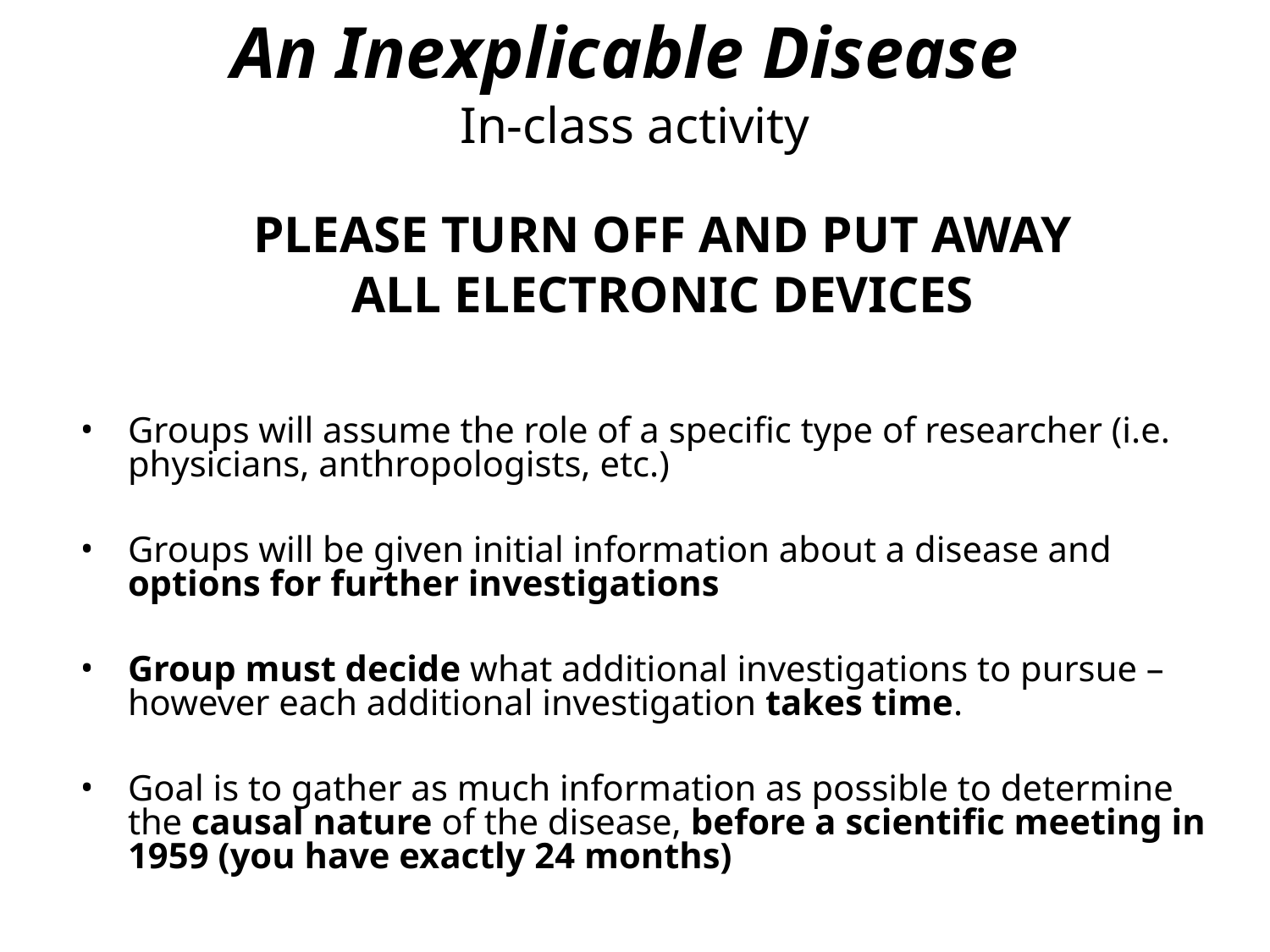

An Inexplicable Disease
In-class activity
# PLEASE TURN OFF AND PUT AWAY
ALL ELECTRONIC DEVICES
Groups will assume the role of a specific type of researcher (i.e. physicians, anthropologists, etc.)
Groups will be given initial information about a disease and options for further investigations
Group must decide what additional investigations to pursue – however each additional investigation takes time.
Goal is to gather as much information as possible to determine the causal nature of the disease, before a scientific meeting in 1959 (you have exactly 24 months)

## Slide 2
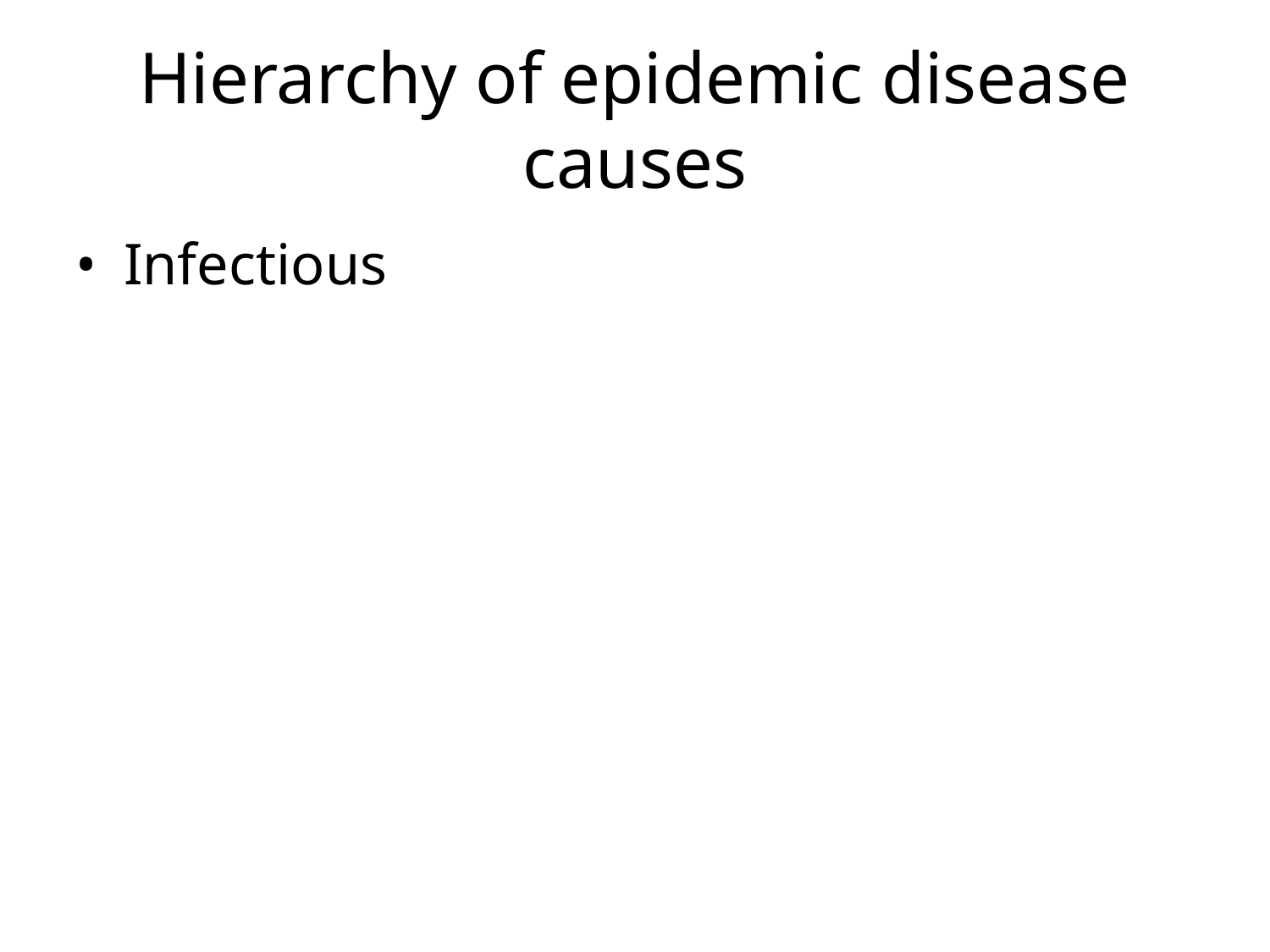

# Hierarchy of epidemic disease causes
Infectious

## Slide 3
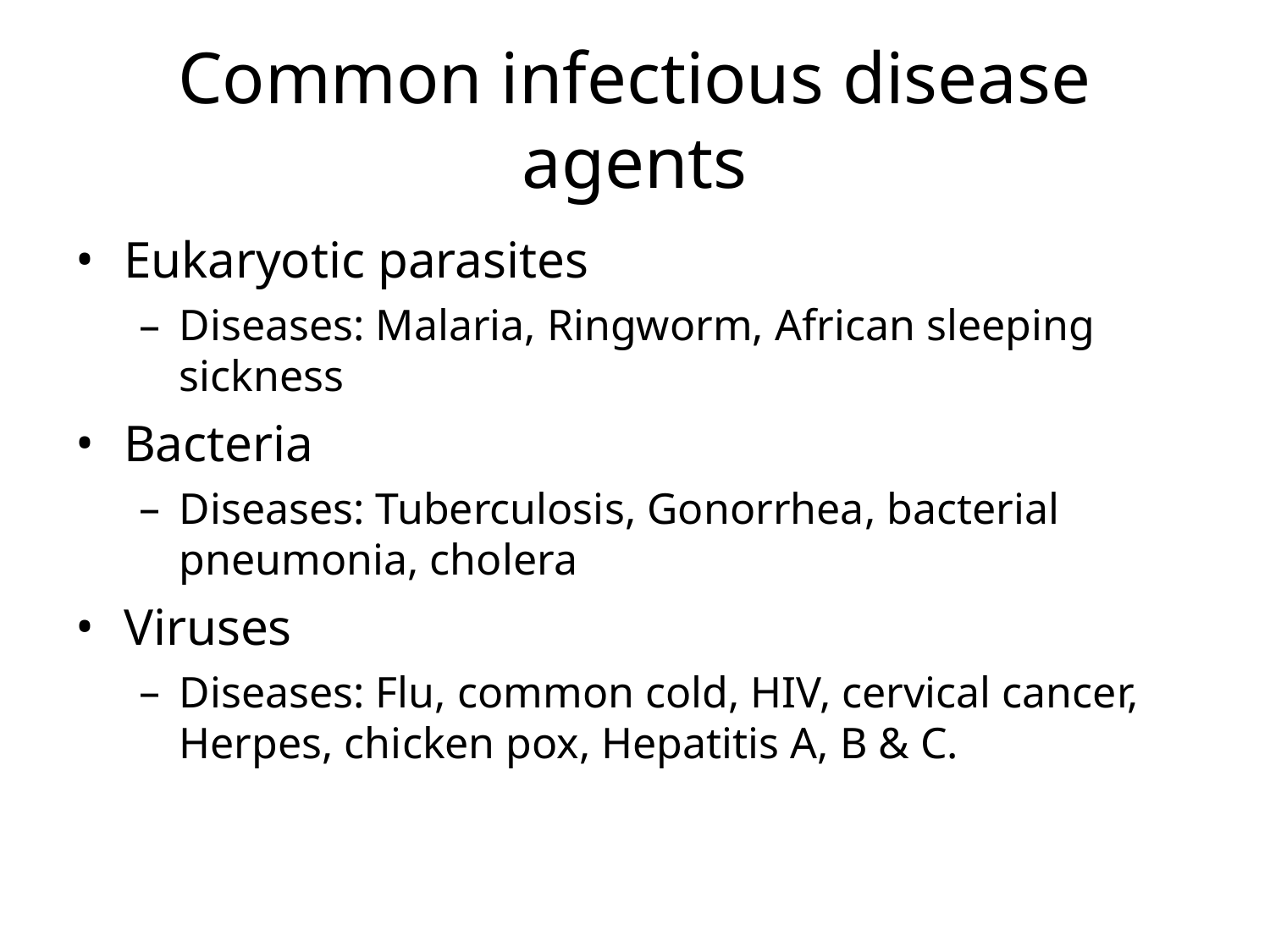

# Common infectious disease agents
Eukaryotic parasites
Diseases: Malaria, Ringworm, African sleeping sickness
Bacteria
Diseases: Tuberculosis, Gonorrhea, bacterial pneumonia, cholera
Viruses
Diseases: Flu, common cold, HIV, cervical cancer, Herpes, chicken pox, Hepatitis A, B & C.

## Slide 4
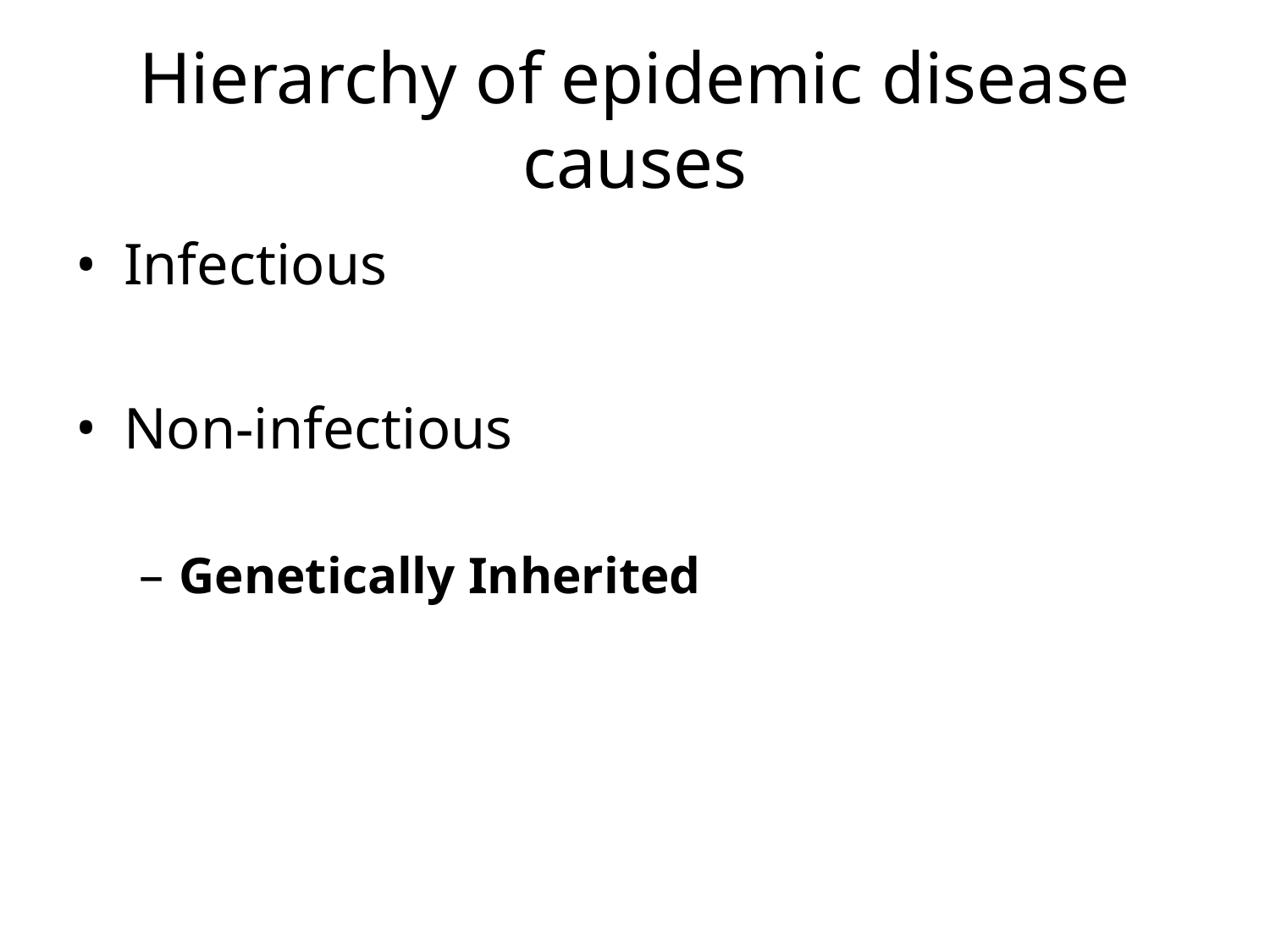

# Hierarchy of epidemic disease causes
Infectious
Non-infectious
Genetically Inherited

## Slide 5
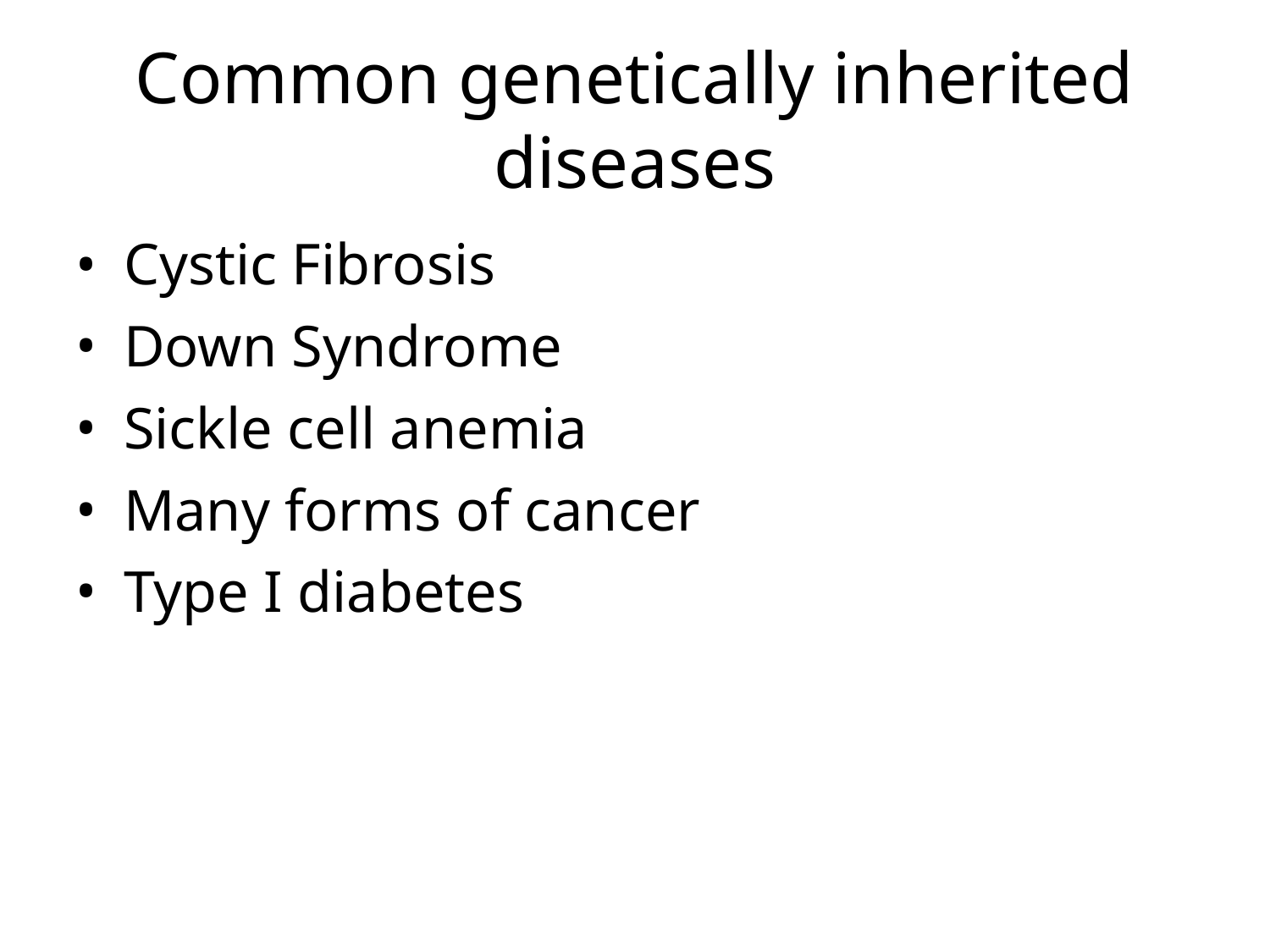

# Common genetically inherited diseases
Cystic Fibrosis
Down Syndrome
Sickle cell anemia
Many forms of cancer
Type I diabetes

## Slide 6
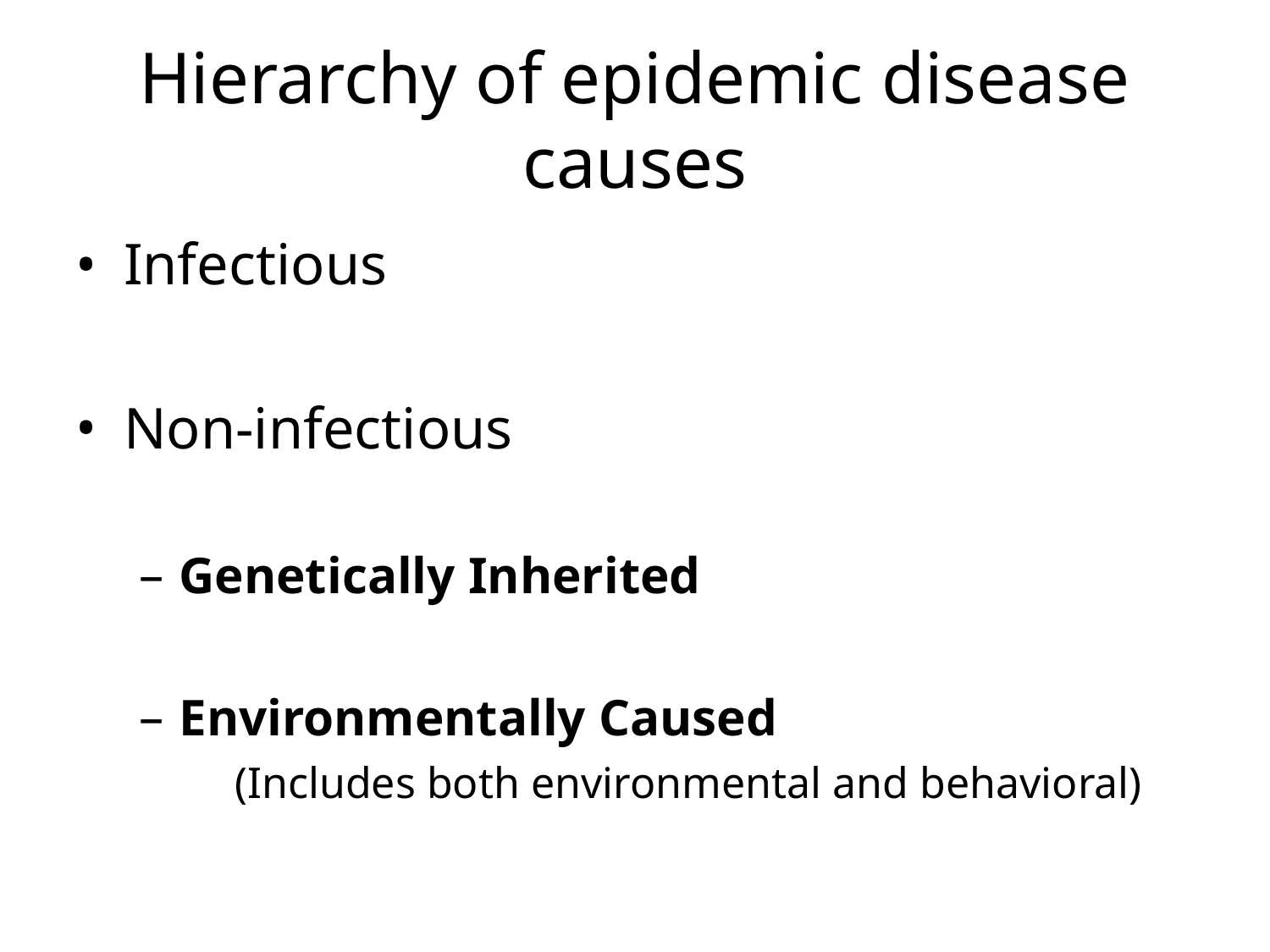

# Hierarchy of epidemic disease causes
Infectious
Non-infectious
Genetically Inherited
Environmentally Caused
(Includes both environmental and behavioral)

## Slide 7
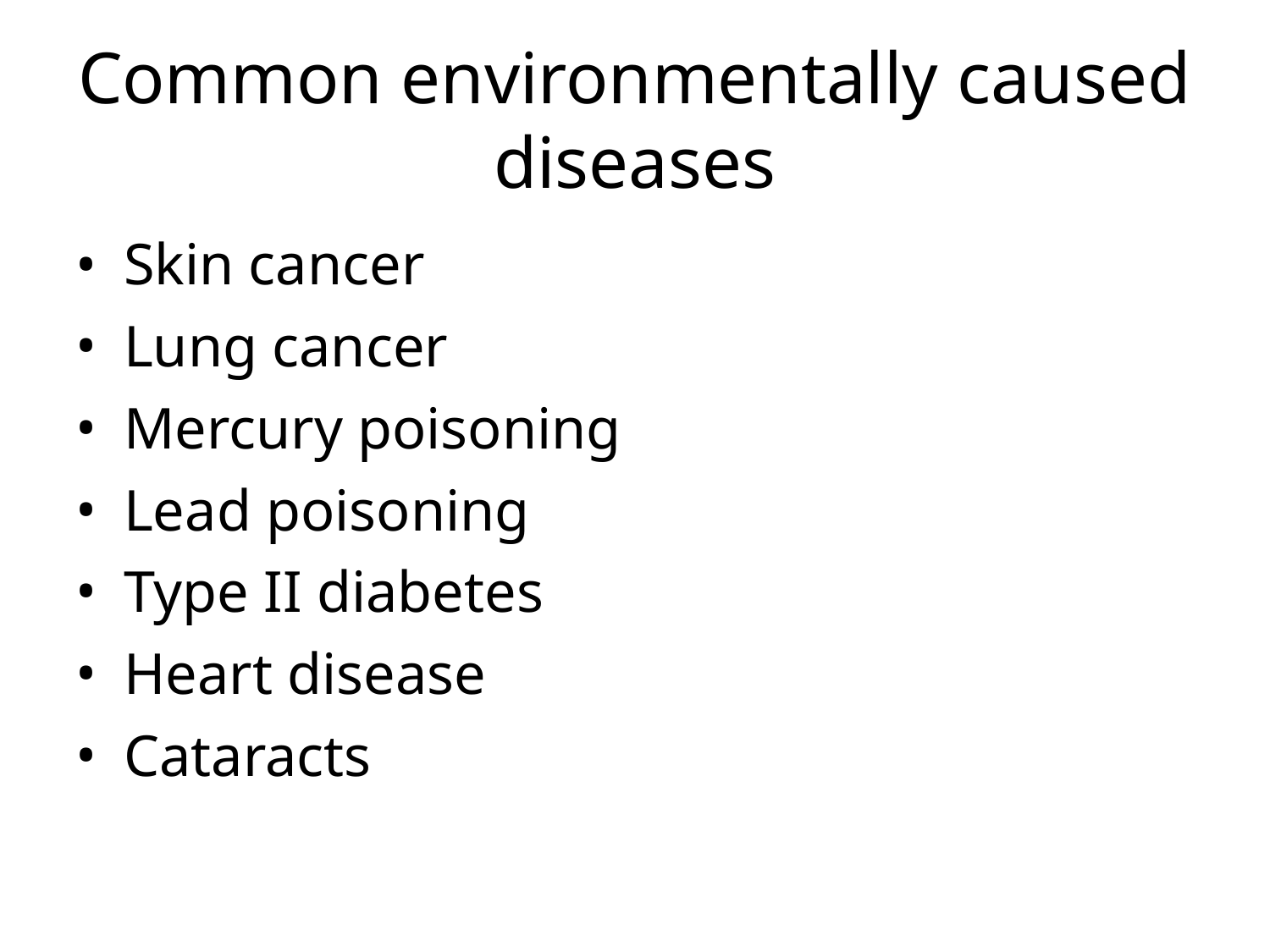

# Common environmentally caused diseases
Skin cancer
Lung cancer
Mercury poisoning
Lead poisoning
Type II diabetes
Heart disease
Cataracts

## Slide 8
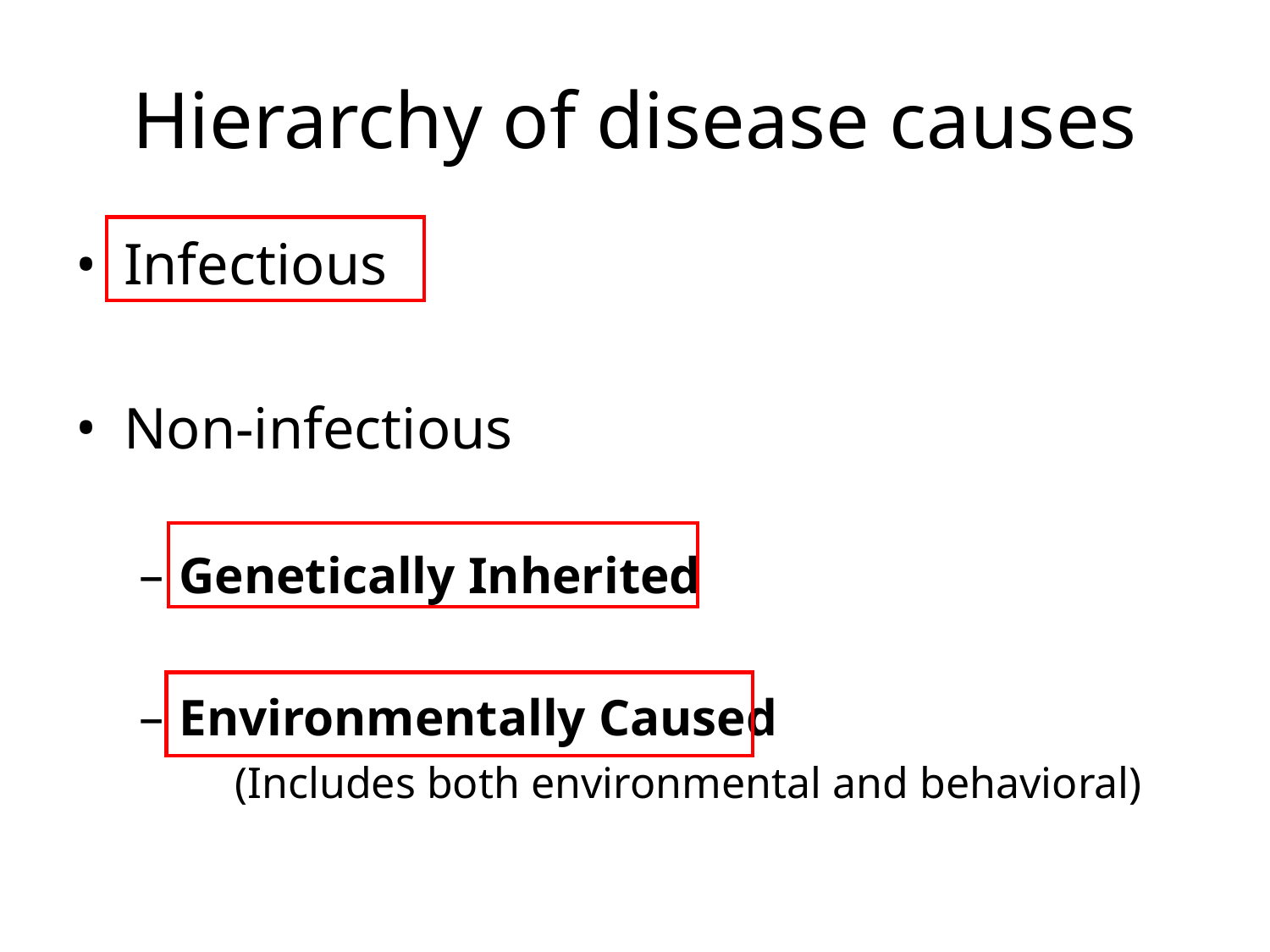

# Hierarchy of disease causes
Infectious
Non-infectious
Genetically Inherited
Environmentally Caused
(Includes both environmental and behavioral)

## Slide 9
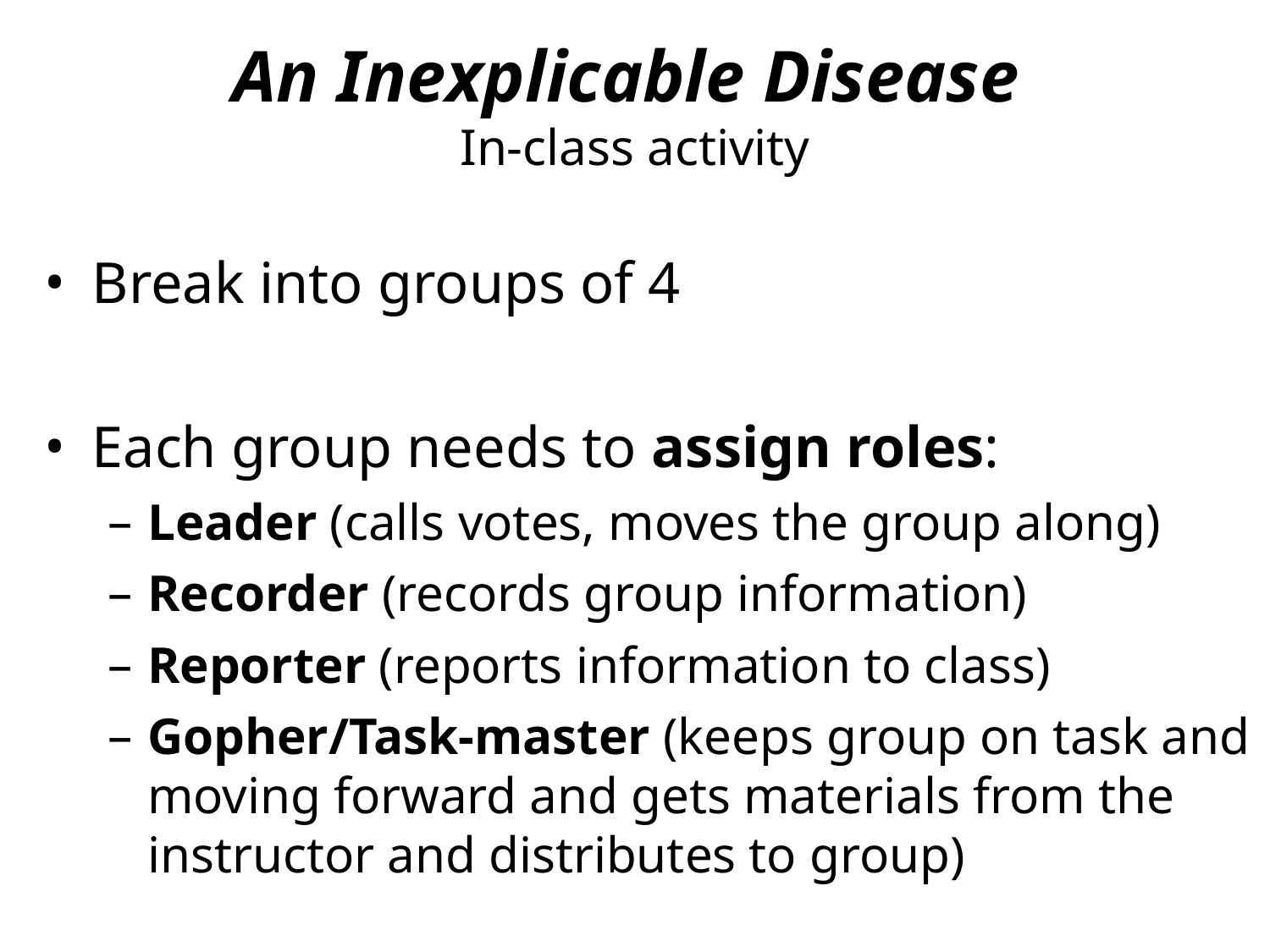

# An Inexplicable Disease In-class activity
Break into groups of 4
Each group needs to assign roles:
Leader (calls votes, moves the group along)
Recorder (records group information)
Reporter (reports information to class)
Gopher/Task-master (keeps group on task and moving forward and gets materials from the instructor and distributes to group)

## Slide 10
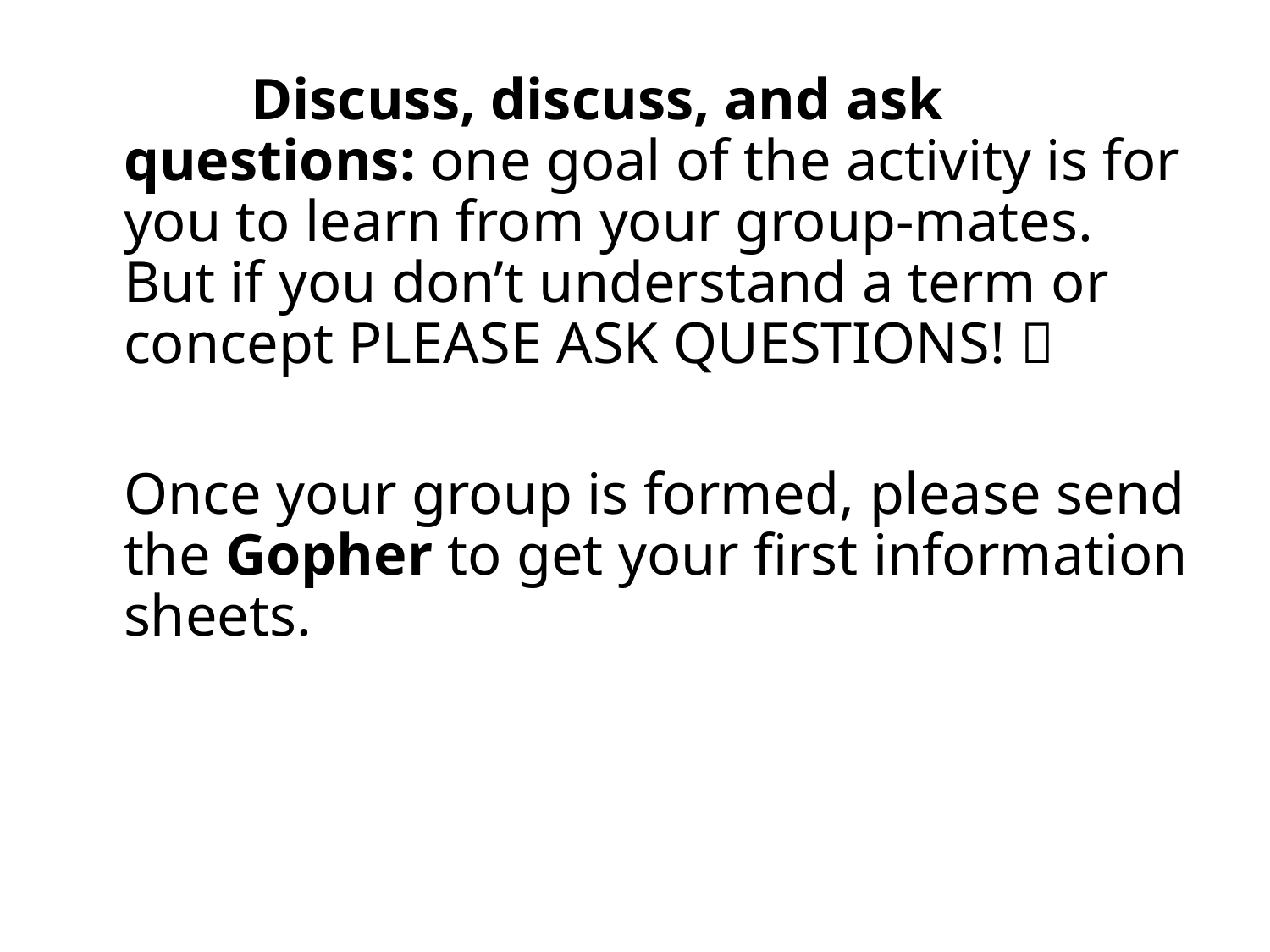

# Discuss, discuss, and ask questions: one goal of the activity is for you to learn from your group-mates. But if you don’t understand a term or concept PLEASE ASK QUESTIONS! 
Once your group is formed, please send the Gopher to get your first information sheets.

## Slide 11
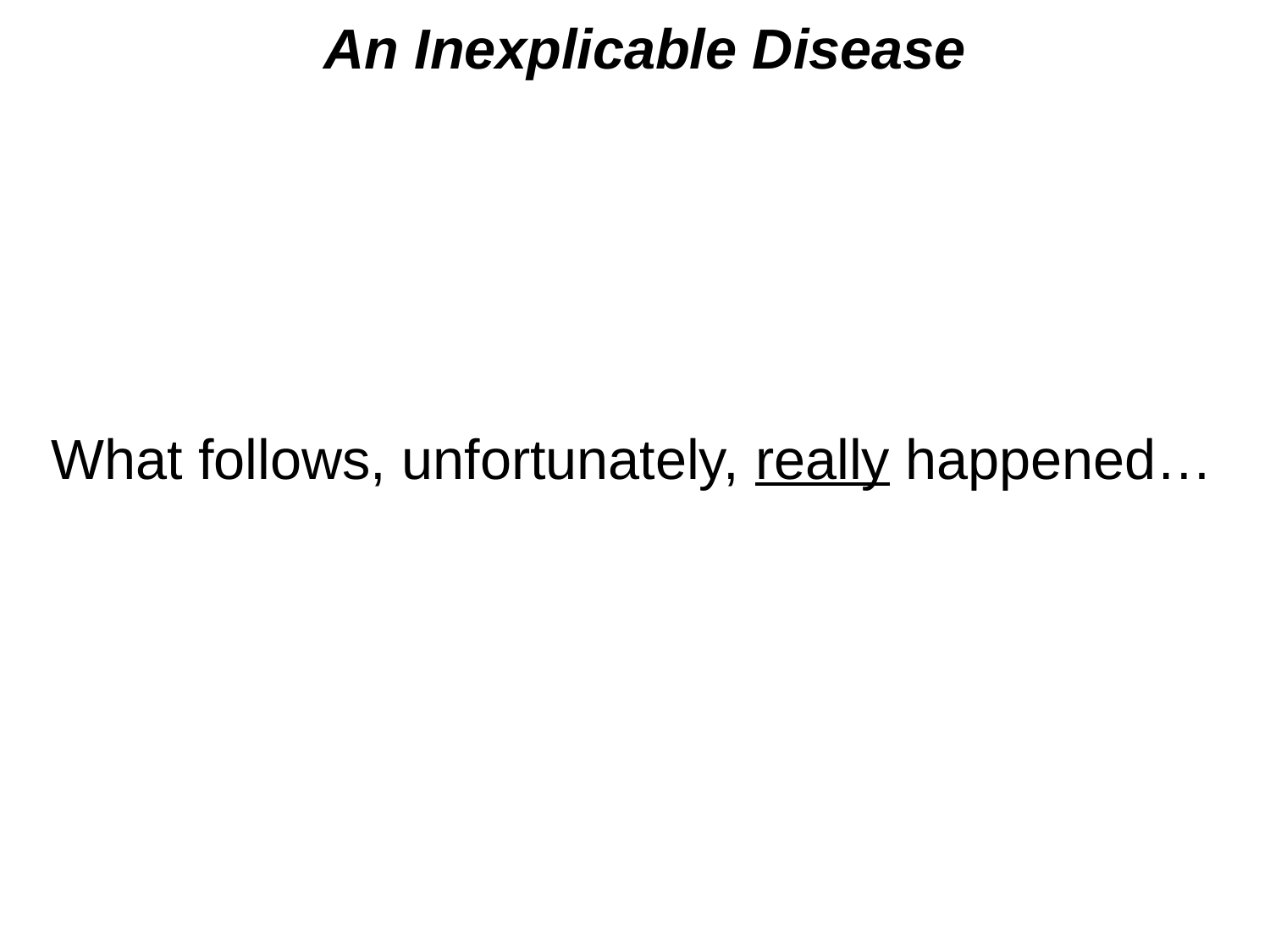

# An Inexplicable Disease
What follows, unfortunately, really happened…

## Slide 12
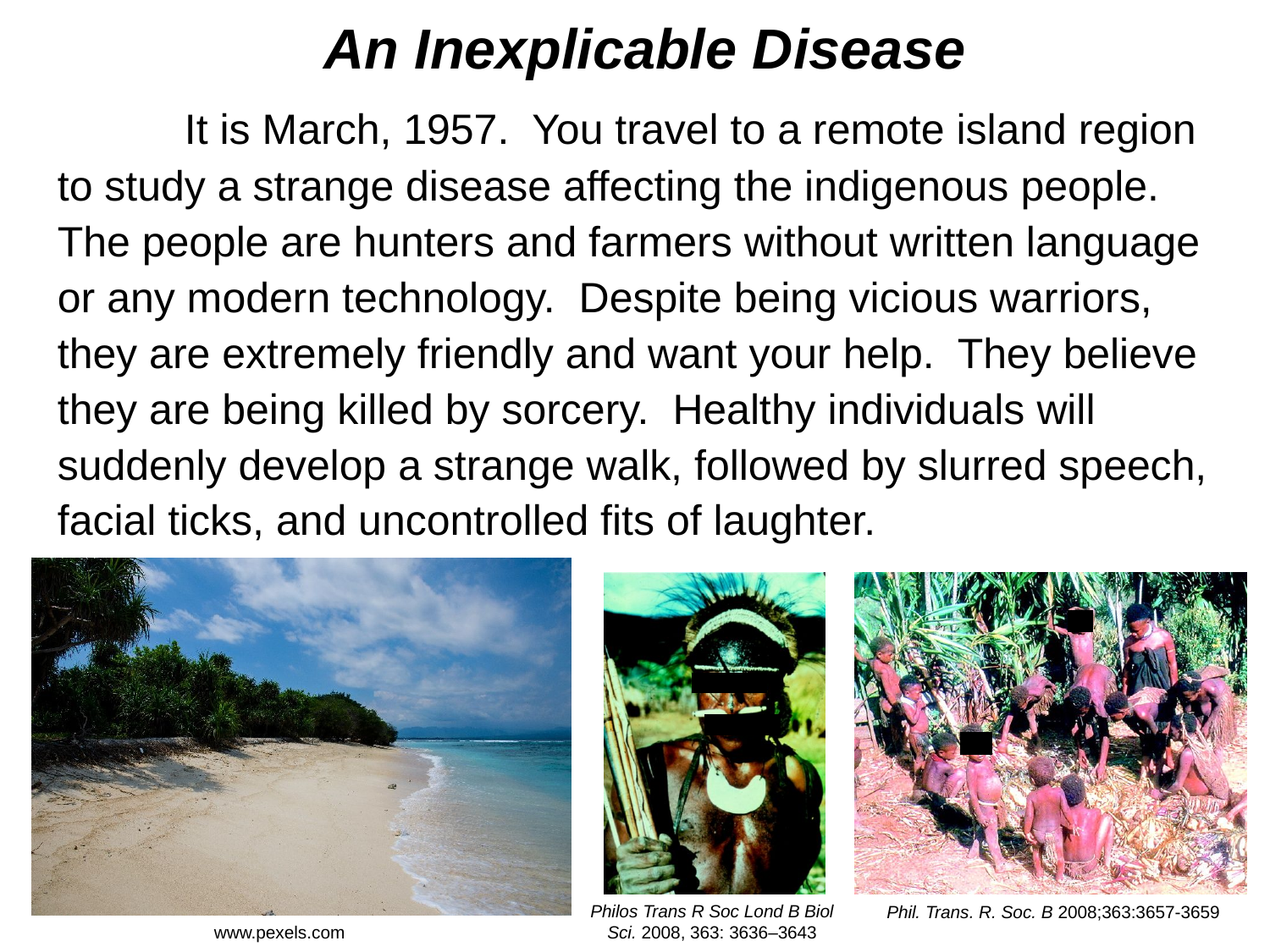

# An Inexplicable Disease
	It is March, 1957. You travel to a remote island region to study a strange disease affecting the indigenous people. The people are hunters and farmers without written language or any modern technology. Despite being vicious warriors, they are extremely friendly and want your help. They believe they are being killed by sorcery. Healthy individuals will suddenly develop a strange walk, followed by slurred speech, facial ticks, and uncontrolled fits of laughter.
Philos Trans R Soc Lond B Biol Sci. 2008, 363: 3636–3643
Phil. Trans. R. Soc. B 2008;363:3657-3659
www.pexels.com

## Slide 13
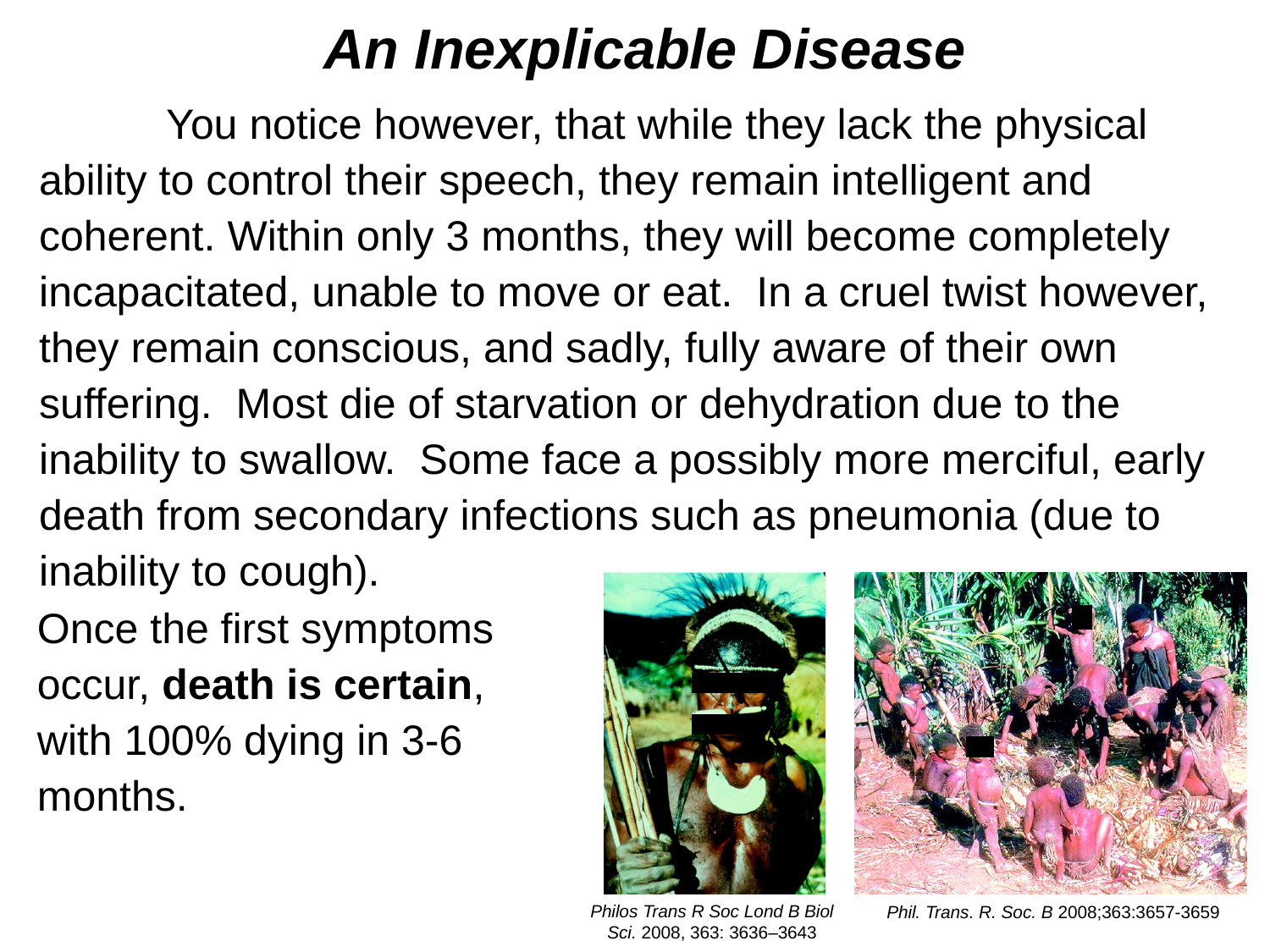

# An Inexplicable Disease
	You notice however, that while they lack the physical ability to control their speech, they remain intelligent and coherent. Within only 3 months, they will become completely incapacitated, unable to move or eat. In a cruel twist however, they remain conscious, and sadly, fully aware of their own suffering. Most die of starvation or dehydration due to the inability to swallow. Some face a possibly more merciful, early death from secondary infections such as pneumonia (due to inability to cough).
Once the first symptoms occur, death is certain, with 100% dying in 3-6 months.
Philos Trans R Soc Lond B Biol Sci. 2008, 363: 3636–3643
Phil. Trans. R. Soc. B 2008;363:3657-3659

## Slide 14
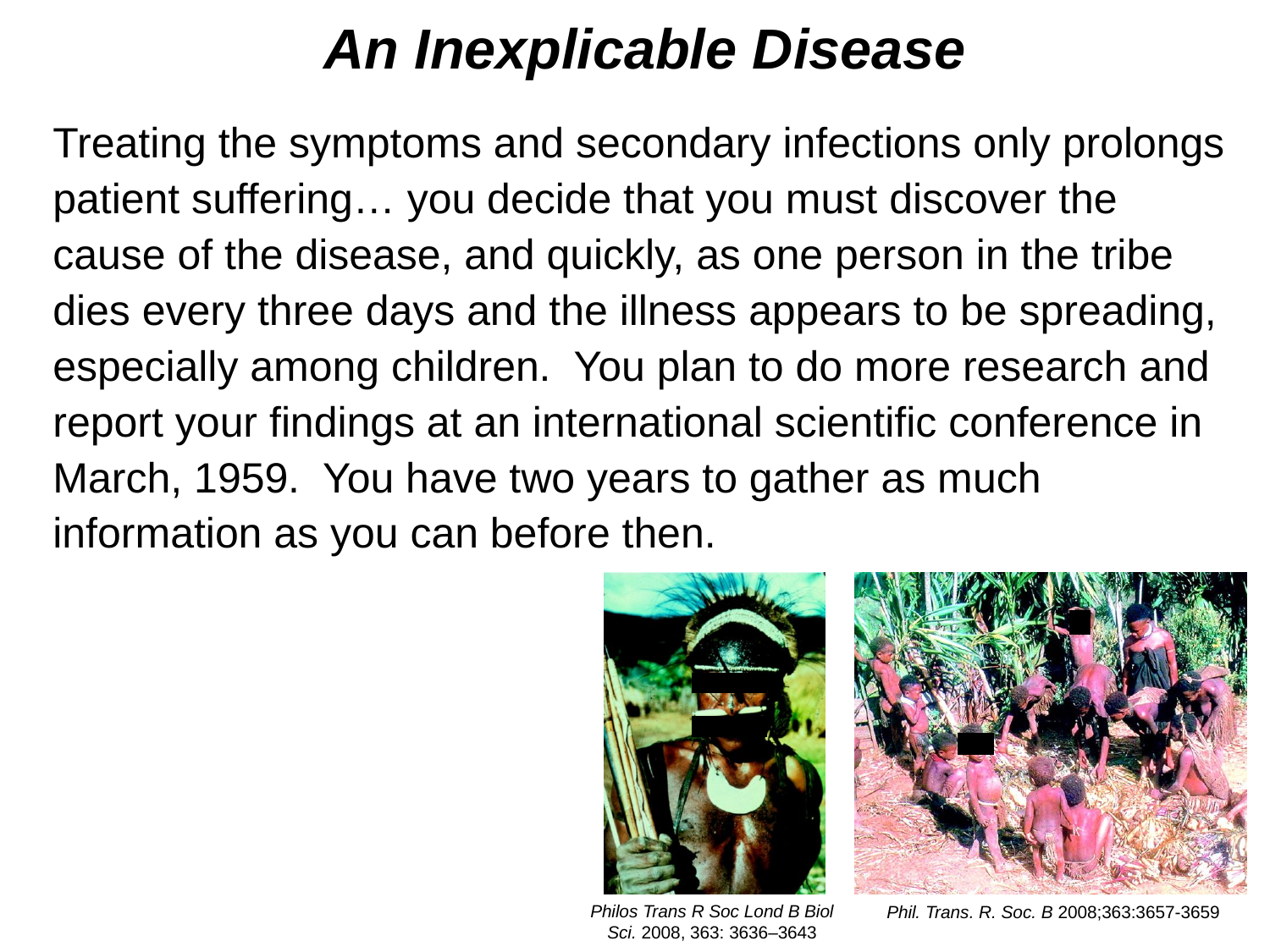

# An Inexplicable Disease
Treating the symptoms and secondary infections only prolongs patient suffering… you decide that you must discover the cause of the disease, and quickly, as one person in the tribe dies every three days and the illness appears to be spreading, especially among children. You plan to do more research and report your findings at an international scientific conference in March, 1959. You have two years to gather as much information as you can before then.
Philos Trans R Soc Lond B Biol Sci. 2008, 363: 3636–3643
Phil. Trans. R. Soc. B 2008;363:3657-3659

## Slide 15
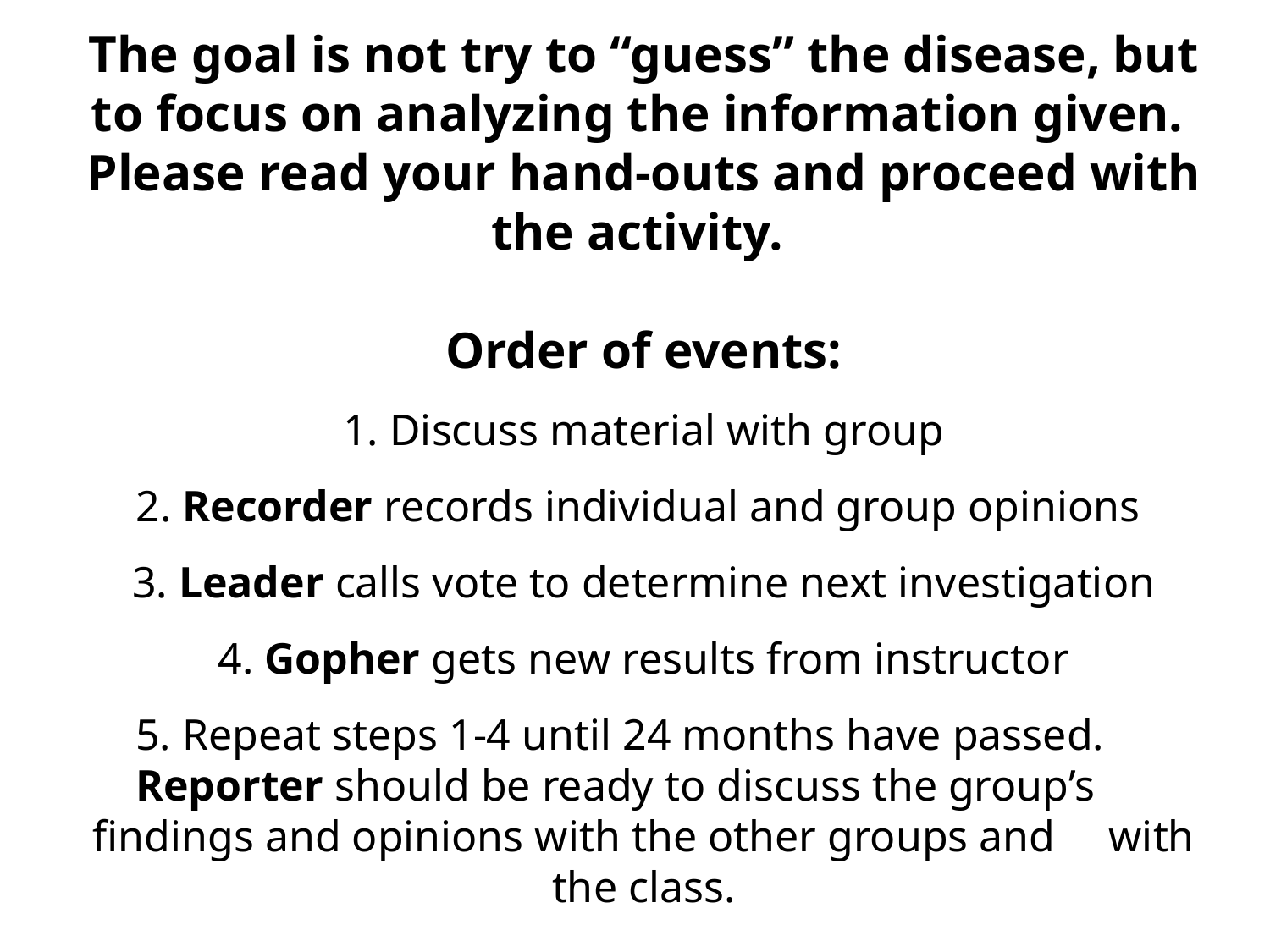

# The goal is not try to “guess” the disease, but to focus on analyzing the information given. Please read your hand-outs and proceed with the activity. Order of events: 1. Discuss material with group 2. Recorder records individual and group opinions 3. Leader calls vote to determine next investigation 4. Gopher gets new results from instructor 5. Repeat steps 1-4 until 24 months have passed. 	Reporter should be ready to discuss the group’s 	findings and opinions with the other groups and 	with the class.

## Slide 16
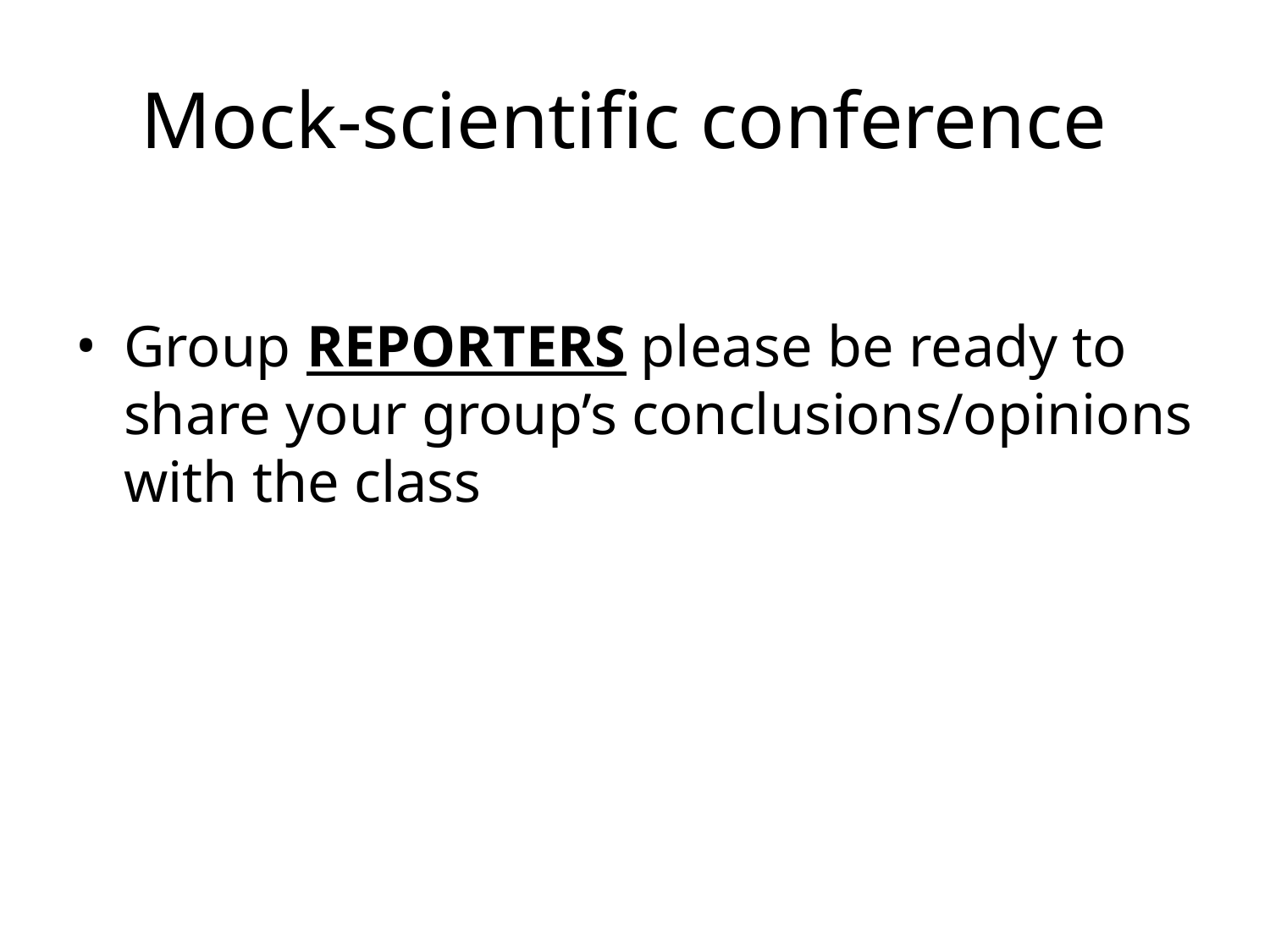

# Mock-scientific conference
Group REPORTERS please be ready to share your group’s conclusions/opinions with the class

## Slide 17
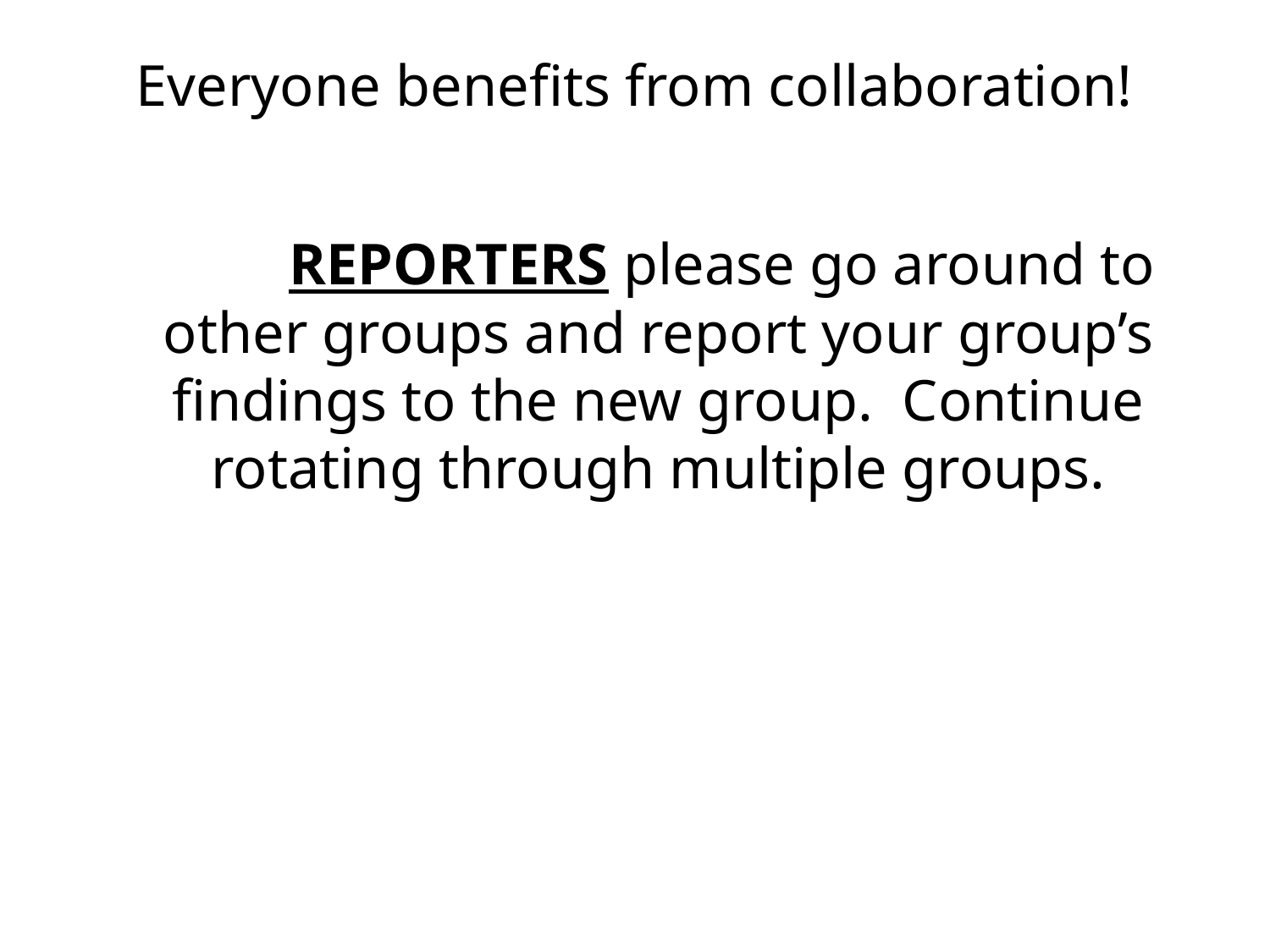

# Everyone benefits from collaboration!
	REPORTERS please go around to other groups and report your group’s findings to the new group. Continue rotating through multiple groups.

## Slide 18
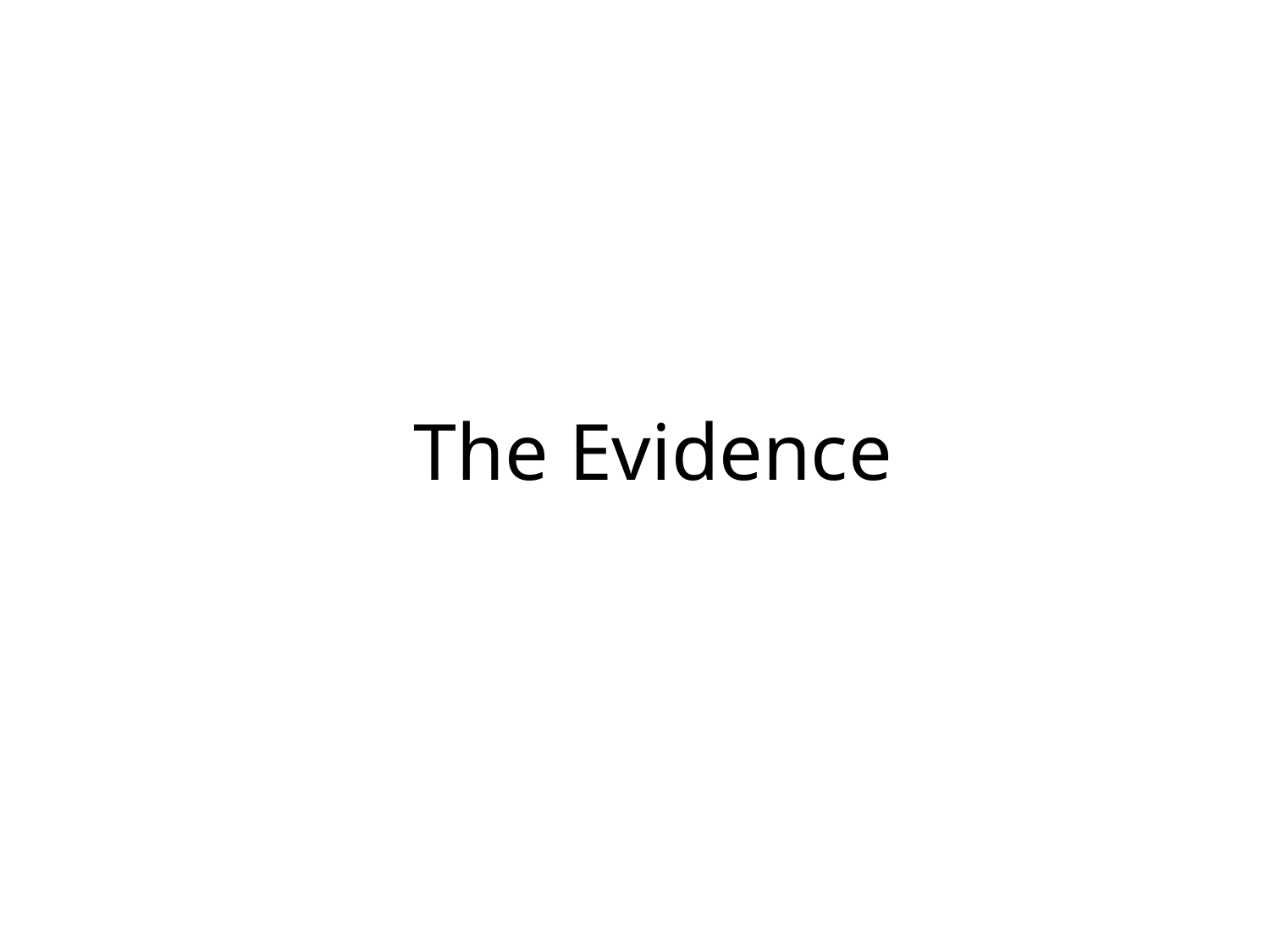

# The Evidence

## Slide 19
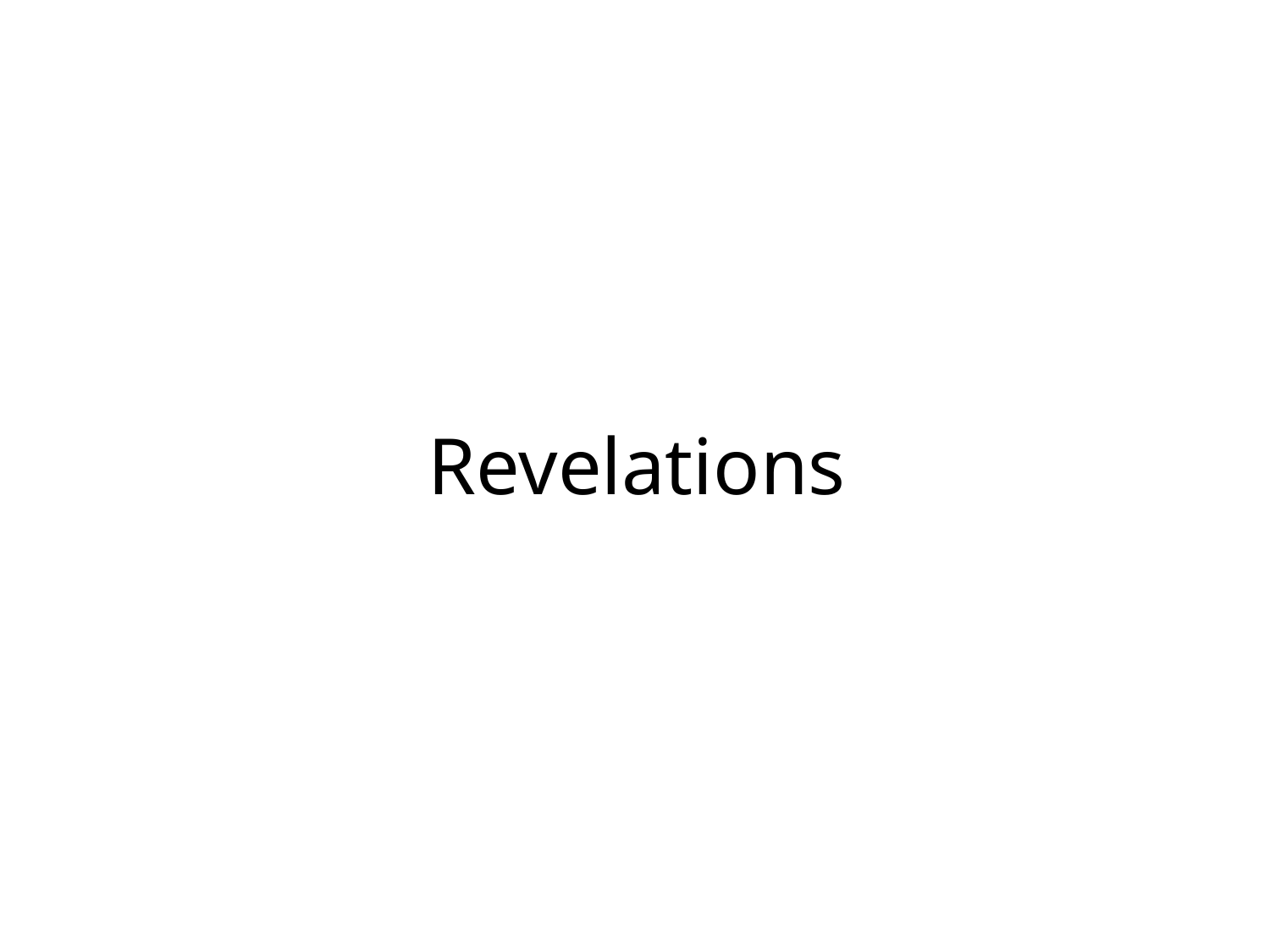

# Revelations

## Slide 20
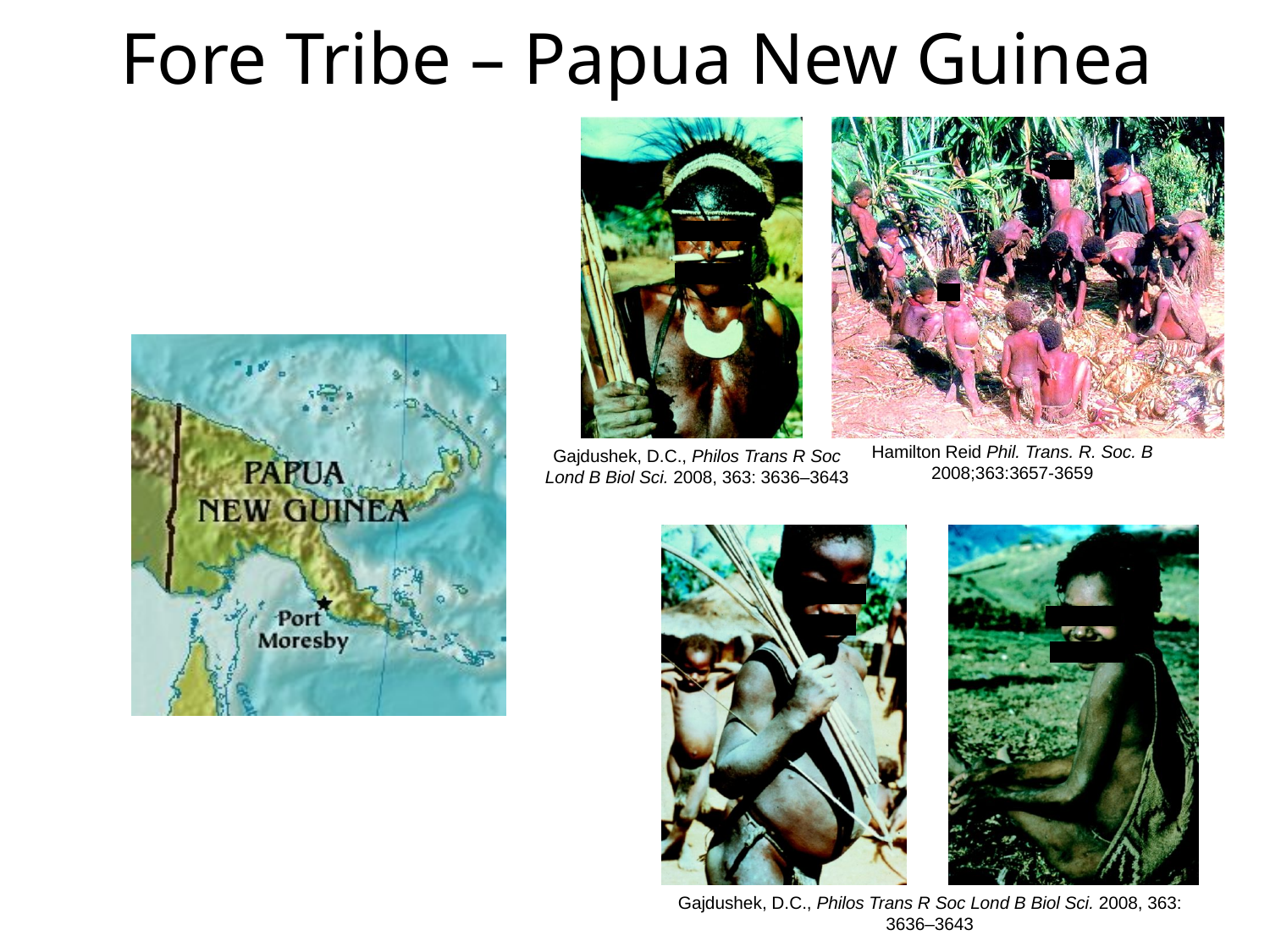

# Fore Tribe – Papua New Guinea
Gajdushek, D.C., Philos Trans R Soc Lond B Biol Sci. 2008, 363: 3636–3643
Hamilton Reid Phil. Trans. R. Soc. B 2008;363:3657-3659
Gajdushek, D.C., Philos Trans R Soc Lond B Biol Sci. 2008, 363: 3636–3643

## Slide 21
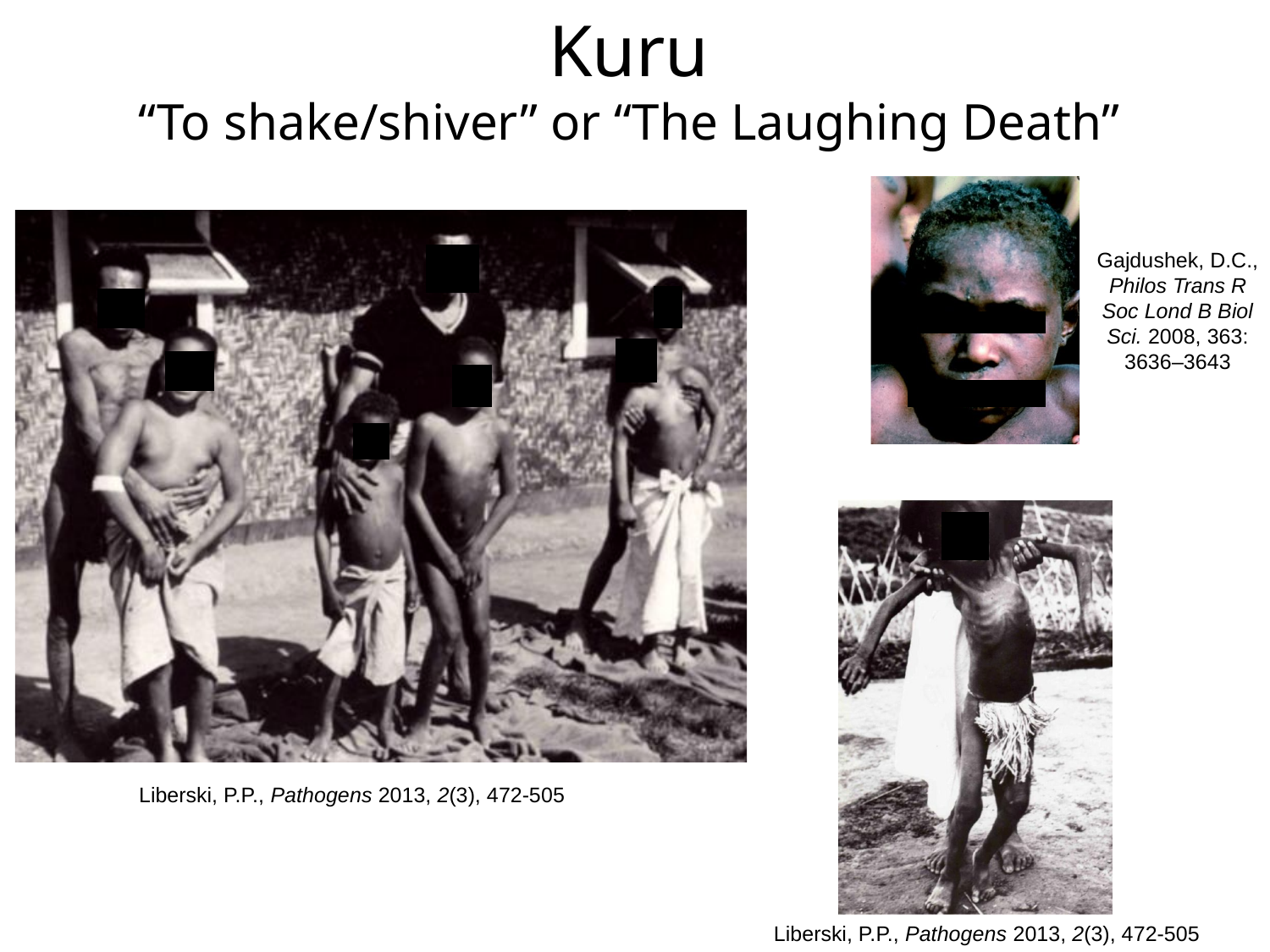

# Kuru “To shake/shiver” or “The Laughing Death”
Gajdushek, D.C., Philos Trans R Soc Lond B Biol Sci. 2008, 363: 3636–3643
Liberski, P.P., Pathogens 2013, 2(3), 472-505
Liberski, P.P., Pathogens 2013, 2(3), 472-505

## Slide 22
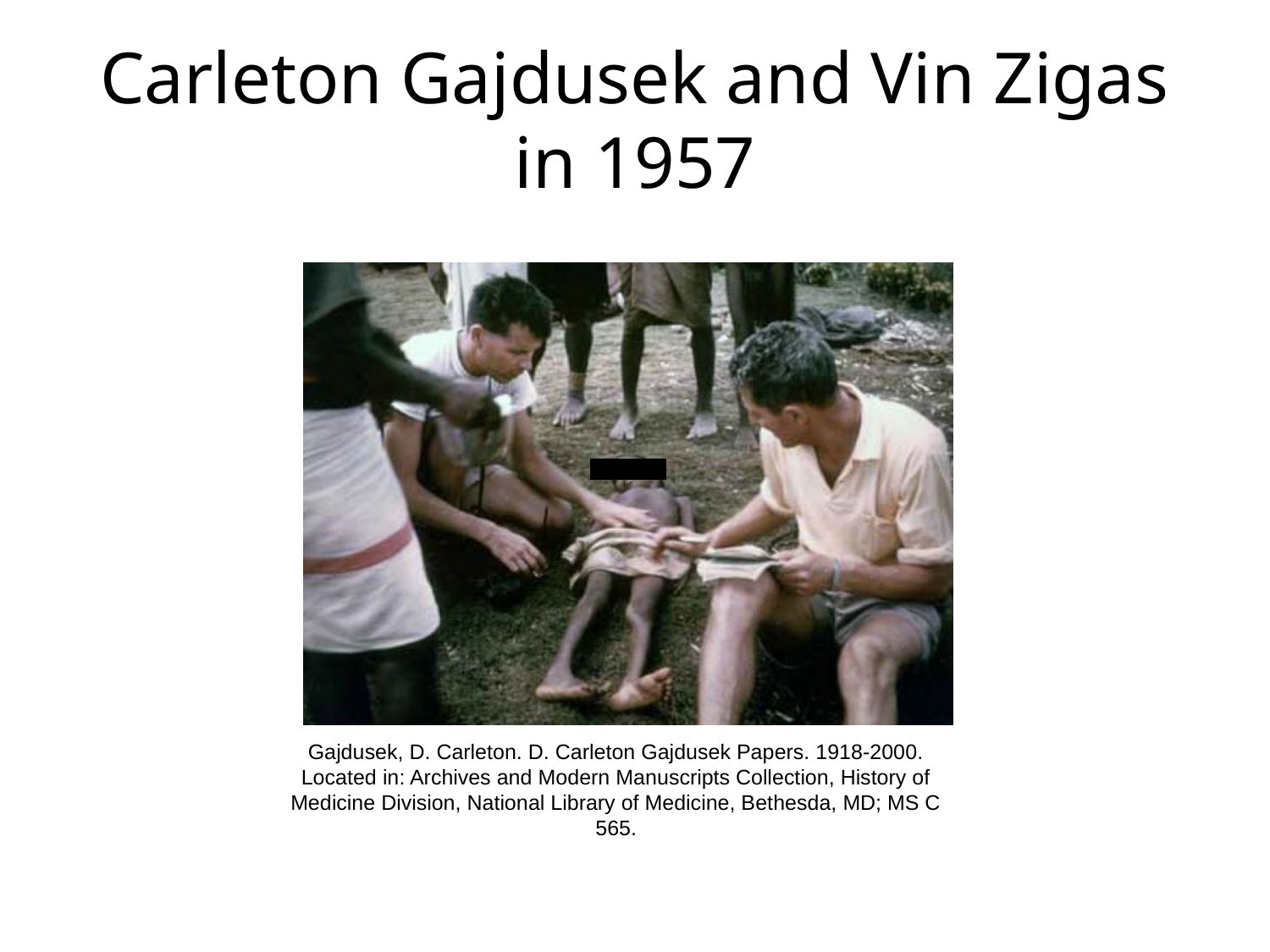

# Carleton Gajdusek and Vin Zigas in 1957
Gajdusek, D. Carleton. D. Carleton Gajdusek Papers. 1918-2000. Located in: Archives and Modern Manuscripts Collection, History of Medicine Division, National Library of Medicine, Bethesda, MD; MS C 565.

## Slide 23
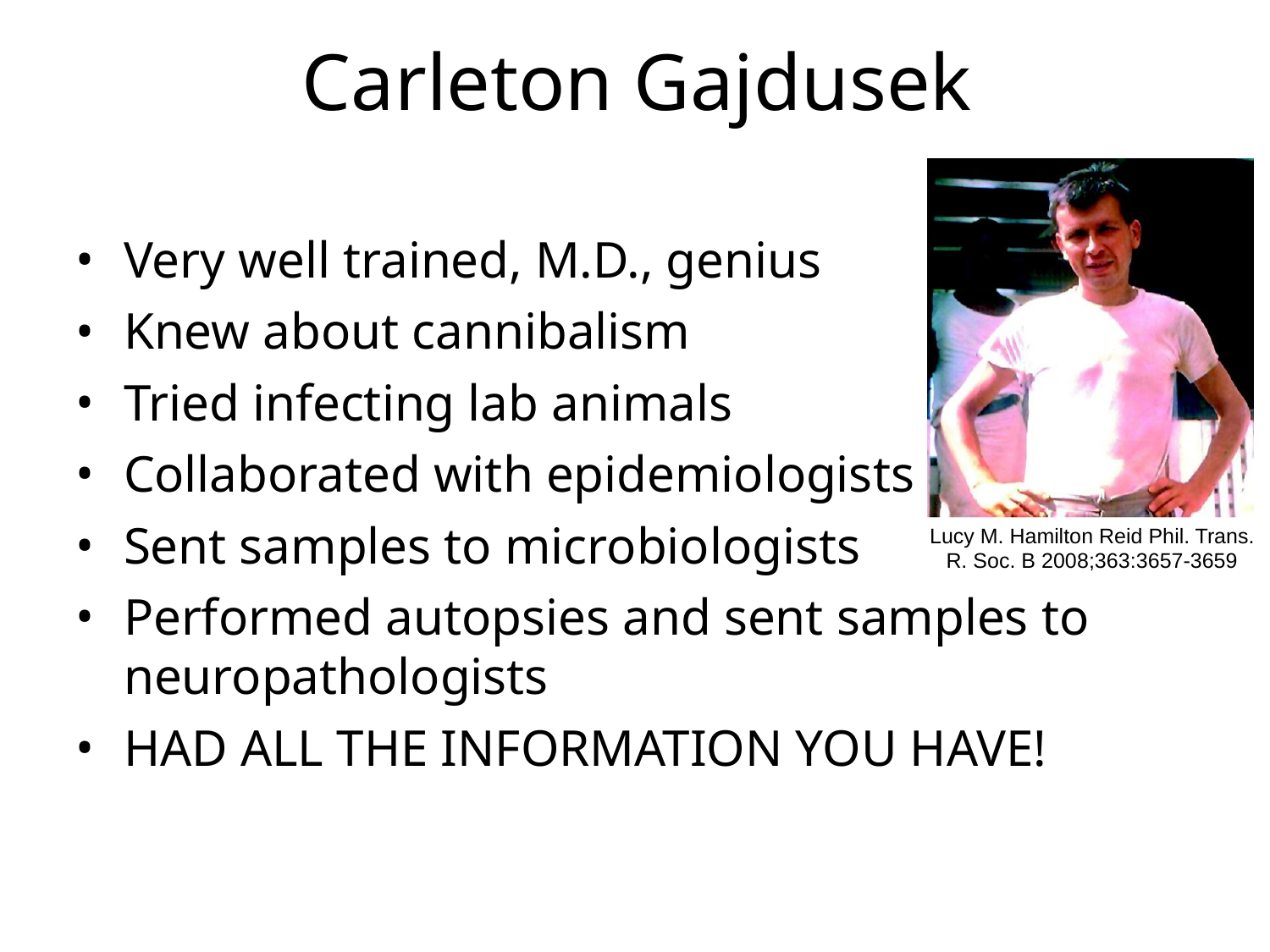

# Carleton Gajdusek
Very well trained, M.D., genius
Knew about cannibalism
Tried infecting lab animals
Collaborated with epidemiologists
Sent samples to microbiologists
Performed autopsies and sent samples to neuropathologists
HAD ALL THE INFORMATION YOU HAVE!
Lucy M. Hamilton Reid Phil. Trans. R. Soc. B 2008;363:3657-3659

## Slide 24
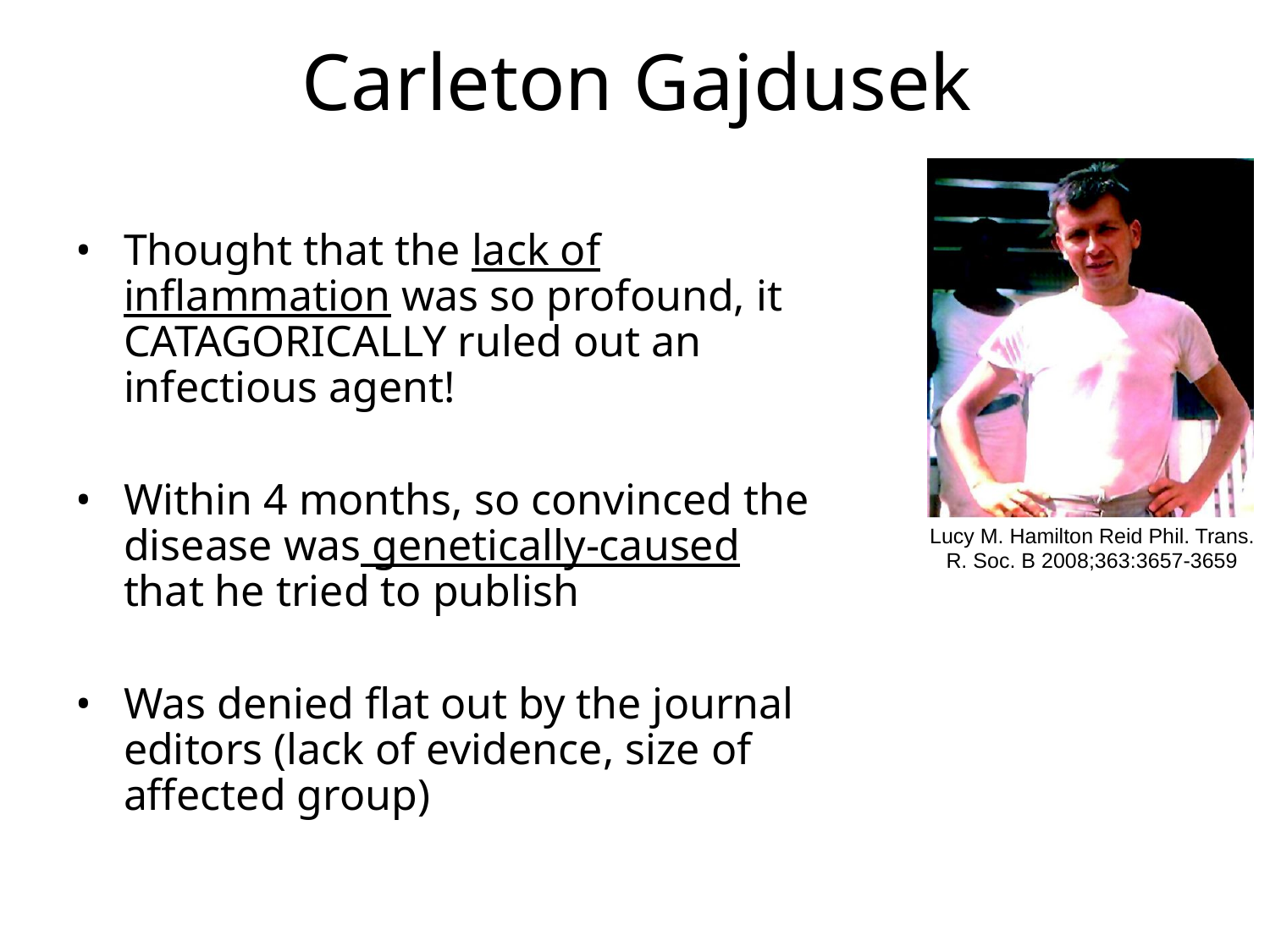

Carleton Gajdusek
# Thought that the lack of inflammation was so profound, it CATAGORICALLY ruled out an infectious agent!
Within 4 months, so convinced the disease was genetically-caused that he tried to publish
Was denied flat out by the journal editors (lack of evidence, size of affected group)
Lucy M. Hamilton Reid Phil. Trans. R. Soc. B 2008;363:3657-3659

## Slide 25
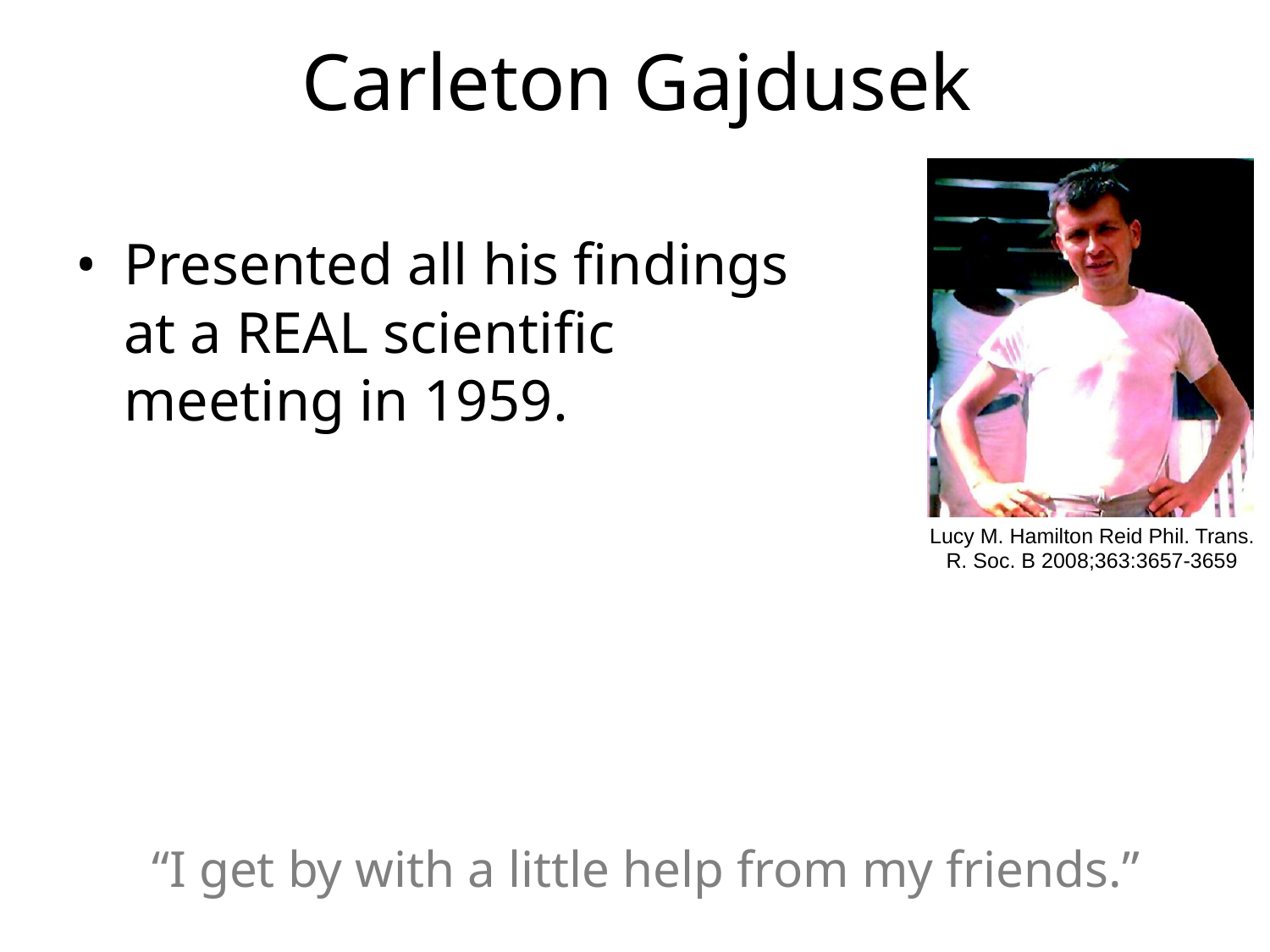

Carleton Gajdusek
# Presented all his findings at a REAL scientific meeting in 1959.
Lucy M. Hamilton Reid Phil. Trans. R. Soc. B 2008;363:3657-3659
“I get by with a little help from my friends.”

## Slide 26
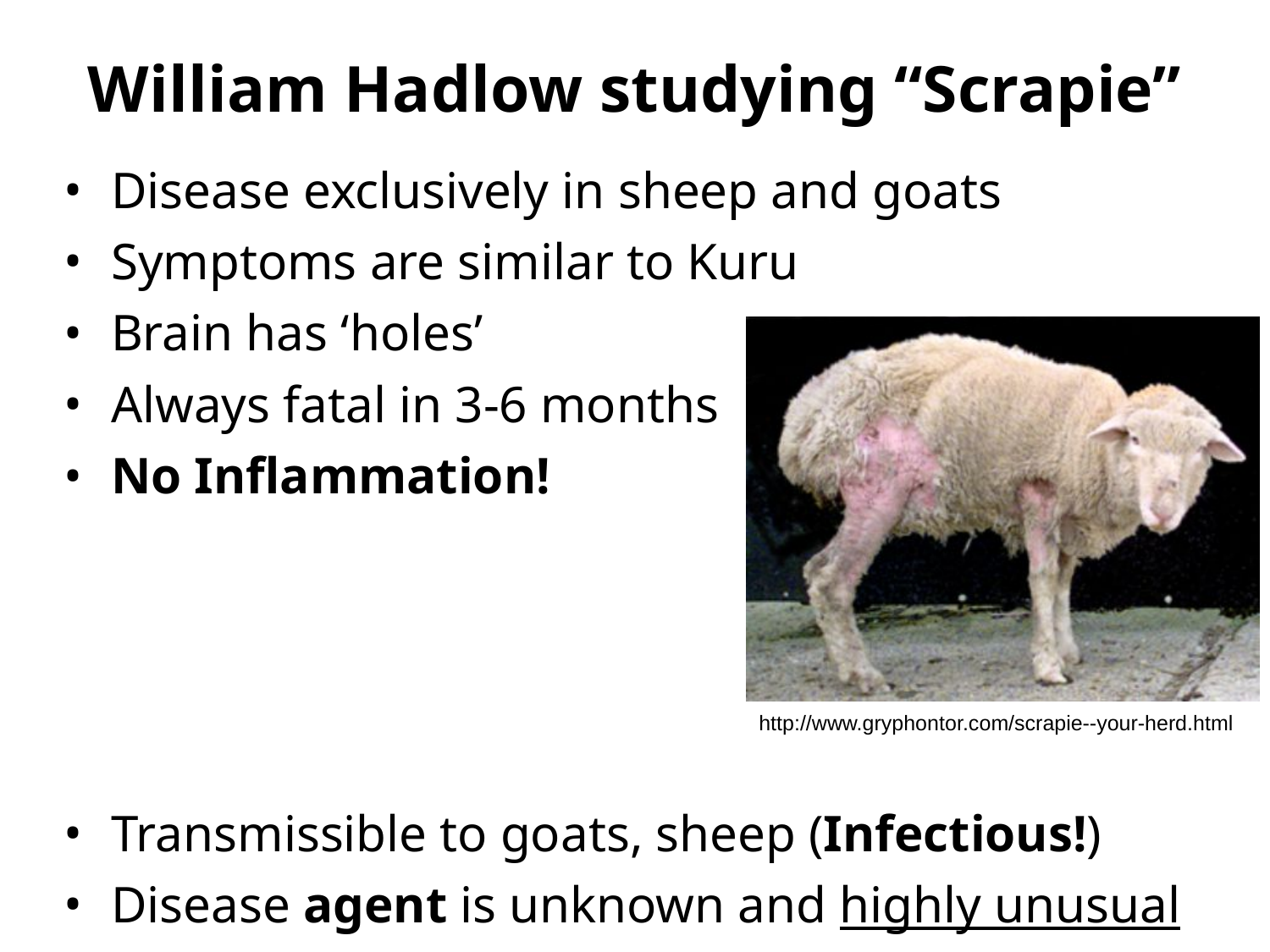

# William Hadlow studying “Scrapie”
Disease exclusively in sheep and goats
Symptoms are similar to Kuru
Brain has ‘holes’
Always fatal in 3-6 months
No Inflammation!
Transmissible to goats, sheep (Infectious!)
Disease agent is unknown and highly unusual
http://www.gryphontor.com/scrapie--your-herd.html

## Slide 27
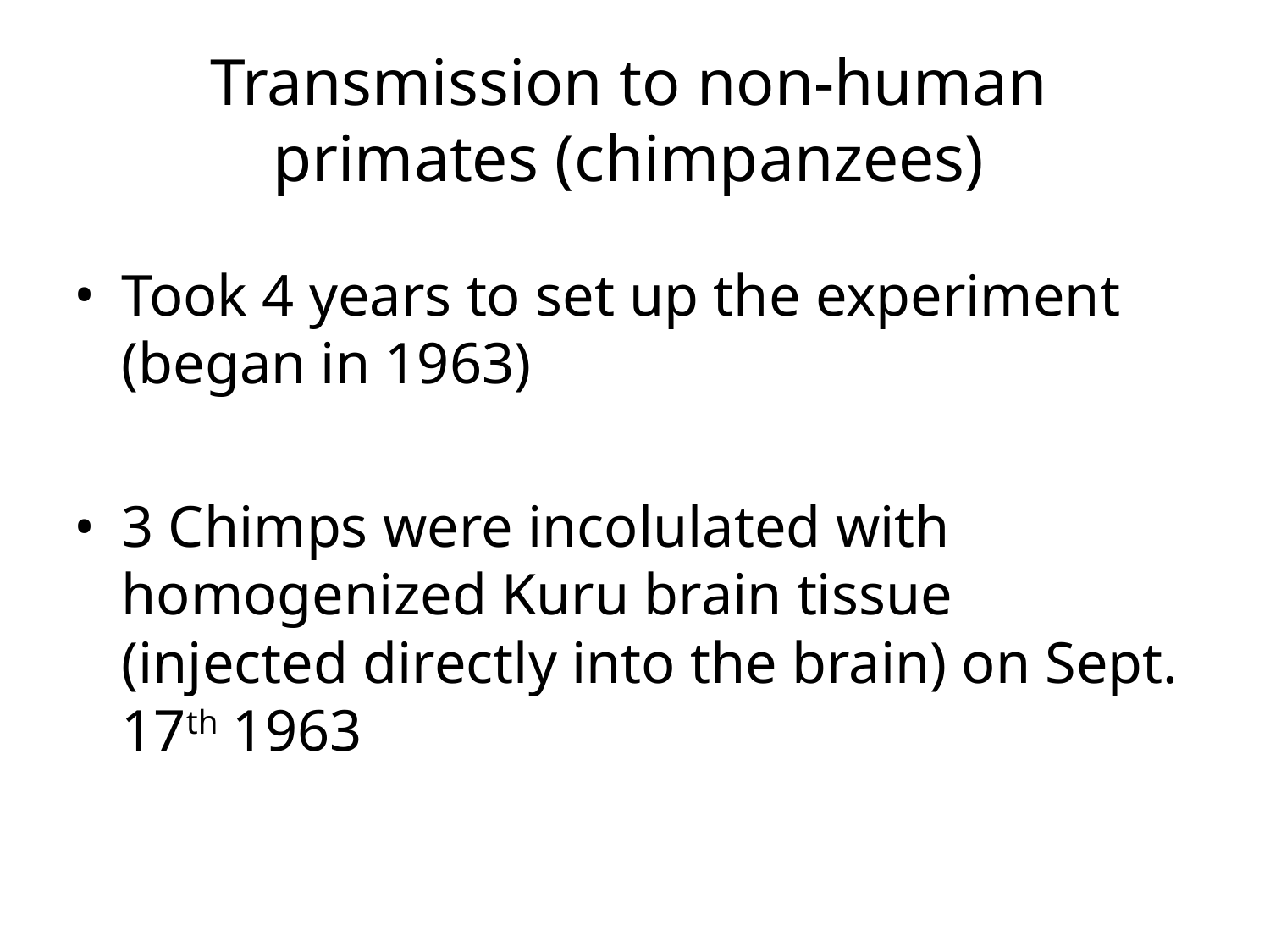

# Transmission to non-human primates (chimpanzees)
Took 4 years to set up the experiment (began in 1963)
3 Chimps were incolulated with homogenized Kuru brain tissue (injected directly into the brain) on Sept. 17th 1963

## Slide 28
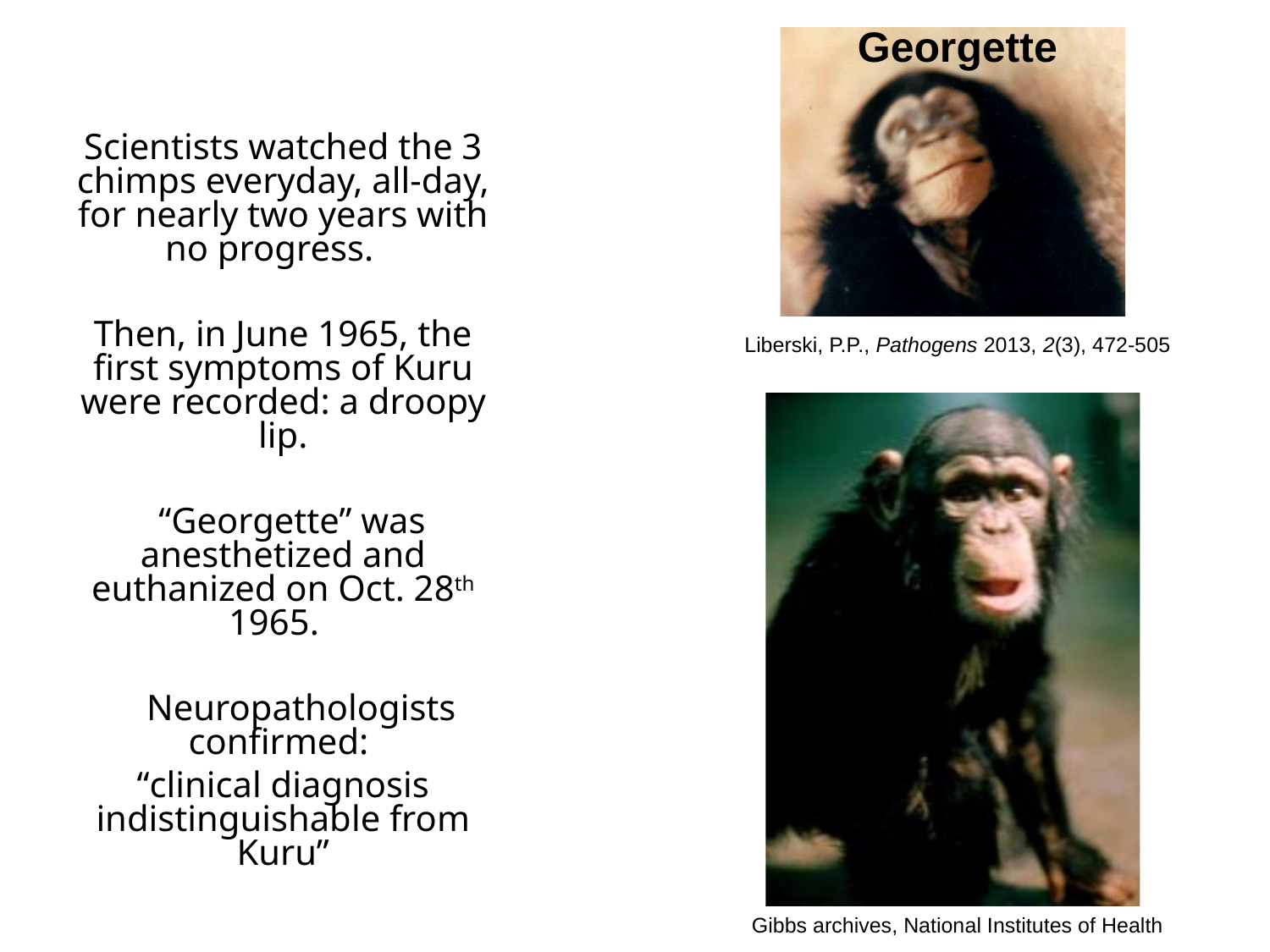

Georgette
# Scientists watched the 3 chimps everyday, all-day, for nearly two years with no progress.
Then, in June 1965, the first symptoms of Kuru were recorded: a droopy lip.
 “Georgette” was anesthetized and euthanized on Oct. 28th 1965.
 Neuropathologists confirmed:
“clinical diagnosis indistinguishable from Kuru”
Liberski, P.P., Pathogens 2013, 2(3), 472-505
Gibbs archives, National Institutes of Health

## Slide 29
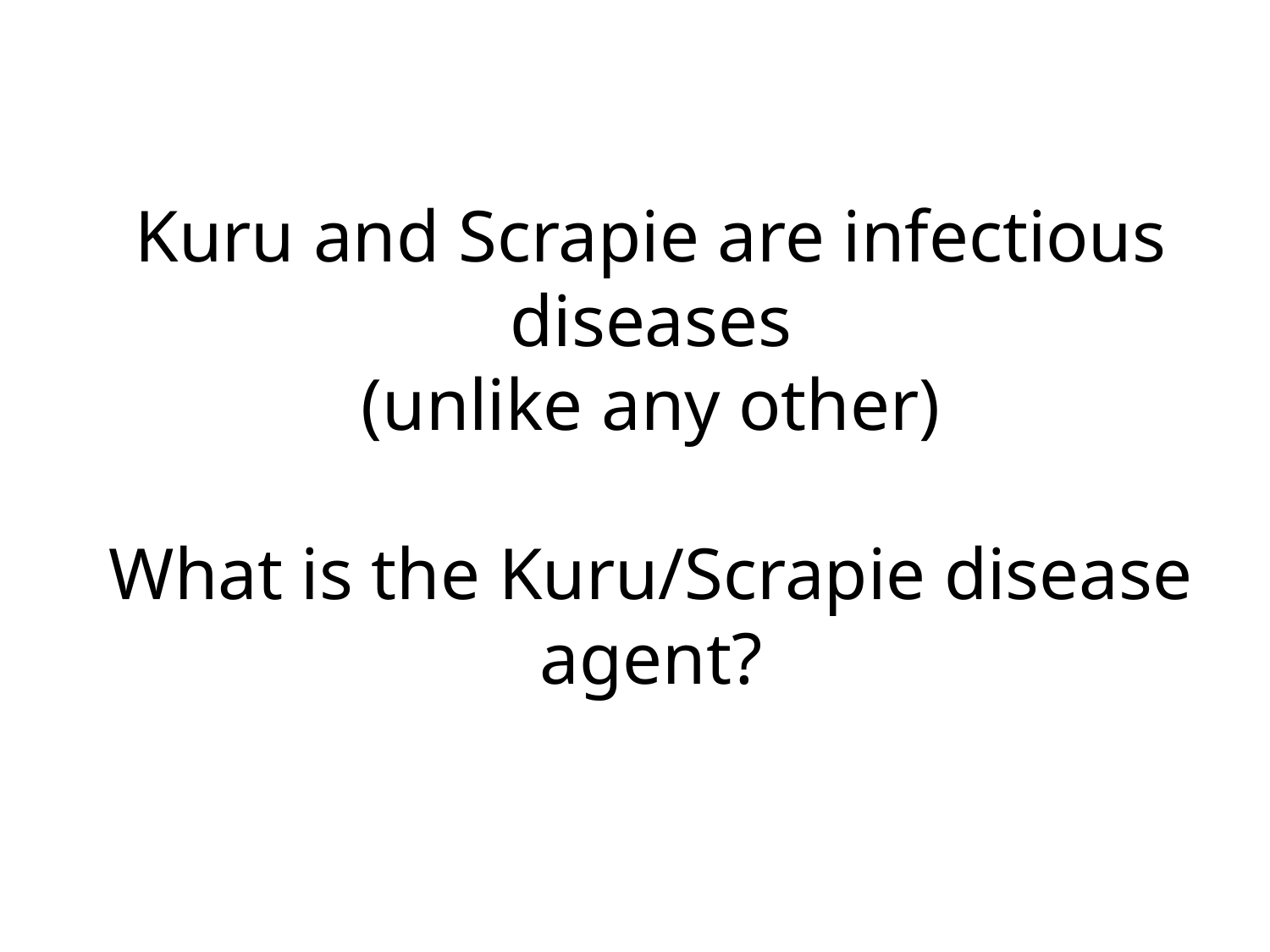

# Kuru and Scrapie are infectious diseases (unlike any other) What is the Kuru/Scrapie disease agent?
