# Supplementary material for: Cannibalism, Kuru, and Mad Cows: Prion Disease As a “Choose-Your-Own-Experiment” Case Study to Simulate Scientific Inquiry in Large Lectures
Source: PLoS Biol. 2016 Jan 20;14(1):e1002351. doi: 10.1371/journal.pbio.1002351 (PMC4720379; doi:10.1371/journal.pbio.1002351)
Supplement: S1 Text — To be read by the instructor only. (PDF) [file pbio.1002351.s003.pdf]

An Inexplicable Disease

In-class, prion disease case study

Justin K. Hines

Total Estimated Time: 75 minutes - Can be continuous or broken into two periods:

Period 1 (50 minutes):

10" Introduction and disease causes mini-lecture
15" Inquiry-driven activity: Student investigations
10" Inquiry-driven activity: Mock-conference
5" Class discussion of evidence
10" Revelations mini-lecture

Period 2 (25 minutes):

25" Prions mini-lecture

A bioethics module is available to extend the activity if desired by the instructor.
Email: hinesj@lafayette.edu

True Story: While the ways students acquire knowledge in this activity are largely fictionalized, the disease and most of the facts gathered by the students are real (the only exception is that results of the microbiologists' investigation reflect what real results may have looked like but have been fictionalized). The activity attempts to simulate the difficulties faced by Dr. Carleton Gajdusek and co-workers in their initial studies of the disease Kuru among the people of the Fore tribe, in Papua New Guinea from 1957-1959. This information is not shared with the students until later in the activity.

Purpose of *An Inexplicable Disease*: To engage students in the process of scientific inquiry through simulated investigation and directed collaboration. Students explore concepts like the importance of collaboration and communication between scientists, the difficulty and uncertainty of scientific investigations, and the often unsavory, but important utility of animal research. The inquiry-driven activity and mock-conference provide students opportunities to generate their own insights into how scientific knowledge is generated, tested, disseminated, and used. In addition, students are introduced to relevant biological concepts such as the hierarchy of disease causes and the role of protein misfolding in amyloid and prion diseases.

Description of the activity
(additional notes are included in the PowerPoint slides)

Introduction and disease causes mini-lecture—Students are broken into groups of approximately four people. Each group assumes the role of a particular type of scientific investigator (either physicians or anthropologists) which determines the additional information they begin the exercise with as well as the specific types of additional investigations that are available to them. The groups are charged with answering a basic question regarding the new disease: Is it infectious, genetically inherited, or environmentally caused? The instructor gives brief mini-lecture to clarify this question further before beginning the activity.

Inquiry-driven activity: Student investigations—Student groups discuss and evaluate the information they have. They are given a list of additional investigations, which each take a certain amount of time to conduct. Students must decide which investigation they wish to conduct, knowing that they are limited to only 24 months of "game-time" for investigations. The groups iteratively gather new results from the instructor, discuss the results, and arrive at another decision regarding further investigation, until they complete 24 months of investigations. The activity gradually reveals conflicting evidence regarding the nature of the disease. Additionally, depending on the type of investigator role the students take on, and the choices they make regarding experimentation, the students are likely to come to different and conflicting conclusions regarding the nature of the disease, as did the actual investigators.

Inquiry-driven activity: Mock conference—In a simulation of a real series of meetings among scientists in 1959, the students then share their results and conclusions with their peers in a mock "scientific conference". This creates a chance for the students to explore why, despite all studying the same disease, they have reached conflicting conclusions. Initially, this appears to be due a lack of any one group having all the information, however, after being given the opportunity to share their knowledge and collaborate, it becomes evident that the evidence in total is either incomplete or self-contradictory.

Class discussion of evidence—The instructor engages the students in a class discussion of the evidence that they have uncovered and asks for student opinions regarding its interpretation.

Revelations mini-lecture—The instructor explains the factual story behind the activity and the resolution of the this conundrum by the eventual demonstration that Kuru is an infectious disease by experimentation with chimpanzees. The instructor ends by explaining that, unbeknownst to the actual investigators, Kuru is in fact a transmissible spongiform encephalopathy or "prion" disease.

Prions mini-lecture—The remaining time (or following class period) consists of a mini-lecture by the instructor explaining additional experiments that led to the prion theory, and a description of the current state of knowledge regarding prions, which serves as excellent example of cutting-edge biology as many questions remain unanswered to this day.

List of documents comprising the teaching materials:

Rules and Guidelines Handout – To be given to students prior to the activity (1 per student)

Group Packets (Anthropologists or Physicians) – To be handed out at the beginning of the activity (1 per group; optimally equal number of groups with each type)

Each Packet contains:

Activity Introduction: "An Inexplicable Disease – Also displayed overhead and read aloud by the instructor to begin the inquiry-driven activity.

Four copies of Anthropologist or Physician handouts – One copy per student in group. This handout shows additional information on the front and options for further investigations on the reverse side.

Group Recorder sheet – One per group; used by the Recorder to organize and record group opinion and progress

Additional Investigation Sheets – To be kept by the instructor and handed out to groups during the "student investigation" phase of the activity. Sheets are given to the "Gopher" upon request during the inquiry-driven activity. Instructor should print out as many copies of each below as there are groups in the class.

There are six total sheets entitled:

Recruiting a microbiologist
Autopsies and recruiting a neuropathologist
Gaining the trust of the people
Recruiting a group of epidemiologists
Attempts to infect common laboratory animals
Attempts to infect chimpanzees

Part I and Part II PowerPoint presentations: Notes are included with each slide containing recommendations for presentation and additional information regarding the case story.

Optional assessment documents:

Pre-/Post- student opinion surveys – To be completed before and after the activity

Post-activity written survey – To be completed after the activity
