# Supplementary material for: Cannibalism, Kuru, and Mad Cows: Prion Disease As a “Choose-Your-Own-Experiment” Case Study to Simulate Scientific Inquiry in Large Lectures
Source: PLoS Biol. 2016 Jan 20;14(1):e1002351. doi: 10.1371/journal.pbio.1002351 (PMC4720379; doi:10.1371/journal.pbio.1002351)
Supplement: S2 Text — To be given to students prior to the activity (one per student). (PDF) [file pbio.1002351.s004.pdf]

Inexplicable Disease Activity **- Rules and Guidelines**

GROUPS:

For this activity, you will need to form groups of four people. Although all members of the group need to participate in the group discussions and decisions, each person also plays a specific role. The roles are:

Leader calls votes and keeps the group on task. All decisions need to be made democratically.
Recorder records group opinions and actions (directed by questions on the recorder sheet)
Task-master/gopher ensures the group stays focused and on task. Discussions should be on topic and no discussion with other (competing) groups. This person is also responsible for quickly getting new information from the instructor and distributing it to the group.
Reporter is responsible for reporting the group's results/opinions aloud to other groups and to the rest of the class when directed to do so by the instructor.

ABOUT THE ACTIVITY:

Each group will be given a disease to study and will take on the role of a specific type of investigator, *e.g*. physicians, anthropologists, etc. This will determine the type of information your group will initially possess and the type of additional research you are qualified to do.

Specifically, the **question** you are interested in answering is:

> Is this disease **infectious or non-infectious**, and if it is non-infectious, is it **genetically inherited or environmentally caused**?

We will discuss more about this question before we begin the activity.

HOW TO GET MORE INFORMATION:

Just like in a real scientific investigation, **gathering evidence takes time**. You will be given a sheet which has **options** for further investigations which will require a certain amount of "activity time" to complete. The goal of the investigation is to determine the cause of the disease as soon as possible and to present your conclusions to your fellow scientists (class-mates) at an in-class mock-"scientific conference". **You will have a limited amount of "activity time" to complete your research before the conference** and so you will need to make wise choices about what experiments to conduct. Once you decide (by majority vote!) what you want to do, send the Gopher up to the instructor and he or she will immediately receive the results of that investigation. The Recorder should make note of how much time passes and the group should discuss the new information. Please remember to be respectful to other groups and keep discussion volume to a minimum. The Recorder should then record your groups' opinions and you can move on to another investigation.

Generally, you can follow this **order of events**:

1. Discuss material with group
2. **Recorder** records individual and group opinions
3. **Leader** calls vote to determine next investigation
4. **Gopher** gets new results from instructor
5. Repeat steps 1-4 until 24 months have passed. **Reporter** should be ready to discuss the group's findings and opinions with the other groups and with the class.
