# Supplementary material for: Cannibalism, Kuru, and Mad Cows: Prion Disease As a “Choose-Your-Own-Experiment” Case Study to Simulate Scientific Inquiry in Large Lectures
Source: PLoS Biol. 2016 Jan 20;14(1):e1002351. doi: 10.1371/journal.pbio.1002351 (PMC4720379; doi:10.1371/journal.pbio.1002351)
Supplement: S4 Text — To be handed out at the beginning of the activity (one per group; optimally equal number of groups with each type). (PDF) [file pbio.1002351.s006.pdf]

An Inexplicable Disease

It is March, 1957. You travel to a remote island region to study a strange disease affecting the indigenous people. The people are hunters and farmers without written language or any modern technology. Despite being vicious warriors, they are extremely friendly and want your help. They believe they are being killed by sorcery. Healthy individuals will suddenly develop a strange walk, followed by slurred speech, facial ticks, and uncontrolled fits of laughter. You notice however, that while they lack the physical ability to control their speech, they remain intelligent and coherent. Within only three months, they will become completely incapacitated, unable to move or eat. In a cruel twist however, they remain conscious, and sadly, fully aware of their own suffering. Most die of starvation or dehydration due to the inability to swallow. Some face a possibly more merciful, early death from secondary infections such as pneumonia (due to inability to cough). Once the first symptoms occur, **death is certain**, with 100% dying in 3-6 months. Treating the symptoms and secondary infections only prolongs patient suffering… you decide you must discover the cause of the disease, and quickly, as one person in the tribe dies every three days and the illness appears to be spreading, especially among children. You plan to do more research and report your findings at an international scientific conference in March, 1959. You have two years to gather as much information as you can before then.

ANTHROPLOGISTS' HAND-OUT

ANTROPOLOGISTS' ADDITIONAL INFORMATION:

Your group is made up of well trained **anthropologists.**
Here are the **additional observations** you have made:

The disease is specific to a tribe of ~12,000 people that live in several distinct villages. The tribe is socially isolated from neighbors with little inter-tribe marriage.

Despite occasional violent and non-violent contact, neighboring tribes are not affected – nor have any medical workers become ill, thus the disease does not appear to be airborne.

Every village within the tribe is affected with an annual death rate of about 1% and increasing annually. Despite being in every village, the disease appears to affect specific families; some families are completely unaffected while others have multiple members who are sick.

The disease almost exclusively affects women and children. In most families where one woman is sick, other women of the same family and their children eventually develop the disease. This fact has strained relations between men and women in the tribe; the women believe the men are attacking the women and their children with sorcery. Men suspected of being sorcerers are being murdered by men who have lost their wives.

Your group has been establishing relationships with members of the tribe in one village, who allow you to better observe their social customs. At a feast, you notice that men always take the best parts of the pig for themselves, while women and children get what remains. When you ask a tribeswoman why, the woman responds angrily, saying that the men are greedy and selfish. However she also says that the women "get revenge on the men". When you ask her what she means by that, she immediately gets quiet and visibly uncomfortable with your question. There have always been rumors of cannibalism in this part of the world… possibly there is some connection, but she refuses to speak to you after this encounter.

MORE ON THE OTHER SIDE -->

POSSIBLE ADDITIONAL INVESTIGATIONS - ANTHROPOLOGISTS

Attempting to infect common laboratory animals

If you think the disease may be infectious, then you can quickly set up a small laboratory and keep some small mammals to do animal testing. Setting up the lab is quick, but it may take time for the animals to develop any symptoms. You feel you can be adequately set up to test mice, rats, guinea pigs and rabbits. **It will take six months to see any results**. Do you want to attempt to infect laboratory animals?

Gaining the trust of the people

If you would like to find out more about what the woman at the feast meant, then you need to spend more time gaining the people's trust before they will openly and honestly answer your questions. **It will take six months to do this**. Do you want to better get to know the tribespeople so that you can conduct more candid interviews?

Recruiting a group of epidemiologists

If you would like to know more about what the people of this tribe are eating and drinking relative to other neighboring tribes who are not sick, then you will need to recruit a group of epidemiologists to do an exhaustive study and allow them time to investigate. **This study will take one year.** Do you want to recruit a group of epidemiologists?

Recruiting a microbiologist

If you want to test food, water, and bodily fluid samples to try to identify and culture any infectious organisms which might be present, then you will need to recruit a **microbiologist** and allow him/her time to investigate. **This study will take six months**. Do you want to recruit a microbiologist?

ANTHROPLOGISTS' HAND-OUT

ANTROPOLOGISTS' ADDITIONAL INFORMATION:

Your group is made up of well trained **anthropologists.**
Here are the **additional observations** you have made:

The disease is specific to a tribe of ~12,000 people that live in several distinct villages. The tribe is socially isolated from neighbors with little inter-tribe marriage.

Despite occasional violent and non-violent contact, neighboring tribes are not affected – nor have any medical workers become ill, thus the disease does not appear to be airborne.

Every village within the tribe is affected with an annual death rate of about 1% and increasing annually. Despite being in every village, the disease appears to affect specific families; some families are completely unaffected while others have multiple members who are sick.

The disease almost exclusively affects women and children. In most families where one woman is sick, other women of the same family and their children eventually develop the disease. This fact has strained relations between men and women in the tribe; the women believe the men are attacking the women and their children with sorcery. Men suspected of being sorcerers are being murdered by men who have lost their wives.

Your group has been establishing relationships with members of the tribe in one village, who allow you to better observe their social customs. At a feast, you notice that men always take the best parts of the pig for themselves, while women and children get what remains. When you ask a tribeswoman why, the woman responds angrily, saying that the men are greedy and selfish. However she also says that the women "get revenge on the men". When you ask her what she means by that, she immediately gets quiet and visibly uncomfortable with your question. There have always been rumors of cannibalism in this part of the world… possibly there is some connection, but she refuses to speak to you after this encounter.

MORE ON THE OTHER SIDE -->

POSSIBLE ADDITIONAL INVESTIGATIONS - ANTHROPOLOGISTS

Attempting to infect common laboratory animals

If you think the disease may be infectious, then you can quickly set up a small laboratory and keep some small mammals to do animal testing. Setting up the lab is quick, but it may take time for the animals to develop any symptoms. You feel you can be adequately set up to test mice, rats, guinea pigs and rabbits. **It will take six months to see any results**. Do you want to attempt to infect laboratory animals?

Gaining the trust of the people

If you would like to find out more about what the woman at the feast meant, then you need to spend more time gaining the people's trust before they will openly and honestly answer your questions. **It will take six months to do this**. Do you want to better get to know the tribespeople so that you can conduct more candid interviews?

Recruiting a group of epidemiologists

If you would like to know more about what the people of this tribe are eating and drinking relative to other neighboring tribes who are not sick, then you will need to recruit a group of epidemiologists to do an exhaustive study and allow them time to investigate. **This study will take one year.** Do you want to recruit a group of epidemiologists?

Recruiting a microbiologist

If you want to test food, water, and bodily fluid samples to try to identify and culture any infectious organisms which might be present, then you will need to recruit a **microbiologist** and allow him/her time to investigate. **This study will take six months**. Do you want to recruit a microbiologist?

ANTHROPLOGISTS' HAND-OUT

ANTROPOLOGISTS' ADDITIONAL INFORMATION:

Your group is made up of well trained **<u>anthropologists</u>.**
Here are the **additional observations** you have made:

The disease is specific to a tribe of ~12,000 people that live in several distinct villages. The tribe is socially isolated from neighbors with little inter-tribe marriage.

Despite occasional violent and non-violent contact, neighboring tribes are not affected – nor have any medical workers become ill, thus the disease does not appear to be airborne.

Every village within the tribe is affected with an annual death rate of about 1% and increasing annually. Despite being in every village, the disease appears to affect specific families; some families are completely unaffected while others have multiple members who are sick.

The disease almost exclusively affects women and children. In most families where one woman is sick, other women of the same family and their children eventually develop the disease. This fact has strained relations between men and women in the tribe; the women believe the men are attacking the women and their children with sorcery. Men suspected of being sorcerers are being murdered by men who have lost their wives.

Your group has been establishing relationships with members of the tribe in one village, who allow you to better observe their social customs. At a feast, you notice that men always take the best parts of the pig for themselves, while women and children get what remains. When you ask a tribeswoman why, the woman responds angrily, saying that the men are greedy and selfish. However she also says that the women "get revenge on the men". When you ask her what she means by that, she immediately gets quiet and visibly uncomfortable with your question. There have always been rumors of cannibalism in this part of the world… possibly there is some connection, but she refuses to speak to you after this encounter.

MORE ON THE OTHER SIDE -->

POSSIBLE ADDITIONAL INVESTIGATIONS - ANTHROPOLOGISTS

Attempting to infect common laboratory animals

If you think the disease may be infectious, then you can quickly set up a small laboratory and keep some small mammals to do animal testing. Setting up the lab is quick, but it may take time for the animals to develop any symptoms. You feel you can be adequately set up to test mice, rats, guinea pigs and rabbits. **It will take six months to see any results**. Do you want to attempt to infect laboratory animals?

Gaining the trust of the people

If you would like to find out more about what the woman at the feast meant, then you need to spend more time gaining the people's trust before they will openly and honestly answer your questions. **It will take six months to do this**. Do you want to better get to know the tribespeople so that you can conduct more candid interviews?

Recruiting a group of epidemiologists

If you would like to know more about what the people of this tribe are eating and drinking relative to other neighboring tribes who are not sick, then you will need to recruit a group of epidemiologists to do an exhaustive study and allow them time to investigate. **This study will take one year.** Do you want to recruit a group of epidemiologists?

Recruiting a microbiologist

If you want to test food, water, and bodily fluid samples to try to identify and culture any infectious organisms which might be present, then you will need to recruit a **microbiologist** and allow him/her time to investigate. **This study will take six months**. Do you want to recruit a microbiologist?

ANTHROPLOGISTS' HAND-OUT

ANTROPOLOGISTS' ADDITIONAL INFORMATION:

Your group is made up of well trained **anthropologists.**
Here are the **additional observations** you have made:

The disease is specific to a tribe of ~12,000 people that live in several distinct villages. The tribe is socially isolated from neighbors with little inter-tribe marriage.

Despite occasional violent and non-violent contact, neighboring tribes are not affected – nor have any medical workers become ill, thus the disease does not appear to be airborne.

Every village within the tribe is affected with an annual death rate of about 1% and increasing annually. Despite being in every village, the disease appears to affect specific families; some families are completely unaffected while others have multiple members who are sick.

The disease almost exclusively affects women and children. In most families where one woman is sick, other women of the same family and their children eventually develop the disease. This fact has strained relations between men and women in the tribe; the women believe the men are attacking the women and their children with sorcery. Men suspected of being sorcerers are being murdered by men who have lost their wives.

Your group has been establishing relationships with members of the tribe in one village, who allow you to better observe their social customs. At a feast, you notice that men always take the best parts of the pig for themselves, while women and children get what remains. When you ask a tribeswoman why, the woman responds angrily, saying that the men are greedy and selfish. However she also says that the women "get revenge on the men". When you ask her what she means by that, she immediately gets quiet and visibly uncomfortable with your question. There have always been rumors of cannibalism in this part of the world… possibly there is some connection, but she refuses to speak to you after this encounter.

MORE ON THE OTHER SIDE -->

POSSIBLE ADDITIONAL INVESTIGATIONS - ANTHROPOLOGISTS

Attempting to infect common laboratory animals

If you think the disease may be infectious, then you can quickly set up a small laboratory and keep some small mammals to do animal testing. Setting up the lab is quick, but it may take time for the animals to develop any symptoms. You feel you can be adequately set up to test mice, rats, guinea pigs and rabbits. **It will take six months to see any results**. Do you want to attempt to infect laboratory animals?

Gaining the trust of the people

If you would like to find out more about what the woman at the feast meant, then you need to spend more time gaining the people's trust before they will openly and honestly answer your questions. **It will take six months to do this**. Do you want to better get to know the tribespeople so that you can conduct more candid interviews?

Recruiting a group of epidemiologists

If you would like to know more about what the people of this tribe are eating and drinking relative to other neighboring tribes who are not sick, then you will need to recruit a group of epidemiologists to do an exhaustive study and allow them time to investigate. **This study will take one year.** Do you want to recruit a group of epidemiologists?

Recruiting a microbiologist

If you want to test food, water, and bodily fluid samples to try to identify and culture any infectious organisms which might be present, then you will need to recruit a **microbiologist** and allow him/her time to investigate. **This study will take six months**. Do you want to recruit a microbiologist?

GROUP RECORDER SHEET **Group number and specialization: _______________________**

Group Member Names:

Group discussion - Initial impressions: After reading all of the information given to you, take a poll of your group members. Ask each member to give their current impressions about the disease; is it **genetically inherited, infectious, or environmentally caused**? Record the results here for each group member.

Additional investigation - What did your group decide to do first? _________________________

Group discussion –After discussing the new information**,** retake the previous poll and record the results a second time.

Additional investigation - What did your group decide to do next? _______________________

Group discussion- After discussing the new information, retake the previous poll and record the results a third time.

Additional investigation - What did your group decide to do next? _______________________

Group discussion - After discussing the new information, retake the previous poll and record the results.

More questions on other side →

Preparing for the conference: After 24 months have passed, the group should decide what the group "opinion" is regarding the disease. Please record this opinion below. What is the most important piece of evidence which lead you to that conclusion? Are there dissenters in the group? Why? What more would you like to know? The **Reporter** for the group should now be prepared to report your current opinion to the class at the scientific conference.

Group discussion – Final impressions: What does your group feel was the most important piece of information gained from the conference? Retake the previous poll and record the results. What would you still like to know? Again, the reporter should be prepared to report your group's current consensus to the class.
