# Supplementary material for: Cannibalism, Kuru, and Mad Cows: Prion Disease As a “Choose-Your-Own-Experiment” Case Study to Simulate Scientific Inquiry in Large Lectures
Source: PLoS Biol. 2016 Jan 20;14(1):e1002351. doi: 10.1371/journal.pbio.1002351 (PMC4720379; doi:10.1371/journal.pbio.1002351)
Supplement: S5 Text — To be kept by the instructor and handed out to groups during the “student investigation” phase of the activity. (PDF) [file pbio.1002351.s007.pdf]

RECRUITING A MICROBIOLOGIST

Six months pass. Soil, water, food, fecal, urine, blood and various tissue samples sent by your group back to the United States were analyzed by a group of microbiologists. No obvious disease agents were present in the blood samples and attempts to culture bacteria, fungi, and viruses from these samples failed. Other cultures readily grew micro-organisms but most were non-pathogenic. A table of the most common pathogen's they found is shown below:

Microbe	Disease	Endemic to area?	Healthy Samples	Affected (Sick) Samples
Salmonella typhi	Typhoid Fever	Yes	15	20
Plasmodium falciparum	Malaria	Yes	28	25
Hepatitis A virus	Hepatitis A	Yes	7	15

These diseases are all endemic in the area and none match the one you are studying. The microbiologists note however that when examining the blood samples, they found no difference in white blood cell counts between sick and healthy samples. They suggest that this would be quite odd for a deadly infectious disease.

RECRUITING A GROUP OF EPIDEMIOLOGISTS

One year passes. You recruit a group of epidemiologists to study the habits and environment of the tribe you are studying and compare these with their neighbors.

One neighboring tribe is directly across a large river from one of the diseased villages, but despite their close location, they have no incidence of the disease. The tribe you are studying inhabits a large, geographically diverse area, and despite this, all villages within the tribe are sick. The epidemiologists conclude this makes it very unlikely that the illness is due to a local environmental toxin.

To determine if the disease is caused by something in the diet, the epidemiologists perform an exhaustive study, cataloging all the food (plants, animals, sources of water) which have been eaten by the sick and healthy within the tribe, as well as those eaten by the healthy neighboring tribes. After one year, this investigation has turned up nothing out of the ordinary and nothing that correlates with the disease. One epidemiologist noted however that the tribespeople are hesitant and elusive when asked to list everything they have eaten.

GAINING THE TRUST OF THE PEOPLE

Six months pass. Your group becomes very close with several families in the tribe. When you press them about how women "take revenge" on men in the tribe, they eventually reveal to you that within the last two generations, food shortages forced them to experiment with cannibalism. They realized that they were throwing away a large source of food, and since that time, it had become a ritual in many families to cook and eat the recently deceased family members. It was considered an honor to feed your family in this way. Generally, the adult men were not allowed to take part in these rituals (women and children only) and when any man did participate, he typically only ate red meat, rather than delicacies like the brain. In this way, the women feel they can get back at the men, whom they also eat. People with leprosy or those who die from diarrhea were considered too unclean to eat, however people who die of sorcery are considered clean. The recent contact with "westerners" in the past decade, who act horrified when they learn of these rituals, has caused the tribe to become extraordinarily embarrassed and secretive about the practice. Everyone you speak with about this initially denies it ever having happened. Some eventually admit the practice happened in the past, but all say that it was abandoned a few years before you arrived.

PERFORMING EXTENSIVE AUTOPSIES AND RECRUITING A NEUROPATHOLOGIST

Six months pass. Several full autopsies are conducted and every part of the body is excised and examined. The brains are fixed in formalin solution and shipped to a neuropathologist in the United States. Examinations of the bodies of those affected with the disease reveal no specific organ damage (excluding the brain). As was suspected by physical examinations of the sick, no evidence of inflammation or immune system response can be found.

Below is the report from the neuropathologist:

First examination: Brains appear normal.
After 6 weeks to sufficiently solidify, various portions were thinly sliced and examined under a microscope. Extensive brain damage was localized specifically to the cerebellum (region of the brain controlling complex motor functions), while no damage was apparent in the cerebrum (consciousness and higher order thought). Brain damage in cerebellum was severe, with extensive deterioration of neurons. Pattern of degeneration is unlike any known neurodegenerative disease, but the damage is reminiscent of Alzheimer's disease. It looks as if the brain had aged however some samples are from children. No evidence of infection was detected… nor ANY evidence of inflammation or immune response whatsoever!!! Very strange… Clinical diagnosis is unknown but in my professional opinion, evidence points toward a non-infectious cause.

J. Doe M.D.

ATTEMPTS TO INFECT COMMON LABORATORY ANIMALS

Six months pass. You set up a small lab near one of the villages. Over the course of a year you attempt to infect mice, rats, guinea pigs and rabbits through feeding experiments, intravenous injections, and even direct inoculation of infected material into the animals' brains. You observe all the lab animals, including uninfected animals as controls. After six months, none of the animals have come down with any symptoms of the disease. You infected both adult and new-born animals. A few of the adult animals have died (and dissection has not revealed anything unusual) but some of the young animals may live another 1-5 years. It is still possible that they may develop the disease, but you will have to continue to observe them for years to come.

New option—your experience in doing animal work has opened up a new option for experimentation! See below:

NEW INVESTIGATION OPTION:

Attempting to infect non-human primates (chimpanzees).

If you really think the disease may be infectious, but perhaps only infectious to primates (like humans), then you can test this hypothesis, but it won't be easy. Since you obviously can't test human beings, you can decide to "go-for-broke" and try to infect chimpanzees. An experiment of this kind is extremely risky for you as it will require all the resources and time you have to set up the proper facility and acquire the animals. At least one year. In addition, you may not get any results for several additional years, meaning you may not have all the data for the conference. **This experiment will take a minimum of 3 years, but you will get the results available for however much time you have remaining.** Would you like to start this experiment with the time you have remaining?

ATTEMPTS TO INFECT CHIMPANZEES

DENIED

3 months pass. You spend 3 months writing a lengthy proposal to secure funding for such an ambitious endeavor. However, your request to set up a chimpanzee lab has been denied out-right by your funding agency. The funding agency cites your lack of sufficient evidence for the disease being infectious to justify both the extremely high cost of such an ambitious experiment, and the possible moral repercussions for doing this kind of experiment on non-human primates, particularly chimpanzees.
