# Supplementary material for: Cannibalism, Kuru, and Mad Cows: Prion Disease As a “Choose-Your-Own-Experiment” Case Study to Simulate Scientific Inquiry in Large Lectures
Source: PLoS Biol. 2016 Jan 20;14(1):e1002351. doi: 10.1371/journal.pbio.1002351 (PMC4720379; doi:10.1371/journal.pbio.1002351)
Supplement: S6 Text — To be completed after the activity at the instructor’s discretion. (PDF) [file pbio.1002351.s008.pdf]

Student Opinion Survey

Here are a number of statements that may or may not describe your beliefs about learning biology. You are asked to rate each statement by selecting a number between 1 and 5 where the numbers mean the following:

1. Strongly Disagree
2. Disagree
3. Neutral
4. Agree
5. Strongly Agree

Choose one of the above five choices that **best expresses your feeling** about the statement. If you don't understand a statement, leave it blank. If you have no strong opinion, choose 3.

We are asking that you express your own beliefs. **Your answers will <u>not</u> affect your grade.** Your instructors will never see your individual answers, only whether you participated and the class results as a whole. This information will be very helpful to us in an effort to design more effective science courses.

1. ______It is possible to explain biological ideas with everyday language.
2. ______I enjoy figuring out answers to biological questions.
3. ______Learning biology changes my ideas about how the natural world works.
4. ______It is a valuable use of my time to study the fundamental experiments behind biological ideas.
5. ______If I had plenty of time, I would take a biology class outside of my major requirements just for fun.
6. ______There is usually only one correct approach to solving a biology problem.
7. ______For me, biology is primarily about learning known facts as opposed to investigating the unknown.
8. ______We use this statement to discard the survey of people who are not reading the questions. Please select agree (not strongly agree) for this question to preserve your answers.
9. ______Most important scientific discoveries are the result of one very talented, intelligent, and/or lucky scientist working alone.
10. ______It is very important for scientists to go to meetings, like conferences, to communicate their results to other scientists.

Please <u>circle</u> your answer to the following questions or fill in the blank:

11. Current year in college: _________

12. What is your gender? __________

13. What is your race/ethnicity (select the one with which you most identify)?

Caucasian (White) Hispanic Native American African American

Asian Pacific Islander Other (please specify)

14. Currently, what is your level of interest in biology?

Very Low Low Moderate High Very High

15. What is your current major or most likely major: ________________________________
