# Supplementary material for: Cannibalism, Kuru, and Mad Cows: Prion Disease As a “Choose-Your-Own-Experiment” Case Study to Simulate Scientific Inquiry in Large Lectures
Source: PLoS Biol. 2016 Jan 20;14(1):e1002351. doi: 10.1371/journal.pbio.1002351 (PMC4720379; doi:10.1371/journal.pbio.1002351)
Supplement: S7 Text — To be completed before and after the activity at the instructor’s discretion. (PDF) [file pbio.1002351.s009.pdf]

Exit Assessment for *Inexplicable Disease* Activity **NAME: ______________________________**

Your feedback will help us understand which aspects of your experience in this activity are valuable to you and will help guide future changes to instruction. It is important that you know that **your responses will be anonymous**. Any data shared or published from this survey will be presented as aggregate numbers or anonymous comments and quotes. The instructor will, however, identify students who completed the survey, as part of the class assessment. The instructor will **not** obtain individual responses connected to student ID numbers. If you have any questions or concerns about the survey or confidentiality, please contact: ___________________

What please circle your **year in college**:

1st   2nd   3rd   4th   5th or more

What is your academic major? (if undecided, please indicate this and provide an area of interest)

Using the scale below, please rate your understanding of the following topics BEFORE and AFTER the *Inexplicable Disease* activity.

	No Understanding	Little Understanding	Some/ Moderate Understanding	Great Deal of Understanding
How new scientific knowledge is created and disseminated - BEFORE	☐	☐	☐	☐
How new scientific knowledge is created and disseminated - AFTER	☐	☐	☐	☐
How a scientist evaluates data - BEFORE	☐	☐	☐	☐
How a scientist evaluates data - AFTER	☐	☐	☐	☐
How scientists share and communicate information - BEFORE	☐	☐	☐	☐
How scientists share and communicate information - AFTER	☐	☐	☐	☐

Can you give any **specific examples** of how you feel that your understanding of scientists has changed?

Using the scale below, please indicate whether you agree or disagree with the following statements

	Strongly Disagree	Disagree	Agree	Strongly Agree
I enjoyed participating in this activity	☐	☐	☐	☐
This activity helped me learn	☐	☐	☐	☐
This activity was busy-work; it was not helpful	☐	☐	☐	☐
I did not enjoy participating in this activity	☐	☐	☐	☐
The activity gave me a better understanding of the process of scientific inquiry	☐	☐	☐	☐
I think this activity should be used in future offerings of this course	☐	☐	☐	☐
I think this activity should be used in other courses	☐	☐	☐	☐
This activity got me interested in learning about prions	☐	☐	☐	☐

Do you think that this activity helped you to improve any SKILLS? (Examples of relevant skills include but are not limited to: the ability to work in groups, evaluate data, form a hypothesis, or think critically)

Using the scale below, please rate your knowledge of the following topics BEFORE and AFTER the activity.

	No Knowledge	Little Knowledge	Some/ Moderate Knowledge	Great Deal of Knowledge
Prions - BEFORE	☐	☐	☐	☐
Prions - AFTER	☐	☐	☐	☐
Causes of human disease - BEFORE	☐	☐	☐	☐
Causes of human disease - AFTER	☐	☐	☐	☐
Human immune responses - BEFORE	☐	☐	☐	☐
Human immune responses - AFTER	☐	☐	☐	☐
The role of an epidemiologist - BEFORE	☐	☐	☐	☐
The role of an epidemiologist - AFTER	☐	☐	☐	☐
Issues surrounding animal research - BEFORE	☐	☐	☐	☐
Issues surrounding animal research - AFTER	☐	☐	☐	☐

Other than about prions, what else did you learn? Was there anything that you were surprised to have learned?

What specifically did you enjoy or not enjoy about the activity?

Considering all aspects of the activity, would you **recommend** that this activity be used in college courses? **Why or why not**?

Any other comments?
