# Supplementary material for: Cannibalism, Kuru, and Mad Cows: Prion Disease As a “Choose-Your-Own-Experiment” Case Study to Simulate Scientific Inquiry in Large Lectures
Source: PLoS Biol. 2016 Jan 20;14(1):e1002351. doi: 10.1371/journal.pbio.1002351 (PMC4720379; doi:10.1371/journal.pbio.1002351)
Supplement: S8 Text — Additional information regarding data collection, evaluation, and analysis. (PDF) [file pbio.1002351.s010.pdf]

## Supplemental Methods

***Post-activity written survey***

Student survey data were collected immediately following the administration of the activity and pooled from seven different offerings of five courses at three institutions (see **Table S1** below for course and institution descriptions). Because it is important to exclude responses from students who were unlikely to be reading the questions, we rejected surveys from students who failed to correctly answer two sets of inverted questions embedded on the survey (that is, a pair of questions that, for example, the first asks if they enjoyed the activity, followed by a question that asks if they did not enjoy the activity; absolute responses can be anything but should be inverted). Following exclusion of these surveys, a total 346 students responses to the survey were analyzed. Answers to some written questions were categorized and counted by theme. Themes were researcher generated following initial readings of all surveys. Two raters individually coded answers by theme and then any differences in coding were resolved by discussion. Answers to specific questions that were given in other, open response questions were included in counts. Summary statistics for some questions are given in **Fig 3**. A listing of various representative statements for the different themes is shown below:

***Theme: Importance of Communication and Collaboration in Science***

*I learned that it can be helpful to have different types of scientists work on a case*
*I know now that scientists do not do their work alone*
*I understand that scientists need to share their results*
*The ability for one's research to have a light-bulb go on in another scientist's head to make sense of something they previously didn't understand.*
*I was surprised to learn that something could be discovered in the scientific community and yet others in the community are unaware of it.*
*Scientists NEED other scientists*
*It seems so much more important for scientists doing specific research to look at explanations beyond their "niche" ex. The vets knew about Scrapie but the doctors didn't!*
*Before I thought that if a scientist made a great discovery that everyone would know about it. After this class, I've realized that this isn't the case. Scrapie was discovered >20 yrs. before Kuru.*

***Theme: Time Required to Do Science***

*I didn't realize it could take years to do some experiments and to answer some questions.*
*The experiments that scientists conduct take a lot of time and patience*
*I was surprised to see ground breaking discoveries in this particular field still to this day about a topic that originated so long ago.*

***Theme: Difficulty/Challenges of Being a Scientist***

*I can see more of the limitations that scientists have to deal with*
*Scientists are judgmental of themselves*
*Scientists aren't always right on their first try*
*I now realize how little support some scientists have*
*The struggle when one cannot do the experiment that they know will give them the answer*

*I've learned that scientists are always challenging other scientist's work*

*That scientists go through a variety of different experiments to come up with their final explanation which is normally challenged by another scientist*

***Theme: Ethical Issues (Animal Research)***

*I didn't realize all the reasons why there were limitations on chimp research*

*There is a moratorium on doing medical research with chimps. I was surprised but I understood why*

*I was surprised to learn about animal research and how people felt about working with chimps*

***Theme: Other Comments Relating to Scientific Inquiry***

*I understand better that scientists are also subject to biases, and can become married to their initial ideas.*

*The anthropology option affected how aware I am of science in the context of culture and time*

*It helped me to understand that it is critical to take up multiple viewpoints on a problem and not to get hung up on one hypothesis*

*I've learned that many scientists can be working on the same thing at once*

***Pre-post survey construction, validation, and administration***

In a second survey we also assessed changes in self-reported perceptions of biology as a result of the activity. Student survey data were collected in five independent sections of *Introduction to Biology* at Institution 1 (see **Table S1** for additional course and institution details) in the Fall 2012 and Fall 2013 semesters. Students were given a nine-question survey (**S7 Text**) immediately before and following the activity. Questions 1-7 were selected for relevance from the CLASS-Bio survey (reference 8, main text), while questions 8 and 9 were researcher-generated and added to address issues that are specifically relevant to the activity but not present in CLASS-Bio. The seven statements selected from the CLASS-Bio survey span five of the seven different categories from the survey: *Real World Connection, Enjoyment (Personal Interest), Problem Solving: Reasoning, Problem Solving: Synthesis and Application, and Conceptual Connections/Memorization*. One statement (#1 in our survey) was reworded from the original statement in CLASS-Bio for use in our survey.

The CLASS-Bio survey rates student perceptions of biology by scoring students' ability to give "expert-like" answers, as compared to practitioners (Biology PhDs). Expert responses are affirmative for questions 1-5 and 9 and negative for questions 6-8. Surveys were administered without explanation of purpose and post-surveys were independently administered without any reference to the pre-survey and subsequently paired pre- to post-. The survey was tested for expert response validity by surveying 17 biology practitioners (Biology PhDs). Questions without at least 90% agreement were discarded; one of the three researcher-generated statements was discarded due to lack of expert response validity. Student response validity was not independently tested for this survey by interviews, as it was for the CLASS-Bio survey, however survey reliability was extensively examined (see below).

\

***Pre-post survey data analysis and reliability***

Again, because it is important to exclude responses from students who were unlikely to be reading the questions, surveys we rejected from students who provided the same Likert response to all questions, or who incorrectly responded to a control question embedded on the survey ("We use this statement to discard the survey of people who are not reading the questions. Please select agree (not strongly agree) for this question to preserve your answers"). Only students who fulfilled all the survey requirements for both the pre- and post-surveys were included for analysis. Data from all five sections across both years were pooled prior to analysis (n=195 pairs). Shifts in student responses, recorded using a Likert scale, were analyzed using a Wilcoxon Signed-rank Test. To counteract the problem of multiple comparisons while maintaining a family-wise error rate of 0.05, significance was determined by using the Bonferroni correction (n=9). Individual test results with p-values less than 0.005 are considered significant and are indicated in **Fig 4**.

Reliability of our survey was evaluated in several ways. First, as was done for the original CLASS-Bio survey, we calculated as a test-retest coefficient of stability on student responses from two equivalent populations: two annual offering of *Introductory Biology* at Institution 1. This metric measures stability over time. Coefficients of stability for the pre-test scores between 2012 & 2013 cohorts were $r = 0.98$ for percent favorable, $r = 0.84$ for percent neutral, and $r = 0.94$ for percent unfavorable. Because $r = 0.80$ is considered an indication of high reliability, these numbers imply a high degree of reliability from year to year in our survey.

We also evaluated internal reliability separately by calculating McDonald's omega (a metric that accounts for multiple factors in the survey where, like Cronbach's alpha, values greater than 0.7 indicate significant internal reliability). McDonald's omega total was 0.84 with five factors allowed when calculated for either the pre-test or post-test survey results, indicating a high degree of internal reliability in the survey.

Finally, and also like the original CLASS-Bio survey, we asked whether our survey is also able to distinguish between declared biology majors and non-majors, a distinction that would be expected to be greatest in pre-test scores. To test this, we looked at the mean number of expert-like answers on the pre-test (out of nine questions in the survey) and compared the two groups. The mean pre-test scores were for biology majors and non-majors were 7.37 and 6.49, respectively ($p = 0.00377$, standard two-population t-test for means), indicating that our survey does distinguish these groups as expected. Also as expected, this difference between groups narrows significantly on the post-test scores and the difference between groups is no longer significant: mean scores were 7.92 and 7.46, respectively ($p = 0.0543$), for majors and non-majors on the post-test. These data also indicate that non-majors had greater gains on the survey, which is also a reasonable expectation for a survey that measures expert-like perceptions of biology. Indeed, the average gains (the number of additional questions answered like experts on the post-test vs. pre-test) for non-majors was 0.967 vs. 0.551 for majors. Gains for both groups were statistically significant using a standard paired t-test to test if the gain differs from 0 ($p = 0.00284$ for majors and $p < 0.0001$ for non-majors). This distinction in gains is also likely due to significant ceiling effects in the student cohort examined that differed between the two groups. For example, biology majors were nearly twice as likely to give the expert response to all 9 questions in on the pre-test as non-majors (27% vs. 15%, respectively).

**Table S1 – Characteristics of courses and institutions where data was collected**

| Institution | Institution Type | Carnegie Classification | Undergraduate Enrollment | Geographic Region |
|---|---|---|---|---|
| 1 | Private not-for-profit | Bac/A&S | ~2500 | Mid-Atlantic |
| 2 | Public | RU/VH | ~30,000 | Midwest |
| 3 | Public | RU/VH | ~35,000 | Mountain |

| Institution | Course Type | Course Size |
|---|---|---|
| 1 | Introductory Biology | ~100 |
| 1 | Advanced Biochemistry | ~12 |
| 2 | 1st Year Genetics Seminar | ~40 |
| 2 | Non-majors Microbiology | ~50 |
| 3 | Introductory Biology | ~90 |
