# Supplementary material for: Correction: Cannibalism, Kuru, and Mad Cows: Prion Disease As a "Choose-Your-Own-Experiment" Case Study to Simulate Scientific Inquiry in Large Lectures
Source: PLoS Biol. 2016 Mar 18;14(3):e1002425. doi: 10.1371/journal.pbio.1002425 (PMC4798775; doi:10.1371/journal.pbio.1002425)

## Slide 1
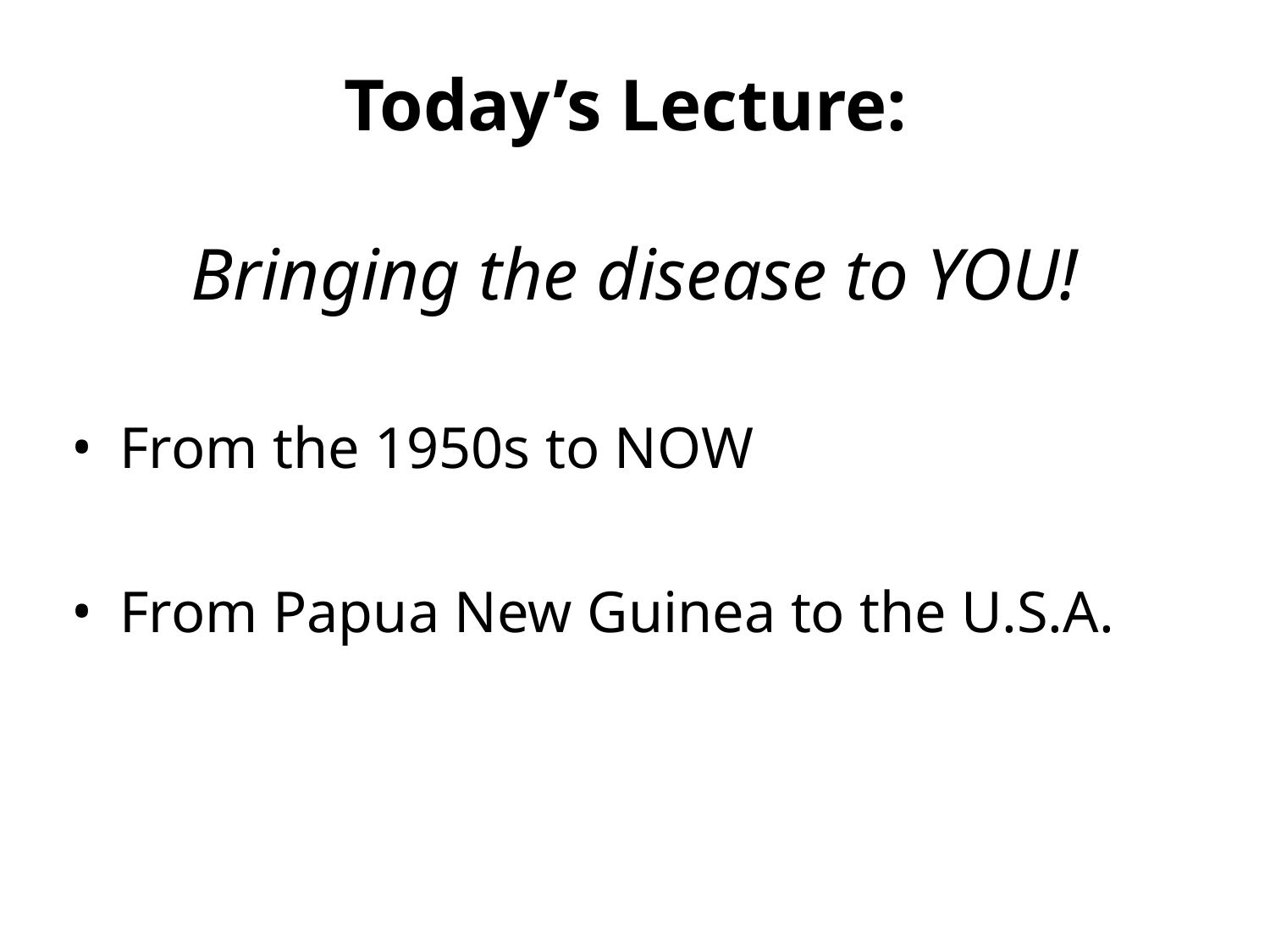

# Today’s Lecture: Bringing the disease to YOU!
From the 1950s to NOW
From Papua New Guinea to the U.S.A.

## Slide 2
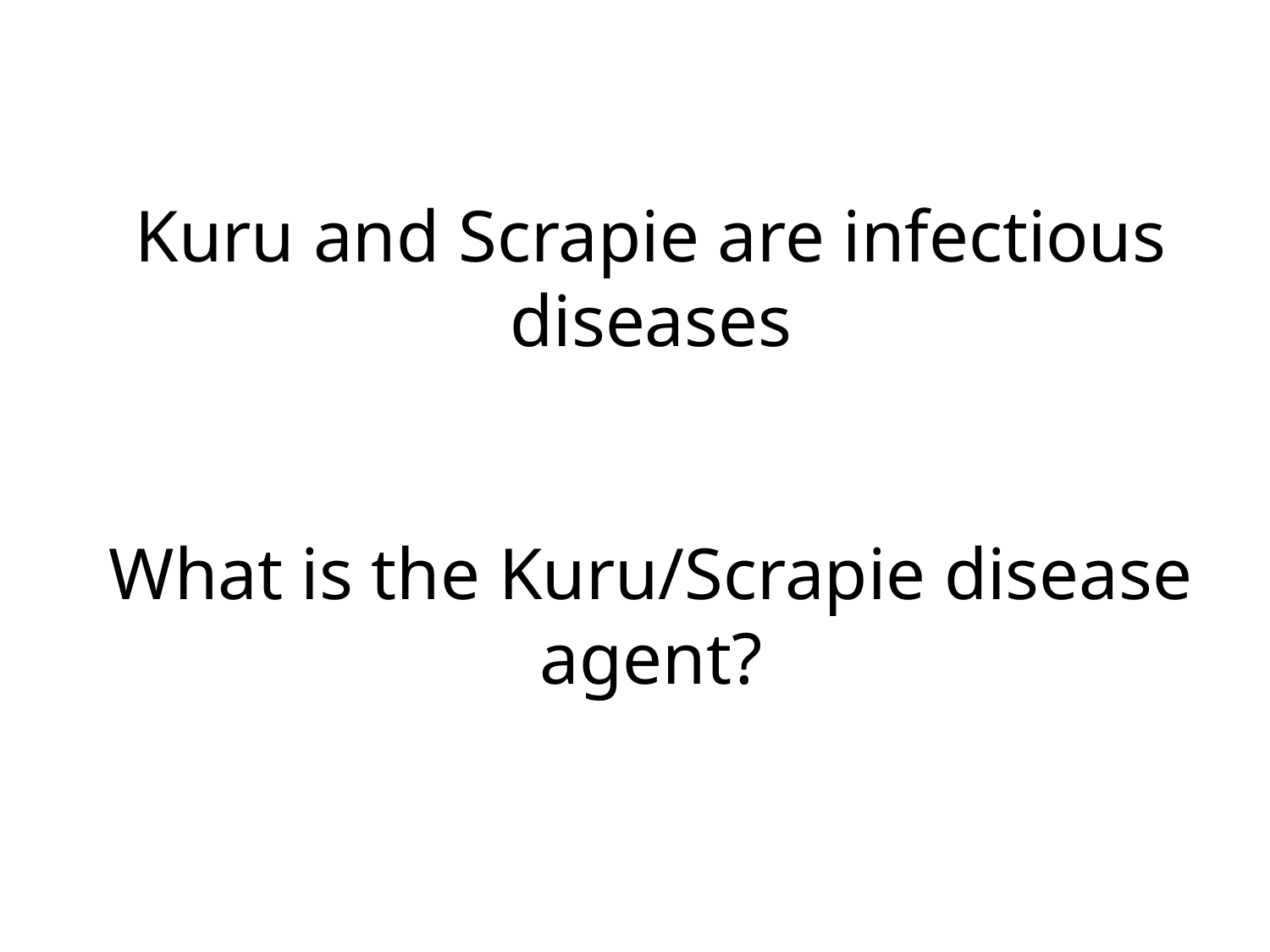

# Kuru and Scrapie are infectious diseases What is the Kuru/Scrapie disease agent?

## Slide 3
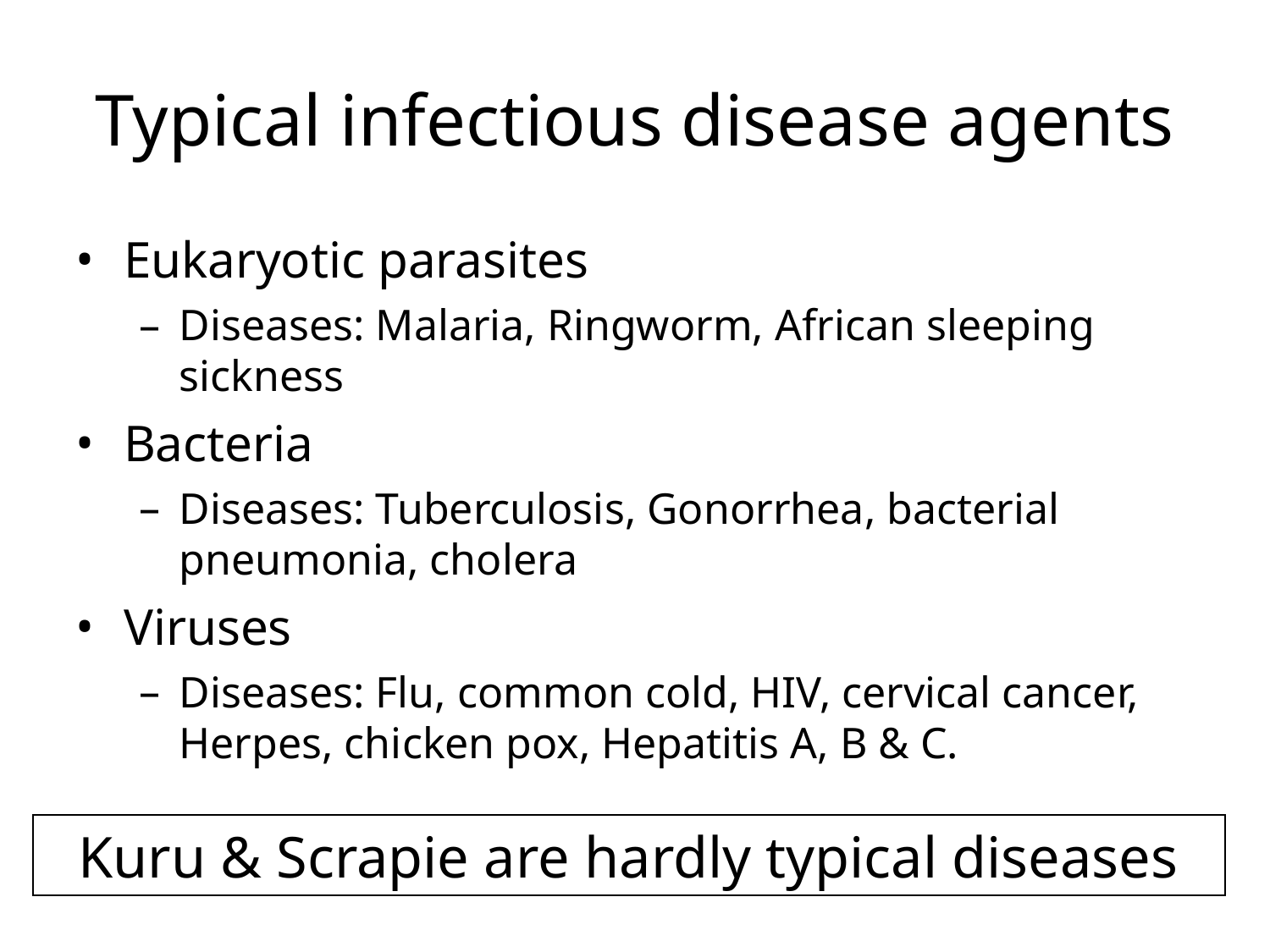

# Typical infectious disease agents
Eukaryotic parasites
Diseases: Malaria, Ringworm, African sleeping sickness
Bacteria
Diseases: Tuberculosis, Gonorrhea, bacterial pneumonia, cholera
Viruses
Diseases: Flu, common cold, HIV, cervical cancer, Herpes, chicken pox, Hepatitis A, B & C.
Kuru & Scrapie are hardly typical diseases

## Slide 4
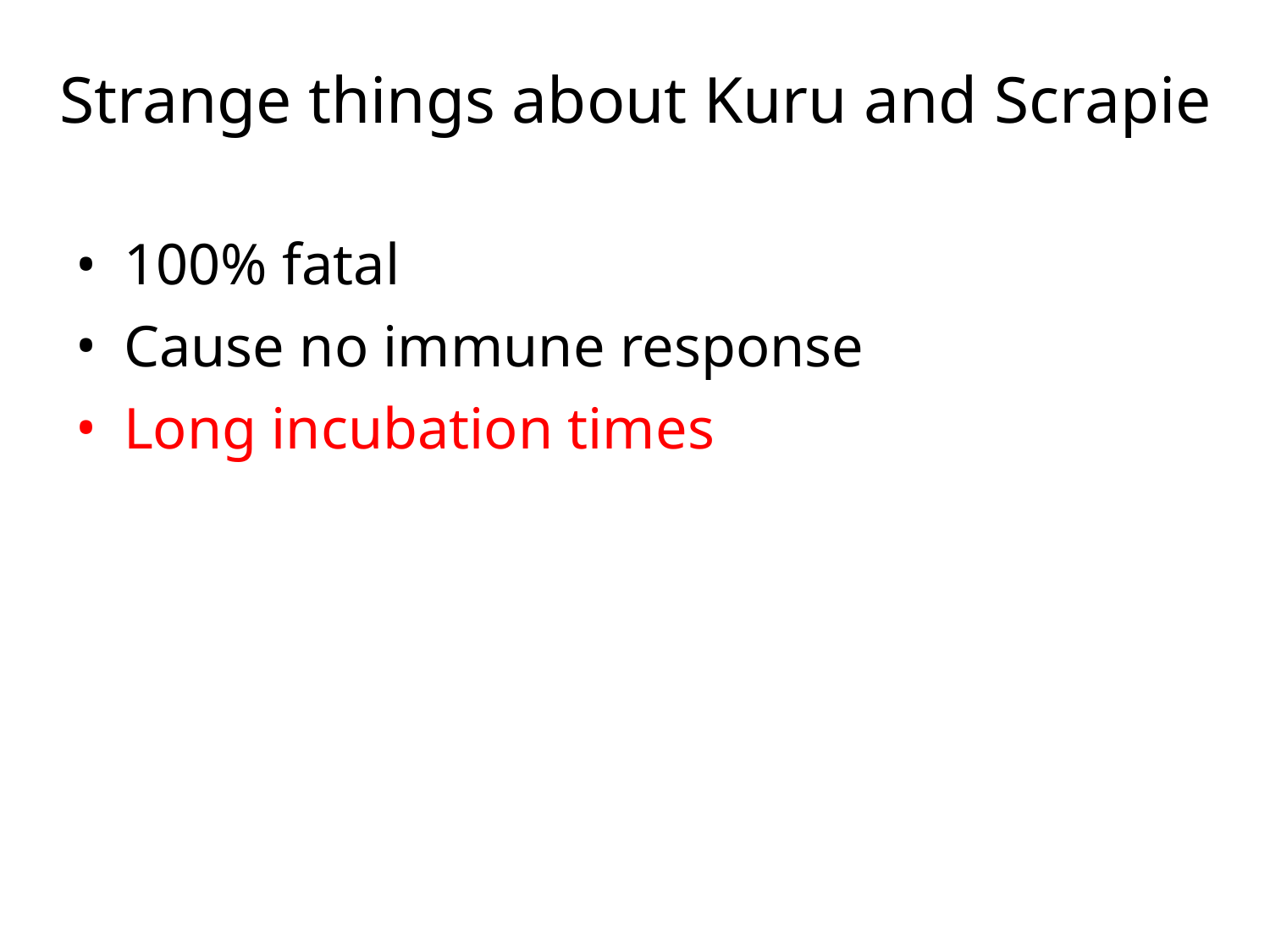

# Strange things about Kuru and Scrapie
100% fatal
Cause no immune response
Long incubation times

## Slide 5
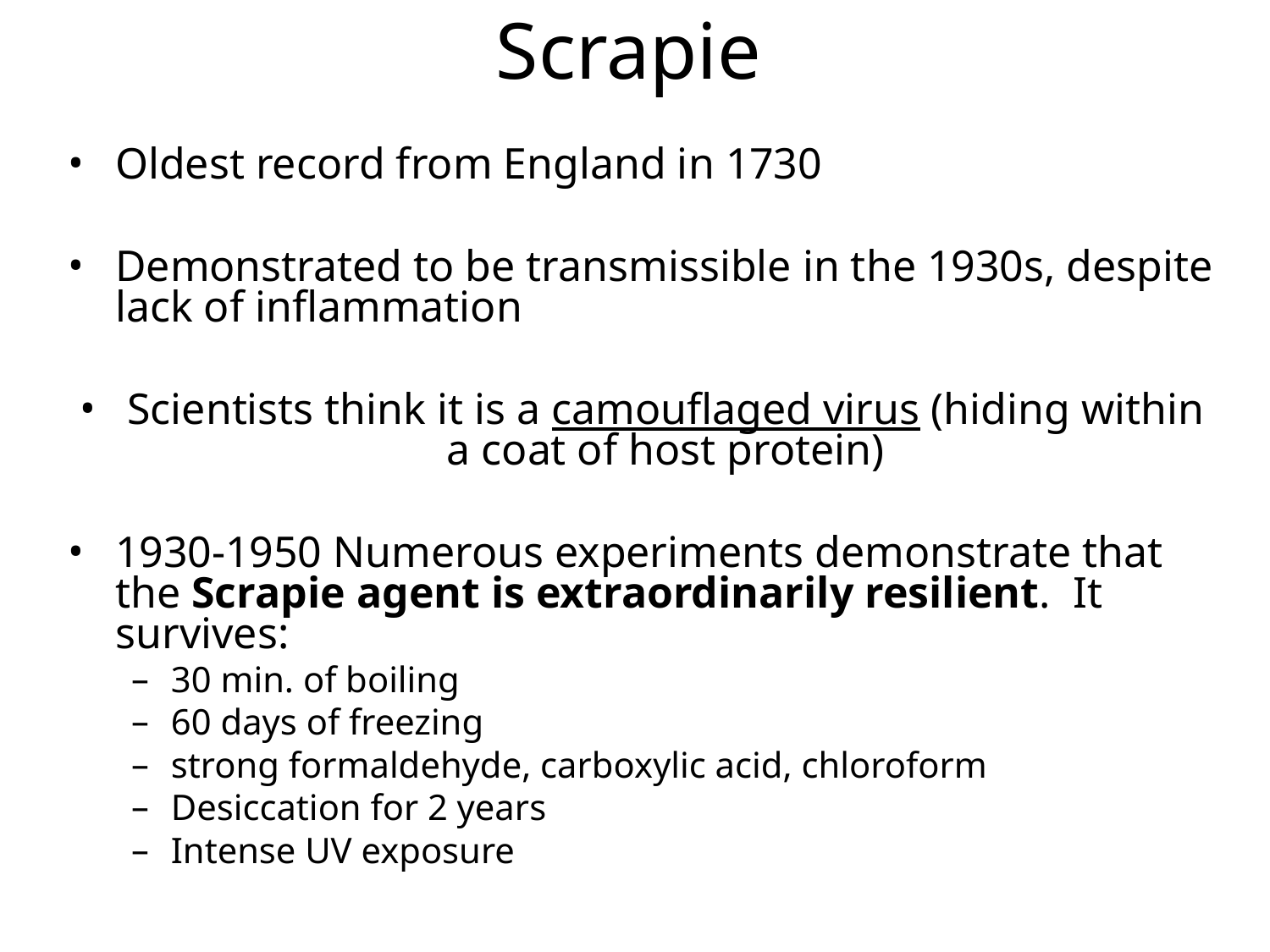

# Scrapie
Oldest record from England in 1730
Demonstrated to be transmissible in the 1930s, despite lack of inflammation
Scientists think it is a camouflaged virus (hiding within a coat of host protein)
1930-1950 Numerous experiments demonstrate that the Scrapie agent is extraordinarily resilient. It survives:
30 min. of boiling
60 days of freezing
strong formaldehyde, carboxylic acid, chloroform
Desiccation for 2 years
Intense UV exposure

## Slide 6
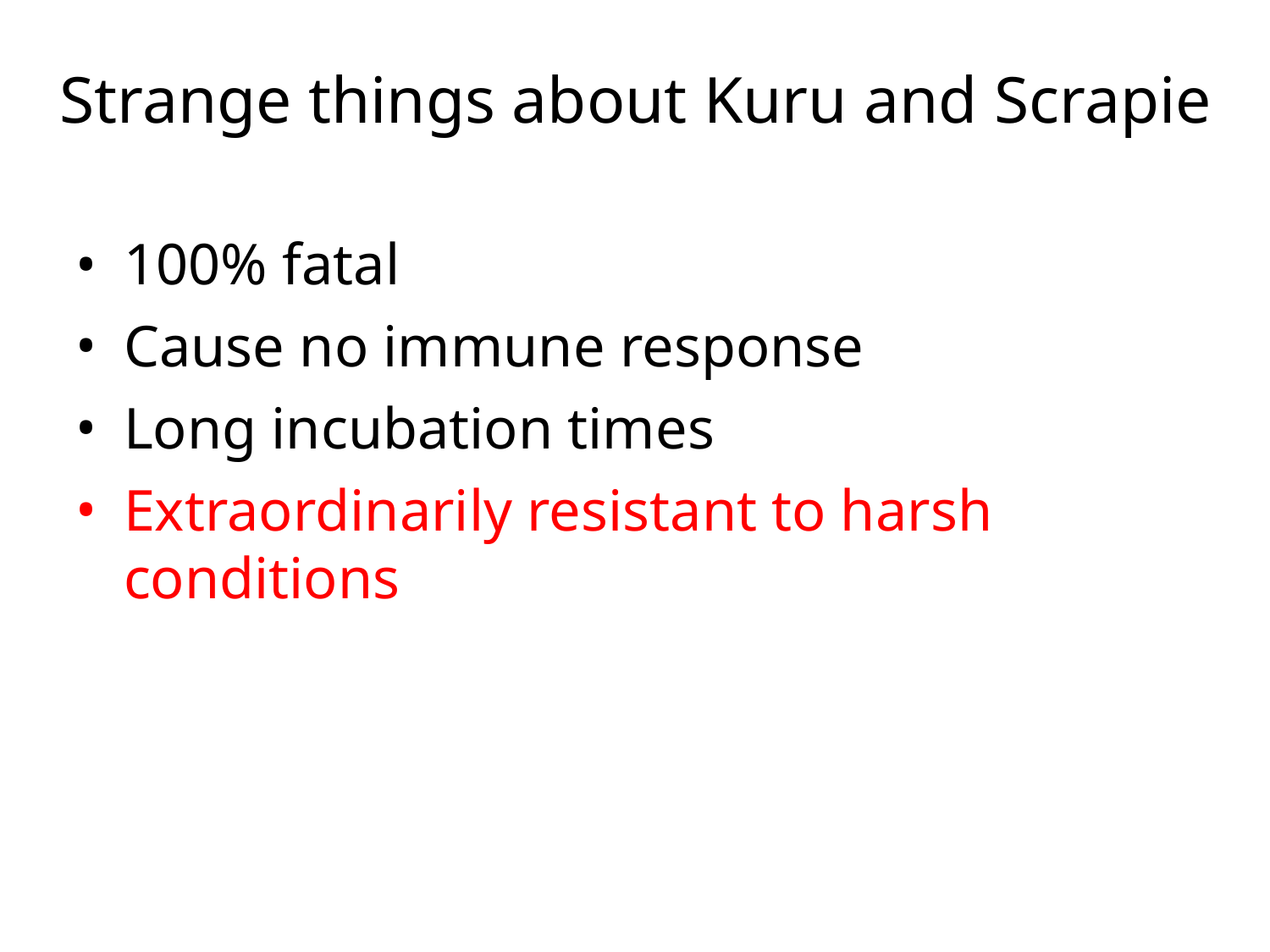

# Strange things about Kuru and Scrapie
100% fatal
Cause no immune response
Long incubation times
Extraordinarily resistant to harsh conditions

## Slide 7
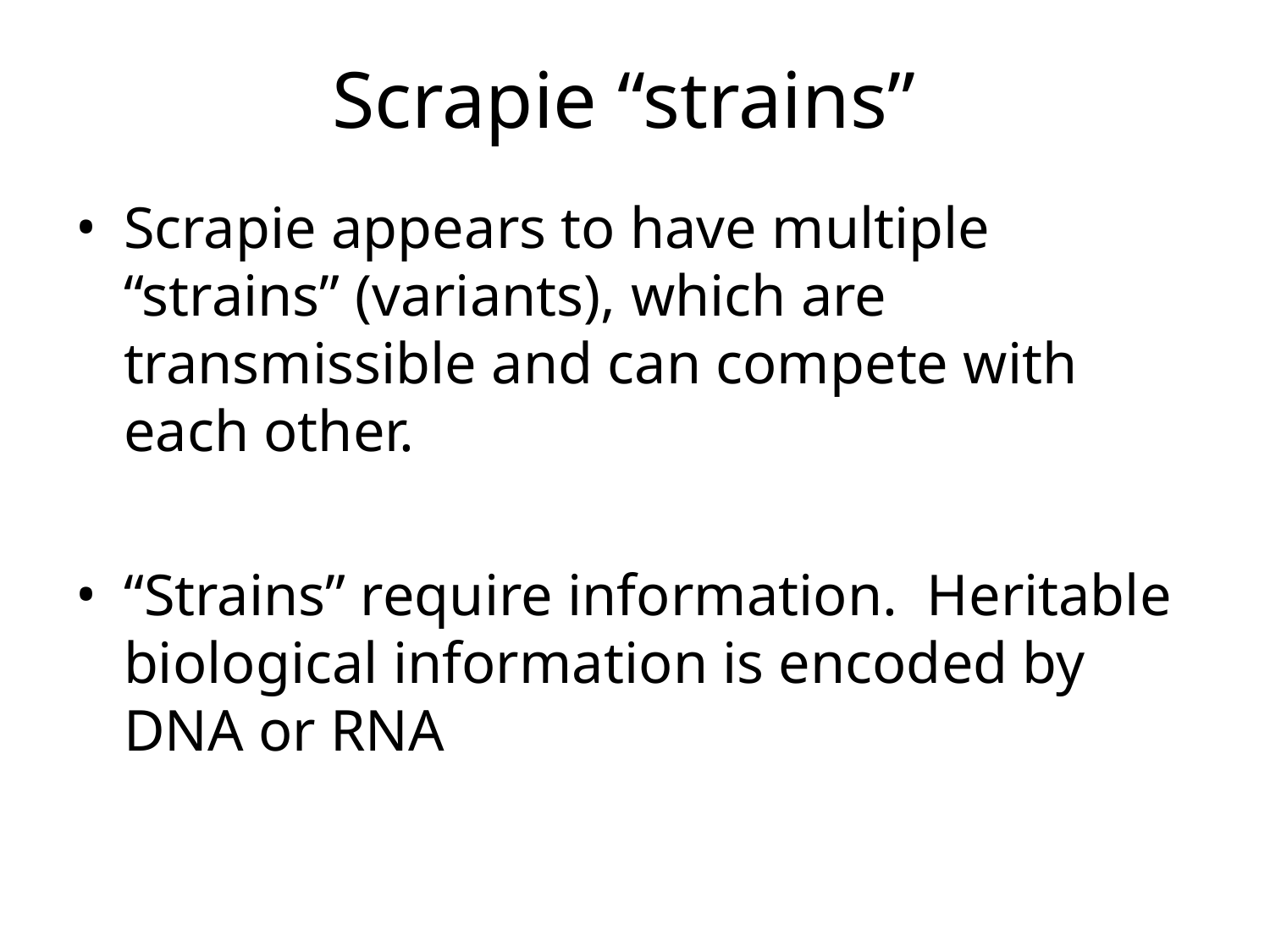

# Scrapie “strains”
Scrapie appears to have multiple “strains” (variants), which are transmissible and can compete with each other.
“Strains” require information. Heritable biological information is encoded by DNA or RNA

## Slide 8
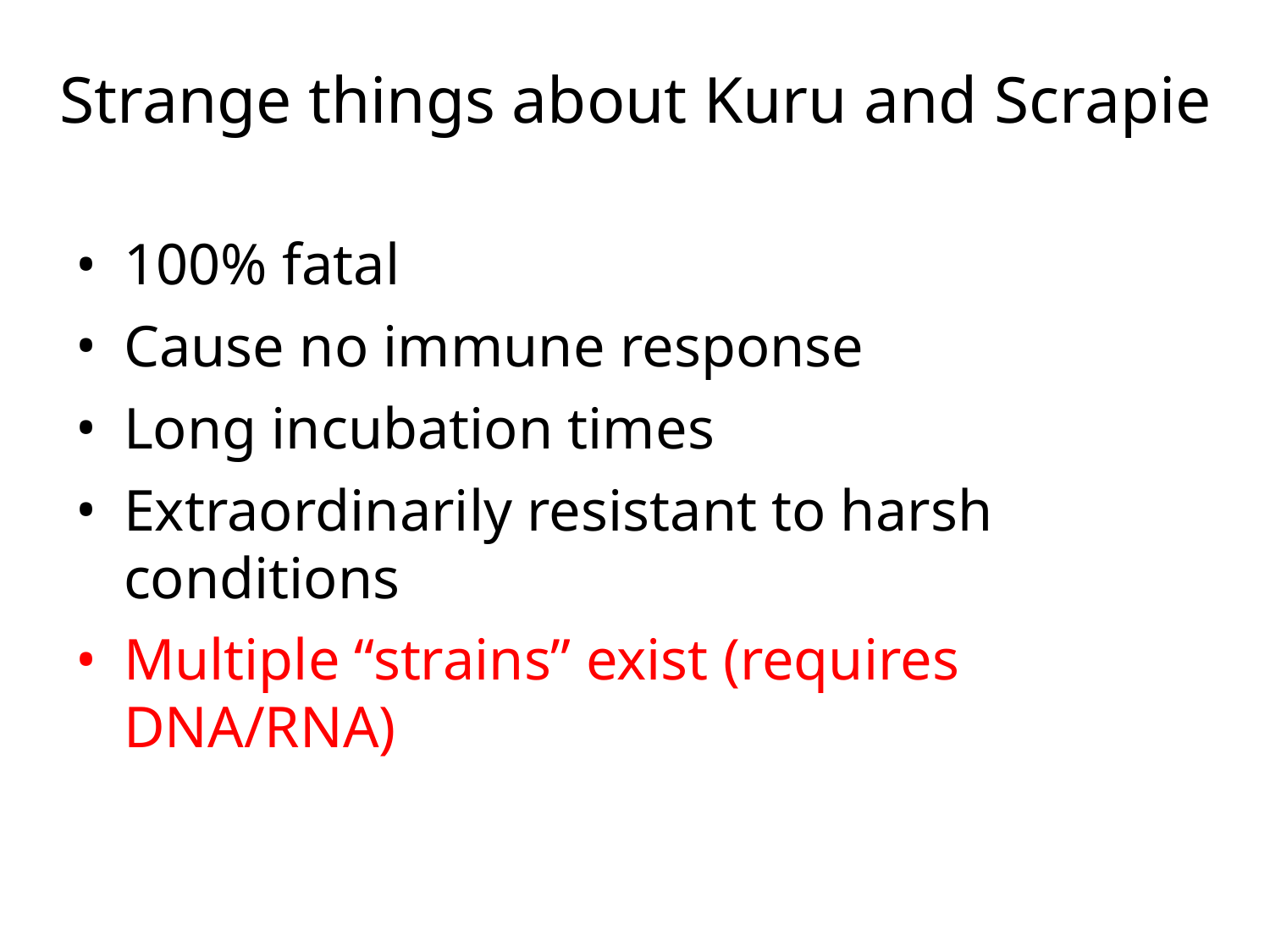

# Strange things about Kuru and Scrapie
100% fatal
Cause no immune response
Long incubation times
Extraordinarily resistant to harsh conditions
Multiple “strains” exist (requires DNA/RNA)

## Slide 9
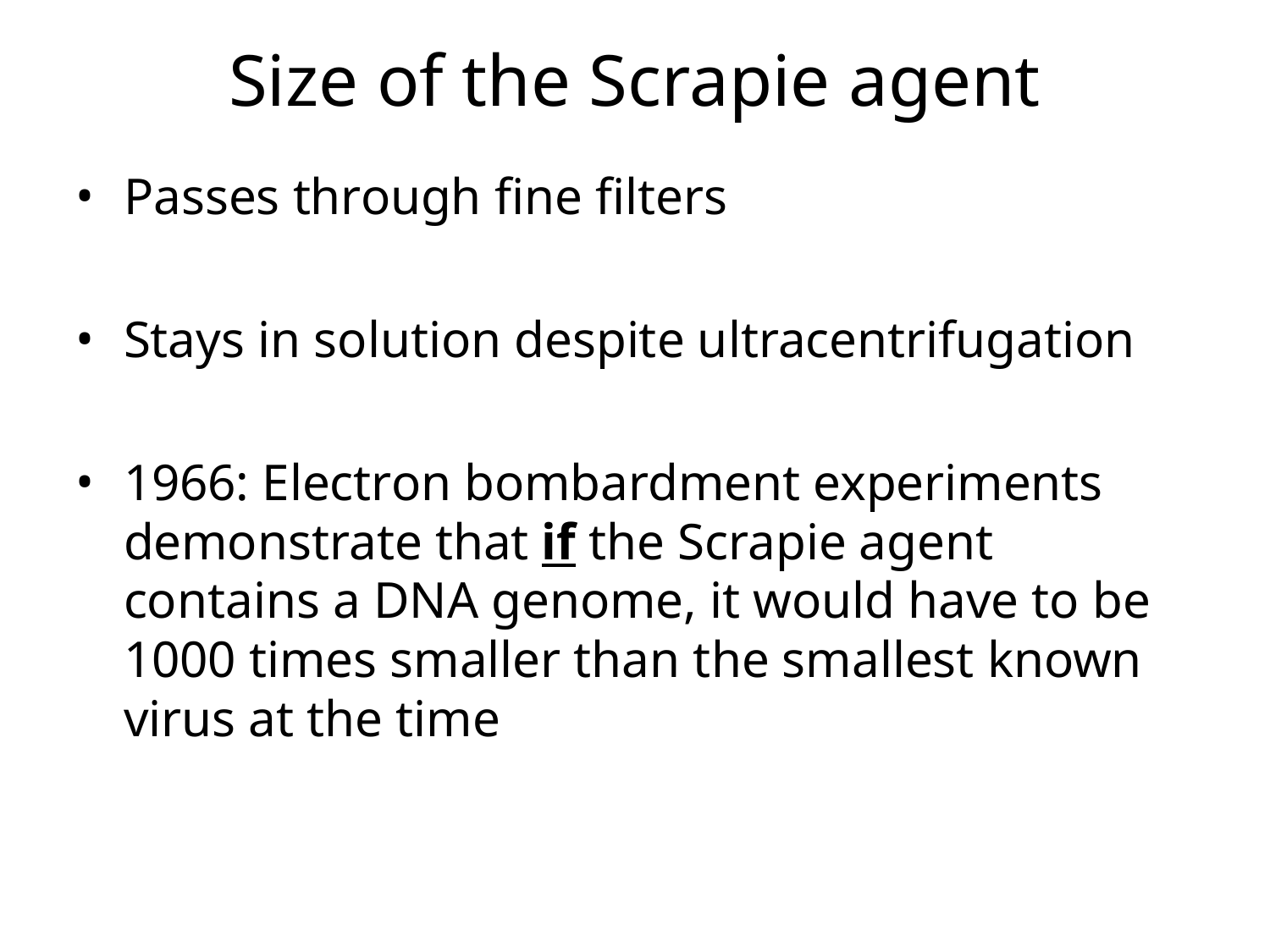

# Size of the Scrapie agent
Passes through fine filters
Stays in solution despite ultracentrifugation
1966: Electron bombardment experiments demonstrate that if the Scrapie agent contains a DNA genome, it would have to be 1000 times smaller than the smallest known virus at the time

## Slide 10
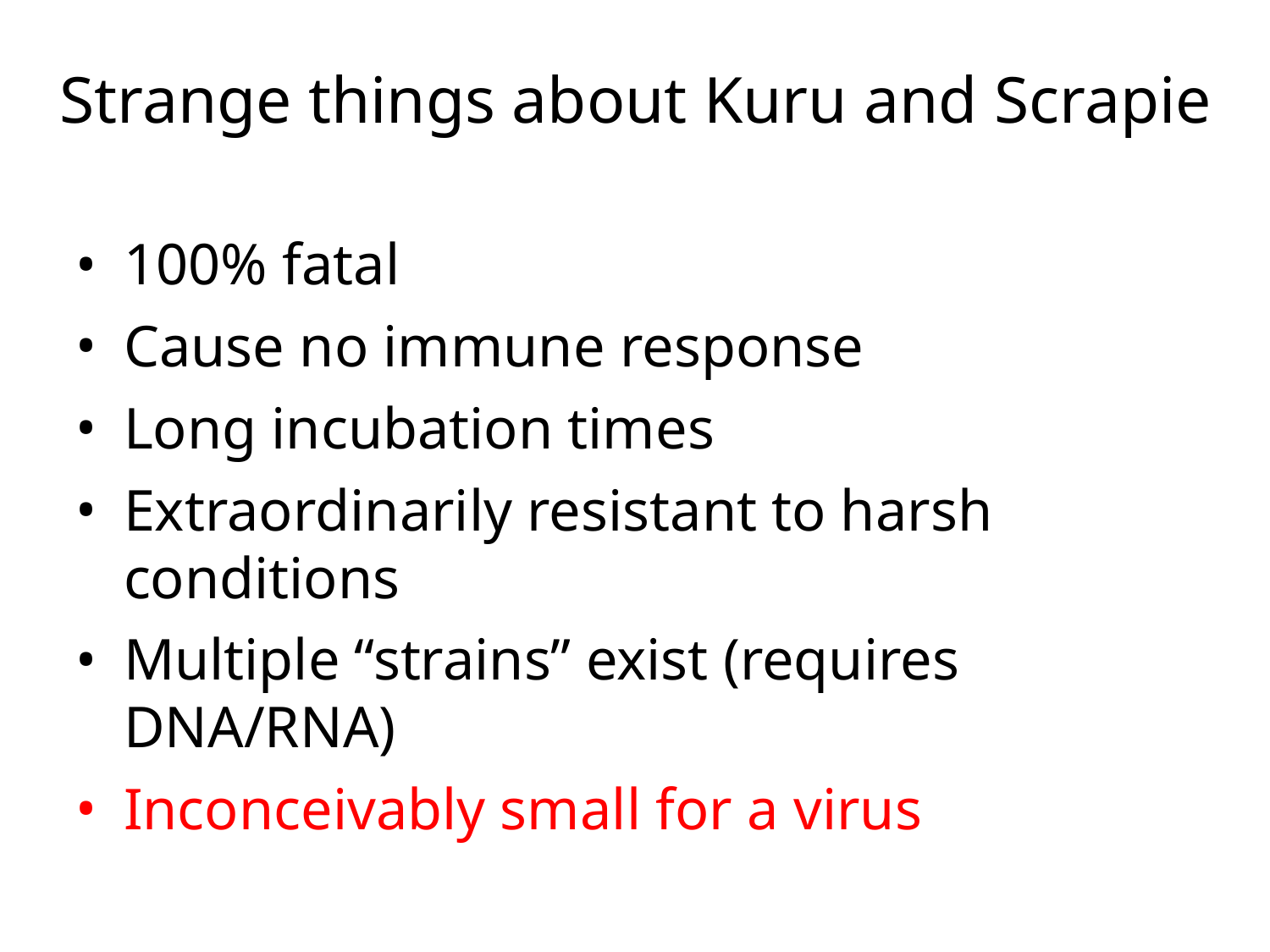

# Strange things about Kuru and Scrapie
100% fatal
Cause no immune response
Long incubation times
Extraordinarily resistant to harsh conditions
Multiple “strains” exist (requires DNA/RNA)
Inconceivably small for a virus

## Slide 11
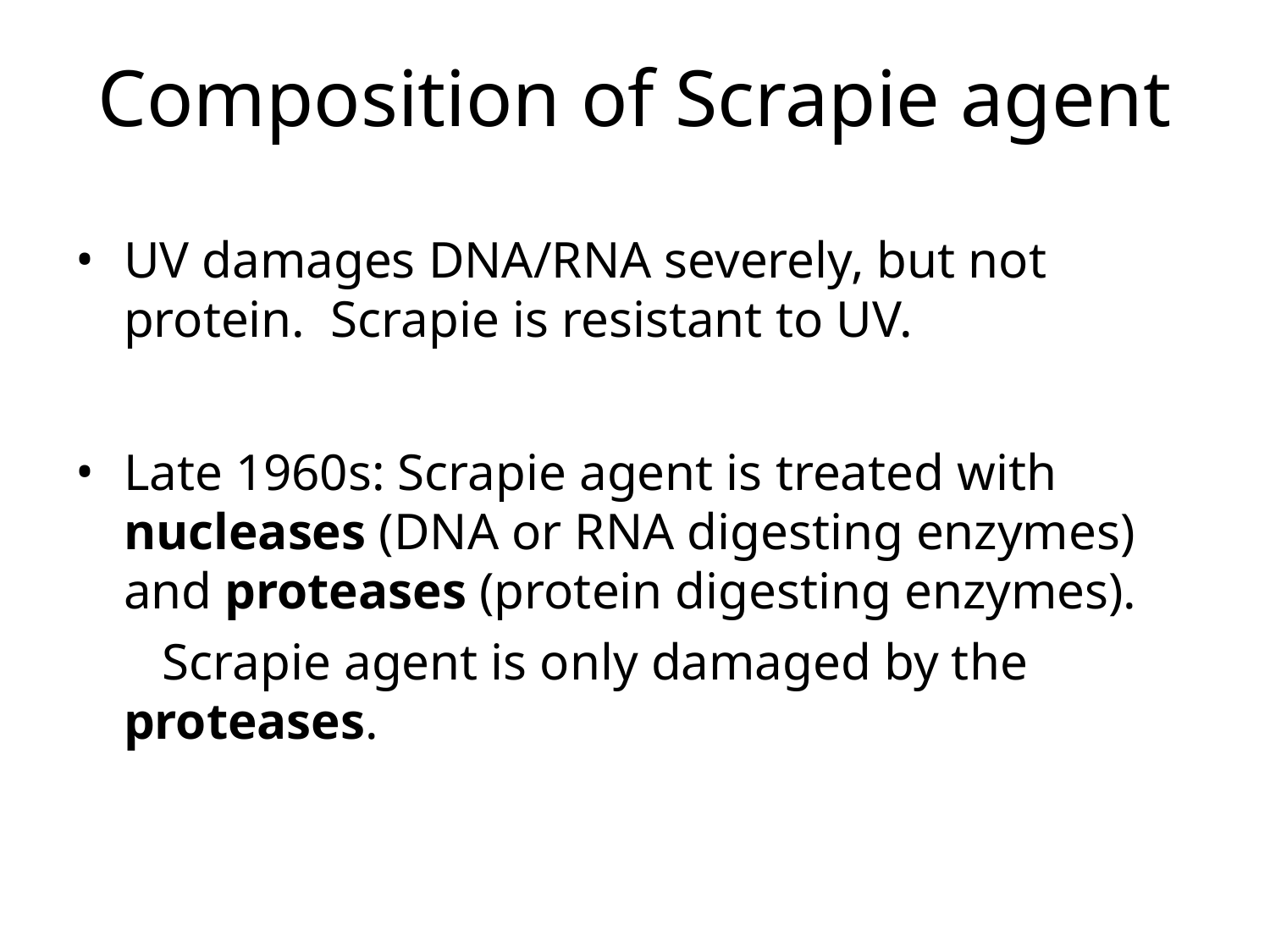

# Composition of Scrapie agent
UV damages DNA/RNA severely, but not protein. Scrapie is resistant to UV.
Late 1960s: Scrapie agent is treated with nucleases (DNA or RNA digesting enzymes) and proteases (protein digesting enzymes).
 Scrapie agent is only damaged by the proteases.

## Slide 12
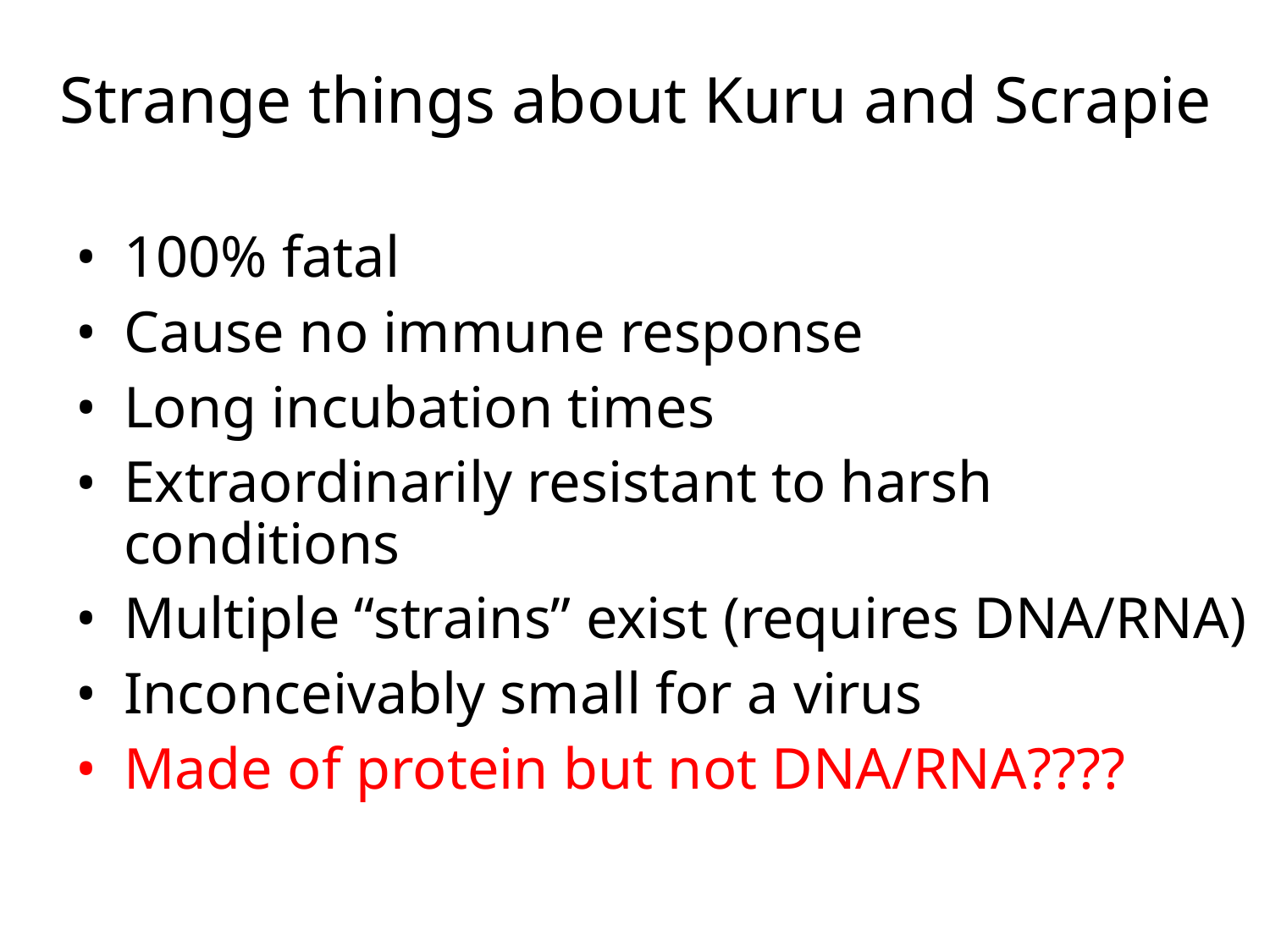

# Strange things about Kuru and Scrapie
100% fatal
Cause no immune response
Long incubation times
Extraordinarily resistant to harsh conditions
Multiple “strains” exist (requires DNA/RNA)
Inconceivably small for a virus
Made of protein but not DNA/RNA????

## Slide 13
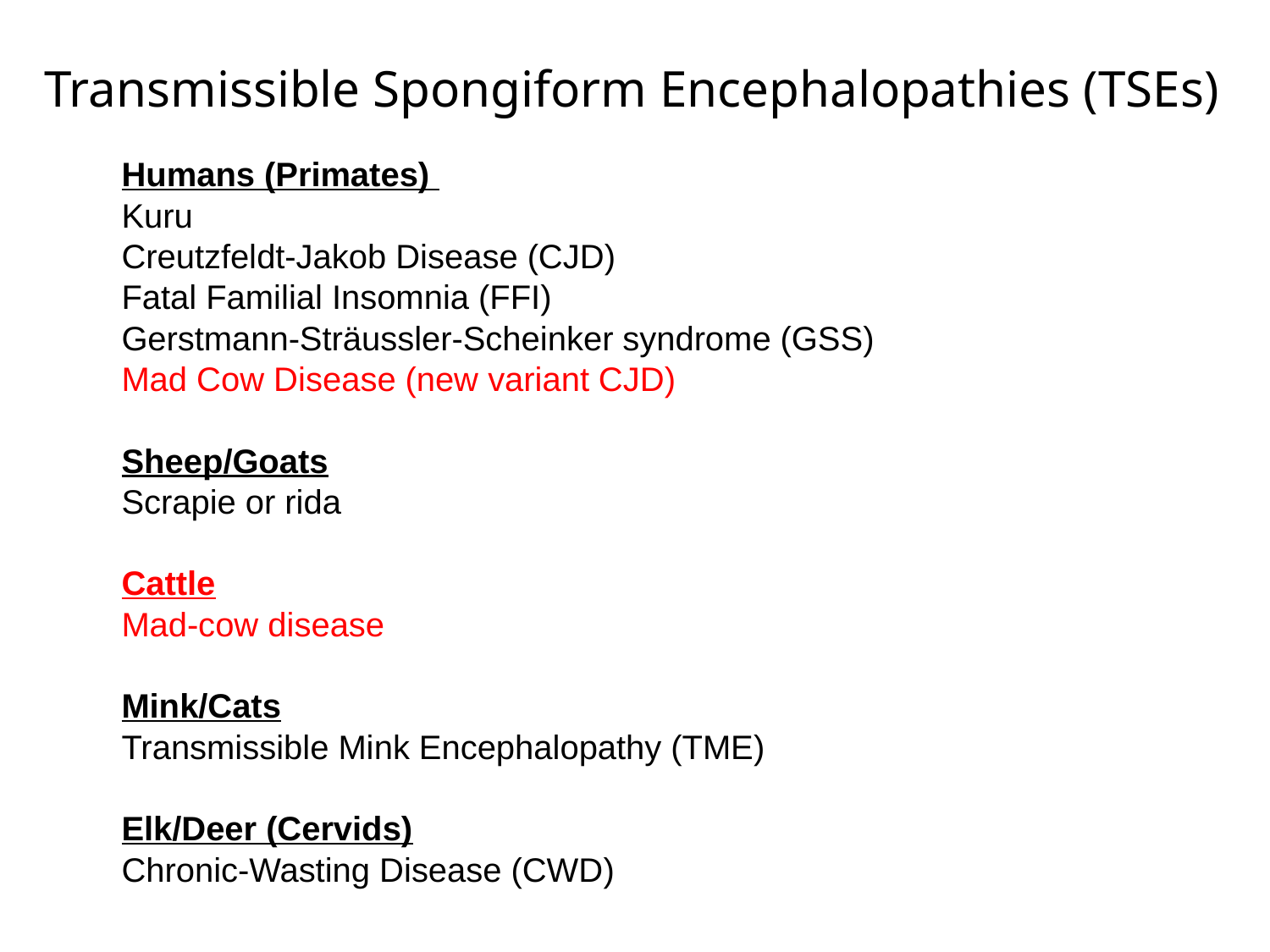

# Transmissible Spongiform Encephalopathies (TSEs)
Humans (Primates)
Kuru
Creutzfeldt-Jakob Disease (CJD)
Fatal Familial Insomnia (FFI)
Gerstmann-Sträussler-Scheinker syndrome (GSS)
Mad Cow Disease (new variant CJD)
Sheep/Goats
Scrapie or rida
Cattle
Mad-cow disease
Mink/Cats
Transmissible Mink Encephalopathy (TME)
Elk/Deer (Cervids)
Chronic-Wasting Disease (CWD)

## Slide 14
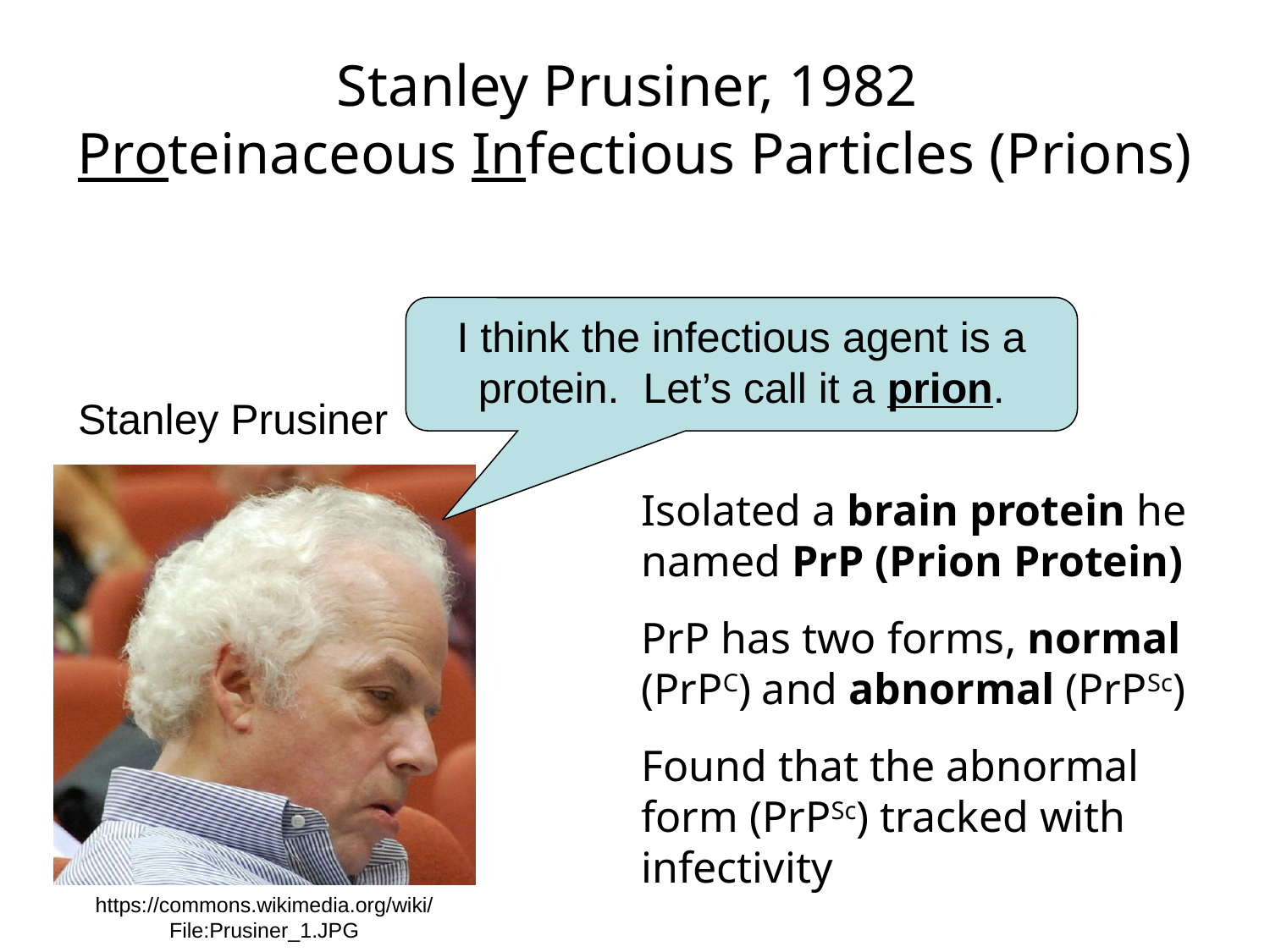

# Stanley Prusiner, 1982 Proteinaceous Infectious Particles (Prions)
I think the infectious agent is a protein. Let’s call it a prion.
Stanley Prusiner
Isolated a brain protein he named PrP (Prion Protein)
PrP has two forms, normal (PrPC) and abnormal (PrPSc)
Found that the abnormal form (PrPSc) tracked with infectivity
https://commons.wikimedia.org/wiki/File:Prusiner_1.JPG

## Slide 15
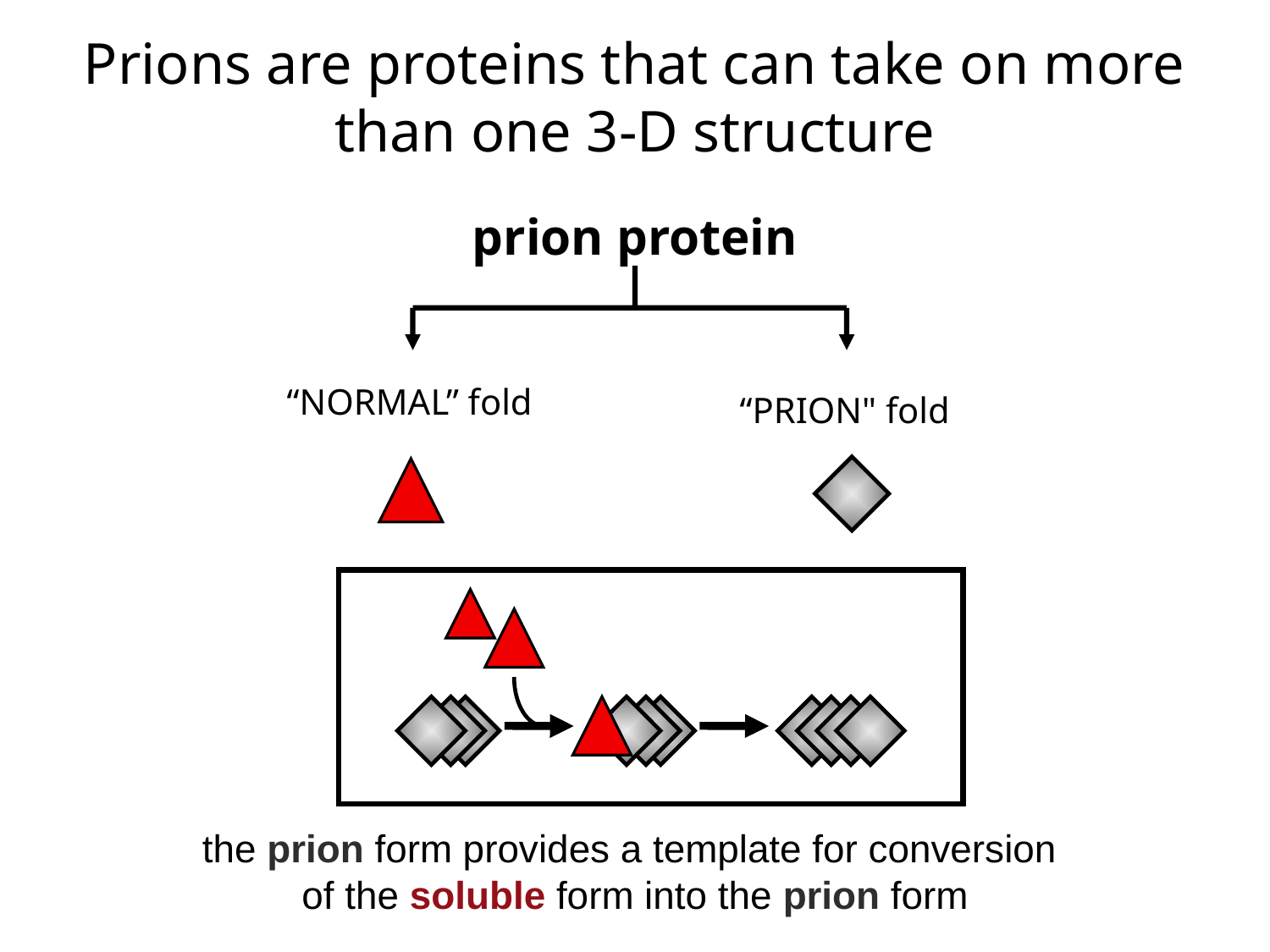

# Prions are proteins that can take on more than one 3-D structure
prion protein
“NORMAL” fold
“PRION" fold
the prion form provides a template for conversion
of the soluble form into the prion form

## Slide 16
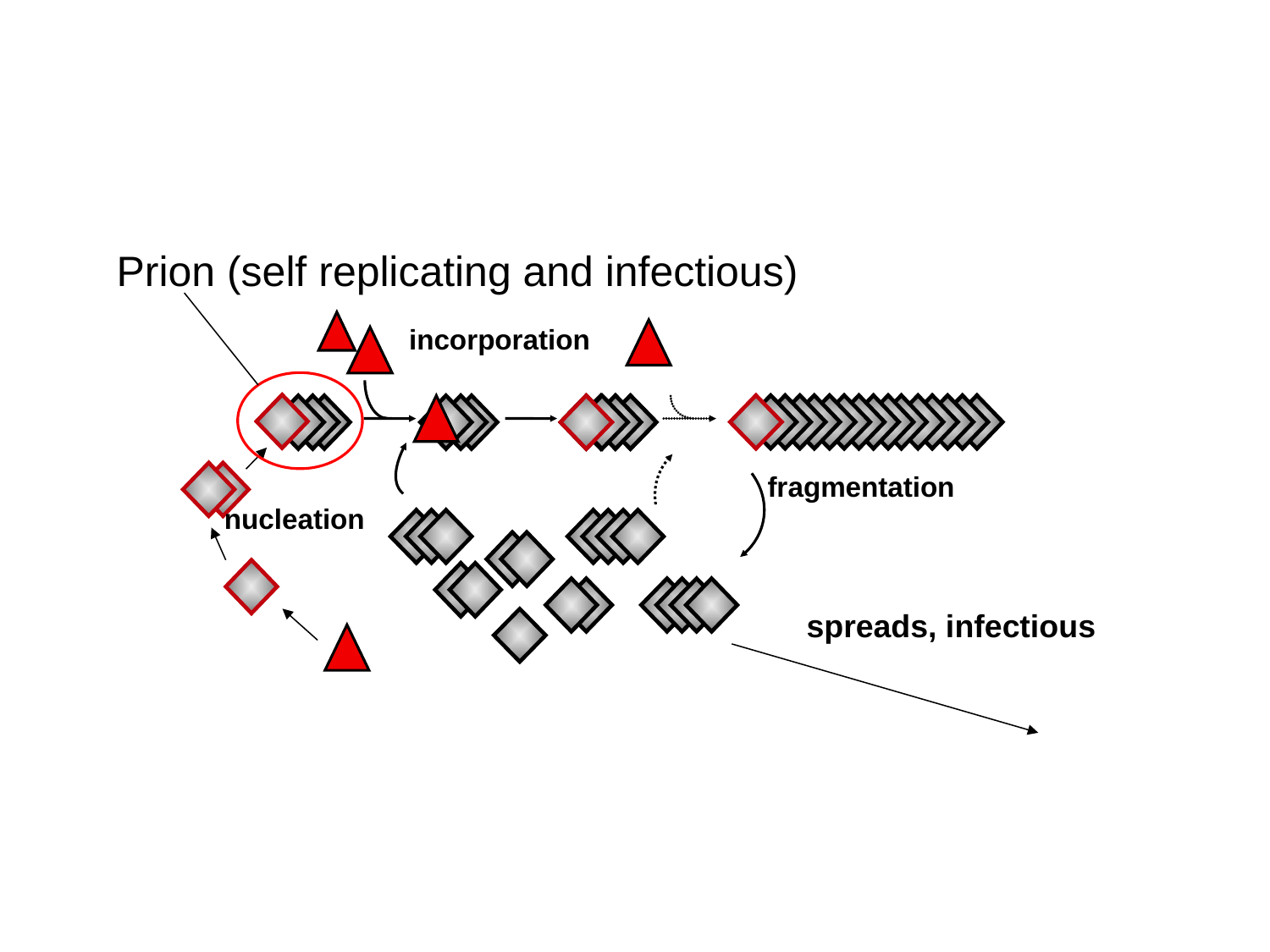

#
Prion (self replicating and infectious)
incorporation
fragmentation
nucleation
spreads, infectious

## Slide 17
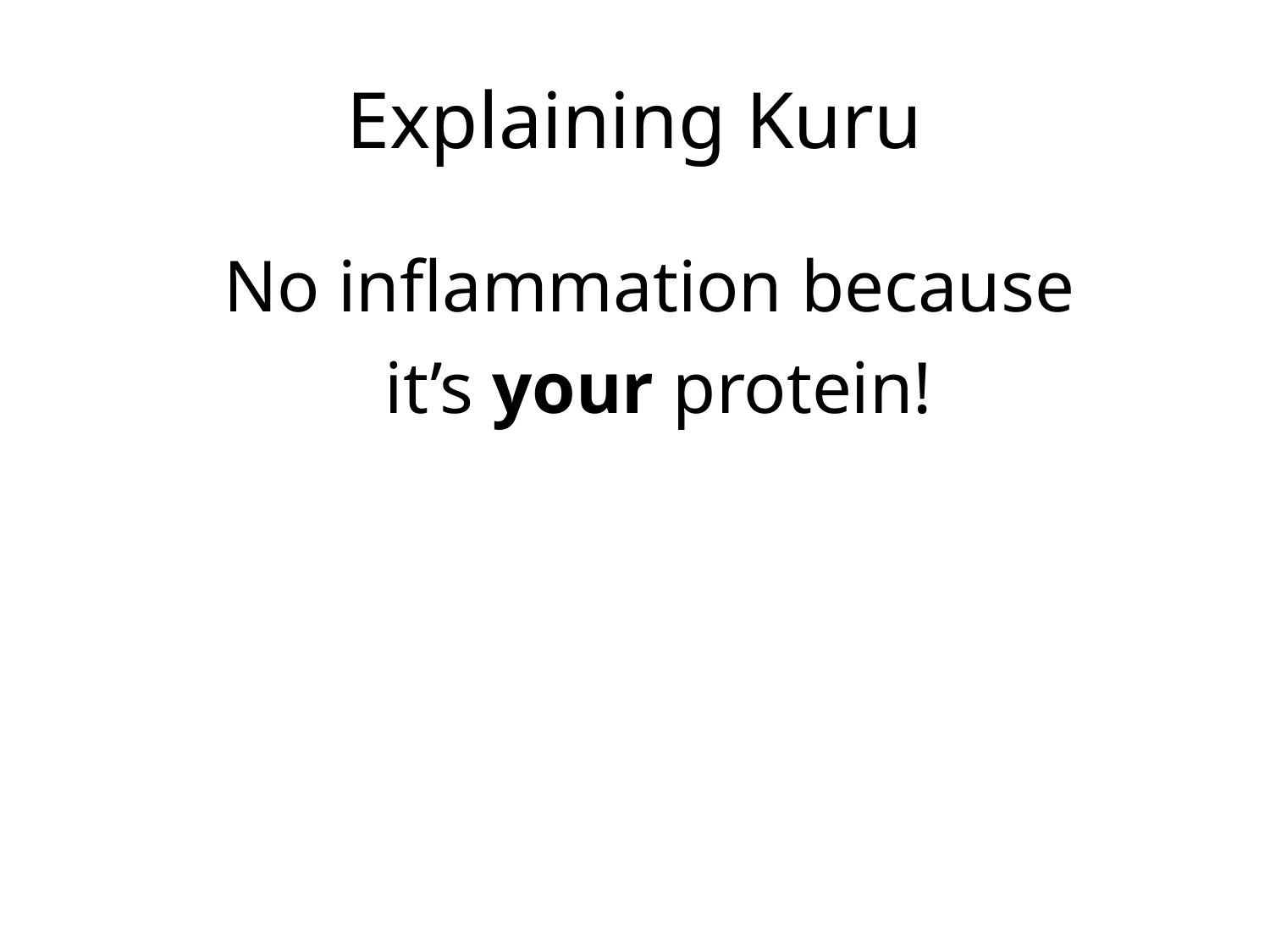

# Explaining Kuru
No inflammation because
it’s your protein!

## Slide 18
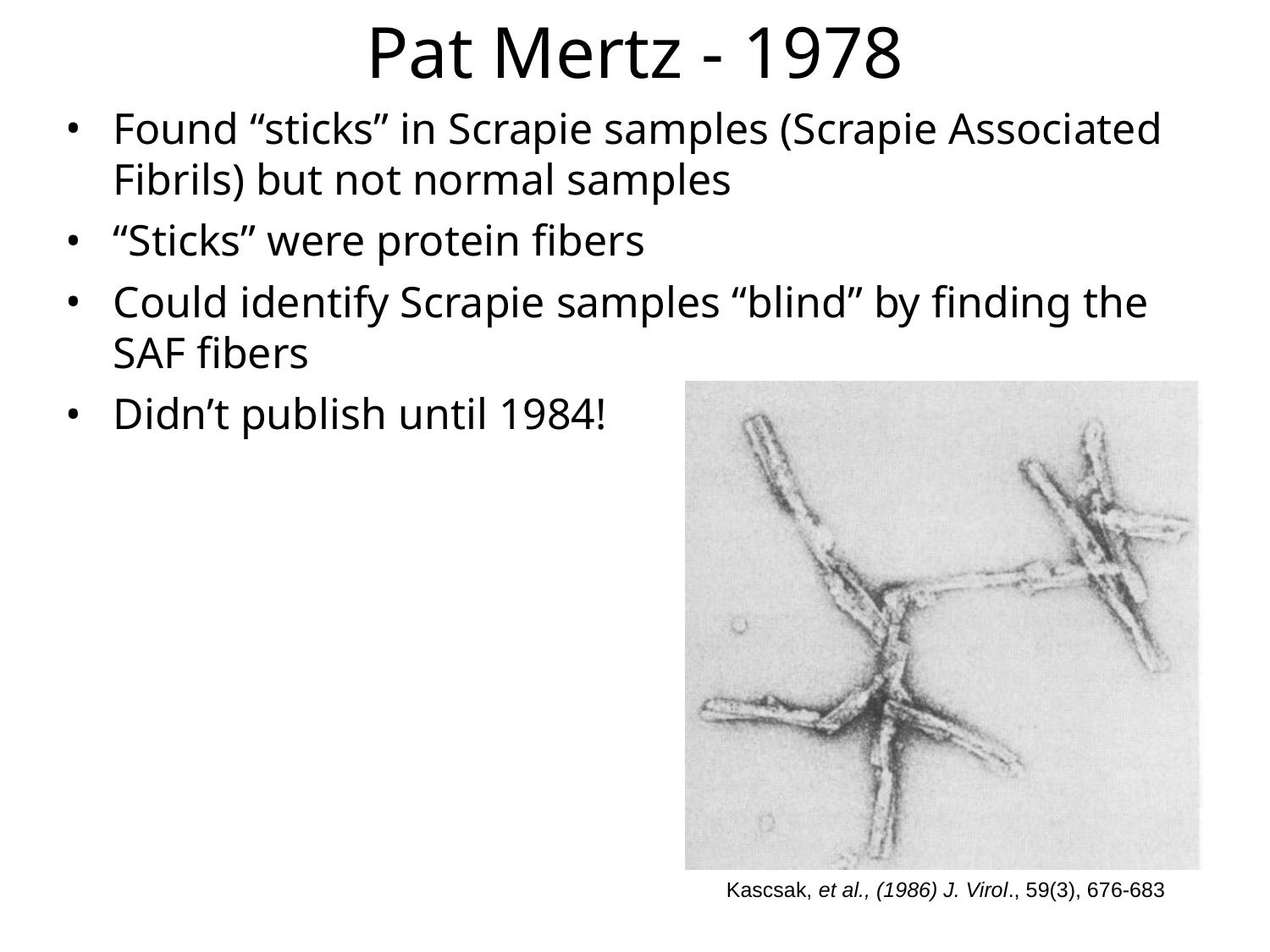

# Pat Mertz - 1978
Found “sticks” in Scrapie samples (Scrapie Associated Fibrils) but not normal samples
“Sticks” were protein fibers
Could identify Scrapie samples “blind” by finding the SAF fibers
Didn’t publish until 1984!
Kascsak, et al., (1986) J. Virol., 59(3), 676-683

## Slide 19
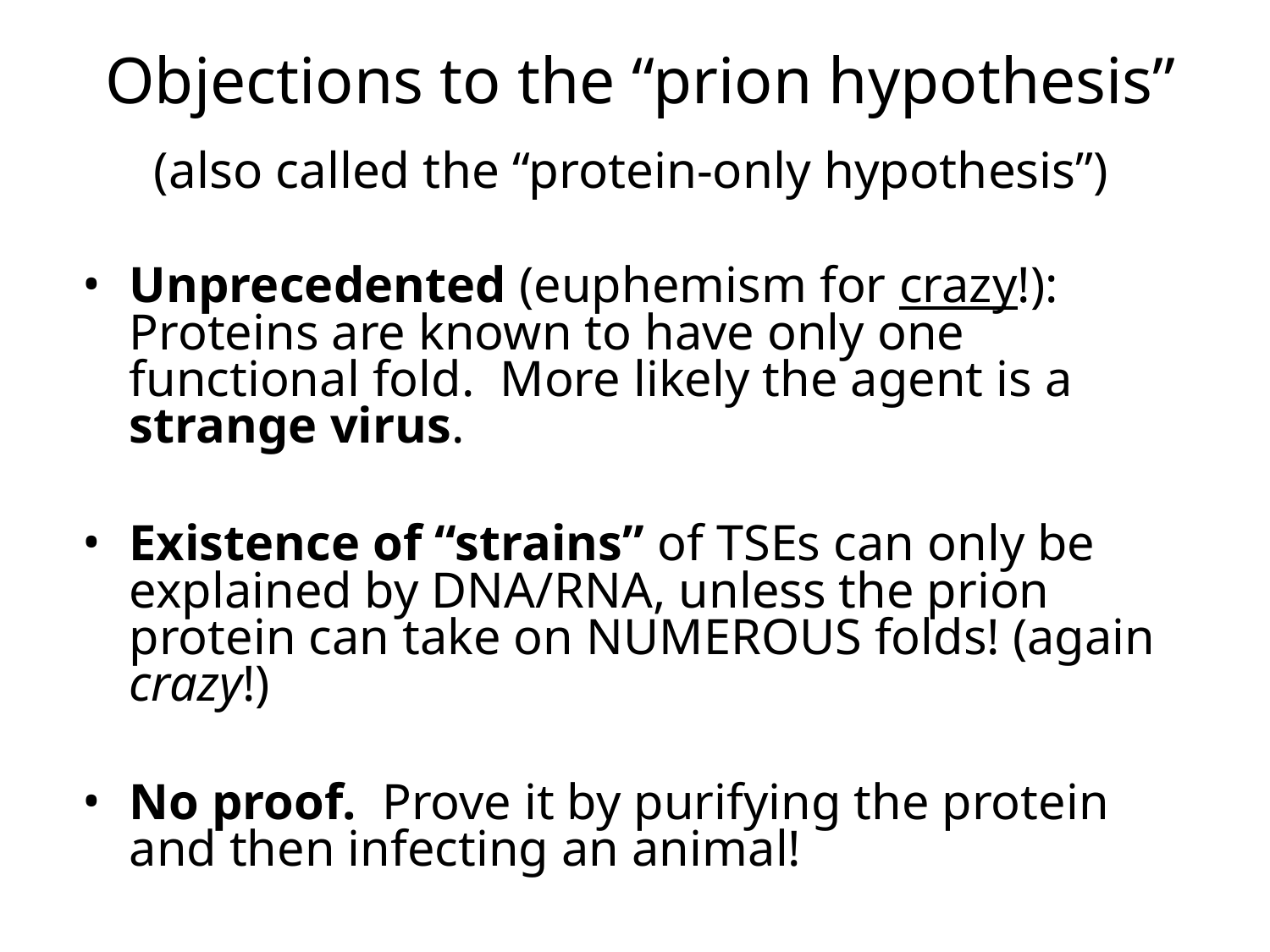

# Objections to the “prion hypothesis” (also called the “protein-only hypothesis”)
Unprecedented (euphemism for crazy!): Proteins are known to have only one functional fold. More likely the agent is a strange virus.
Existence of “strains” of TSEs can only be explained by DNA/RNA, unless the prion protein can take on NUMEROUS folds! (again crazy!)
No proof. Prove it by purifying the protein and then infecting an animal!

## Slide 20
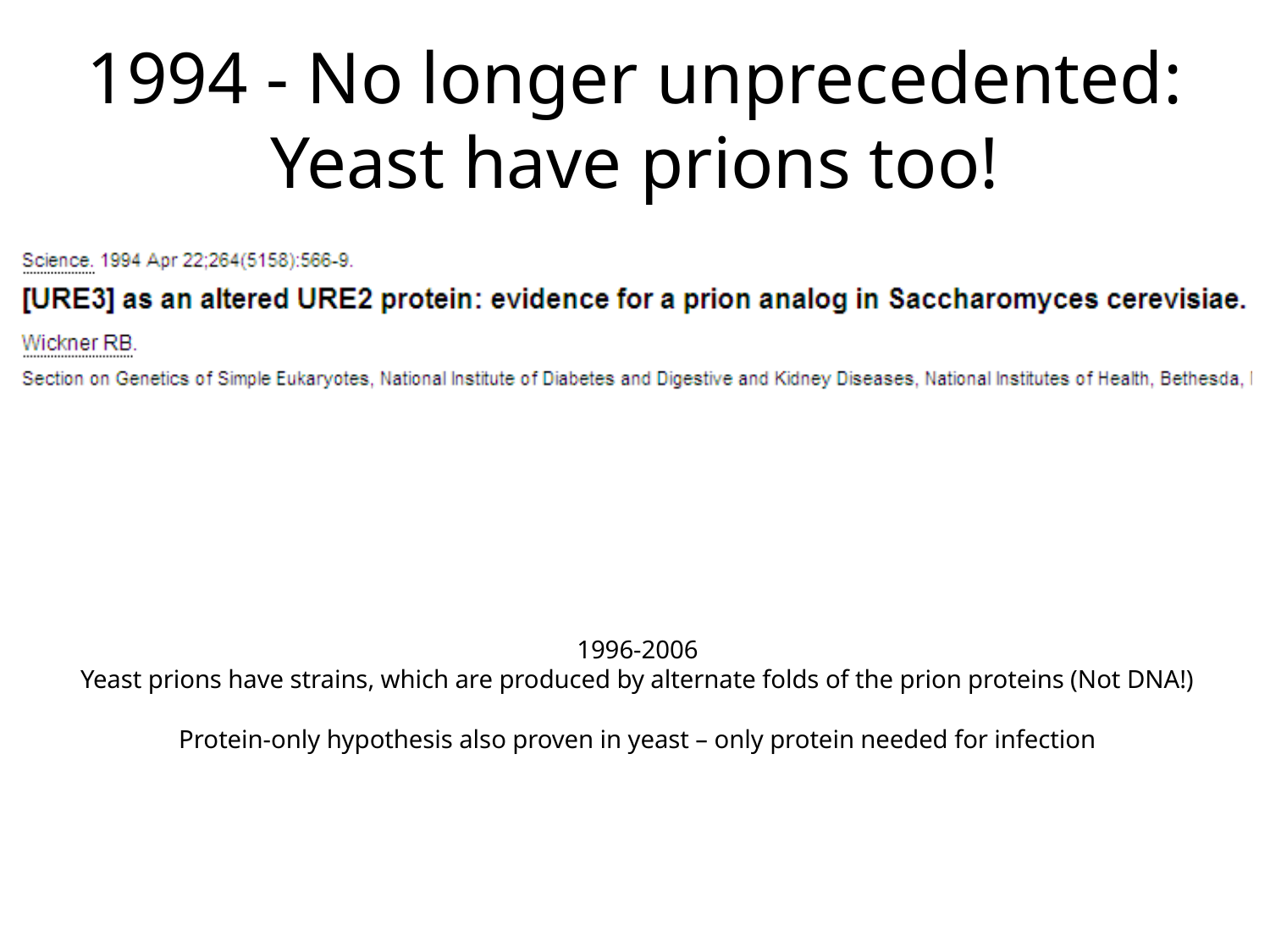

# 1994 - No longer unprecedented: Yeast have prions too!
1996-2006
Yeast prions have strains, which are produced by alternate folds of the prion proteins (Not DNA!)
Protein-only hypothesis also proven in yeast – only protein needed for infection

## Slide 21
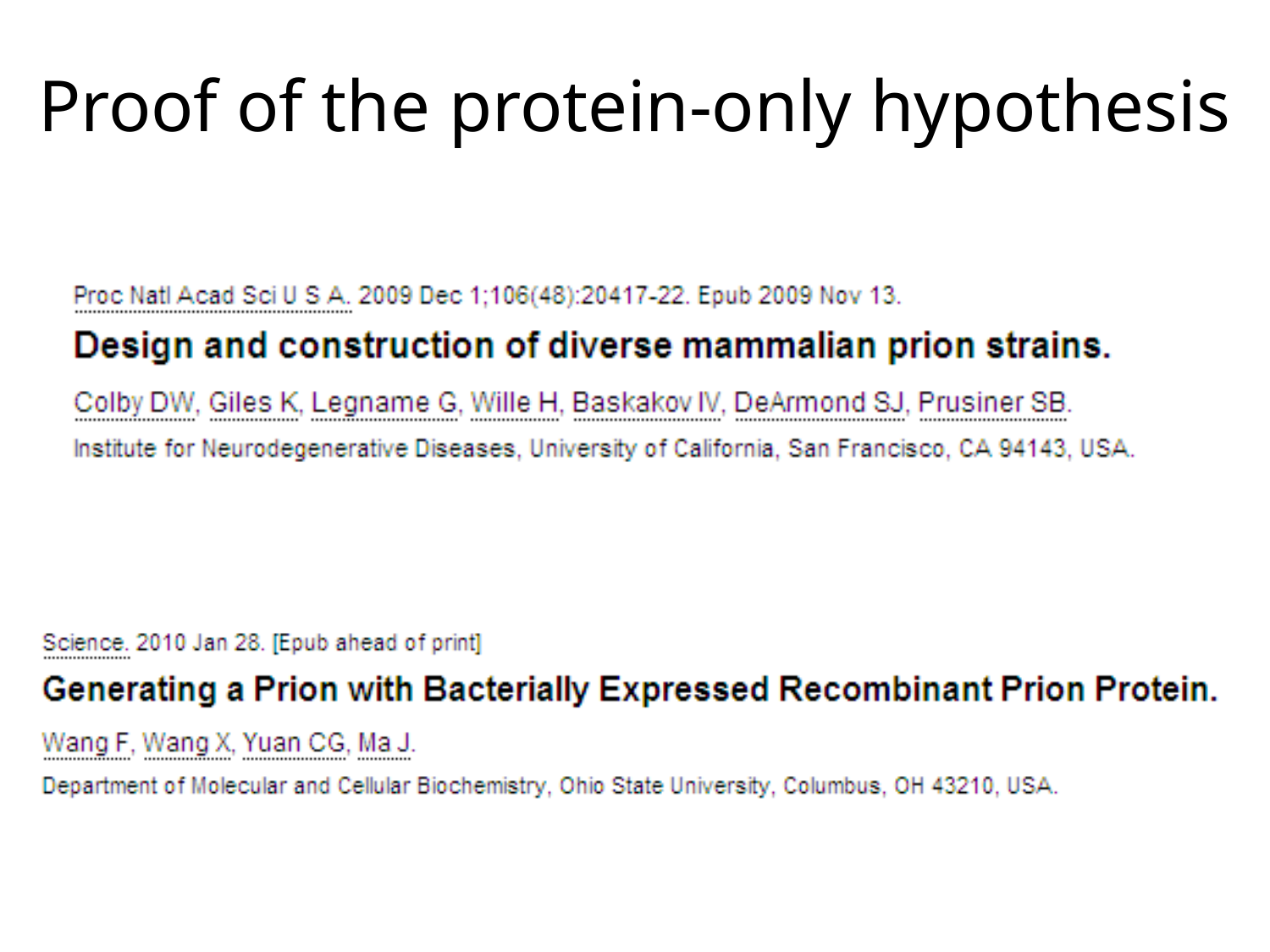

# Proof of the protein-only hypothesis

## Slide 22
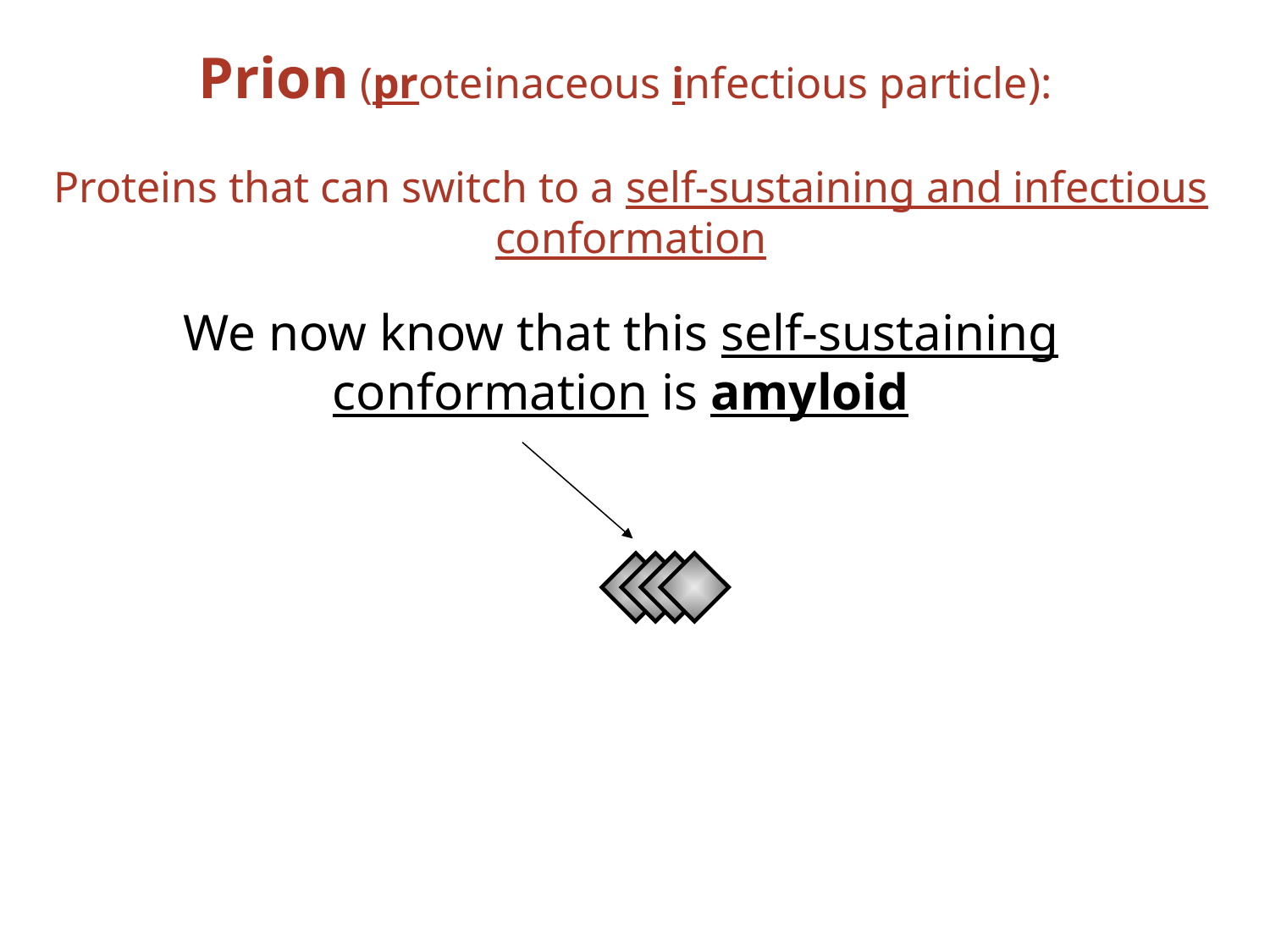

Prion (proteinaceous infectious particle):
Proteins that can switch to a self-sustaining and infectious conformation
We now know that this self-sustaining conformation is amyloid

## Slide 23
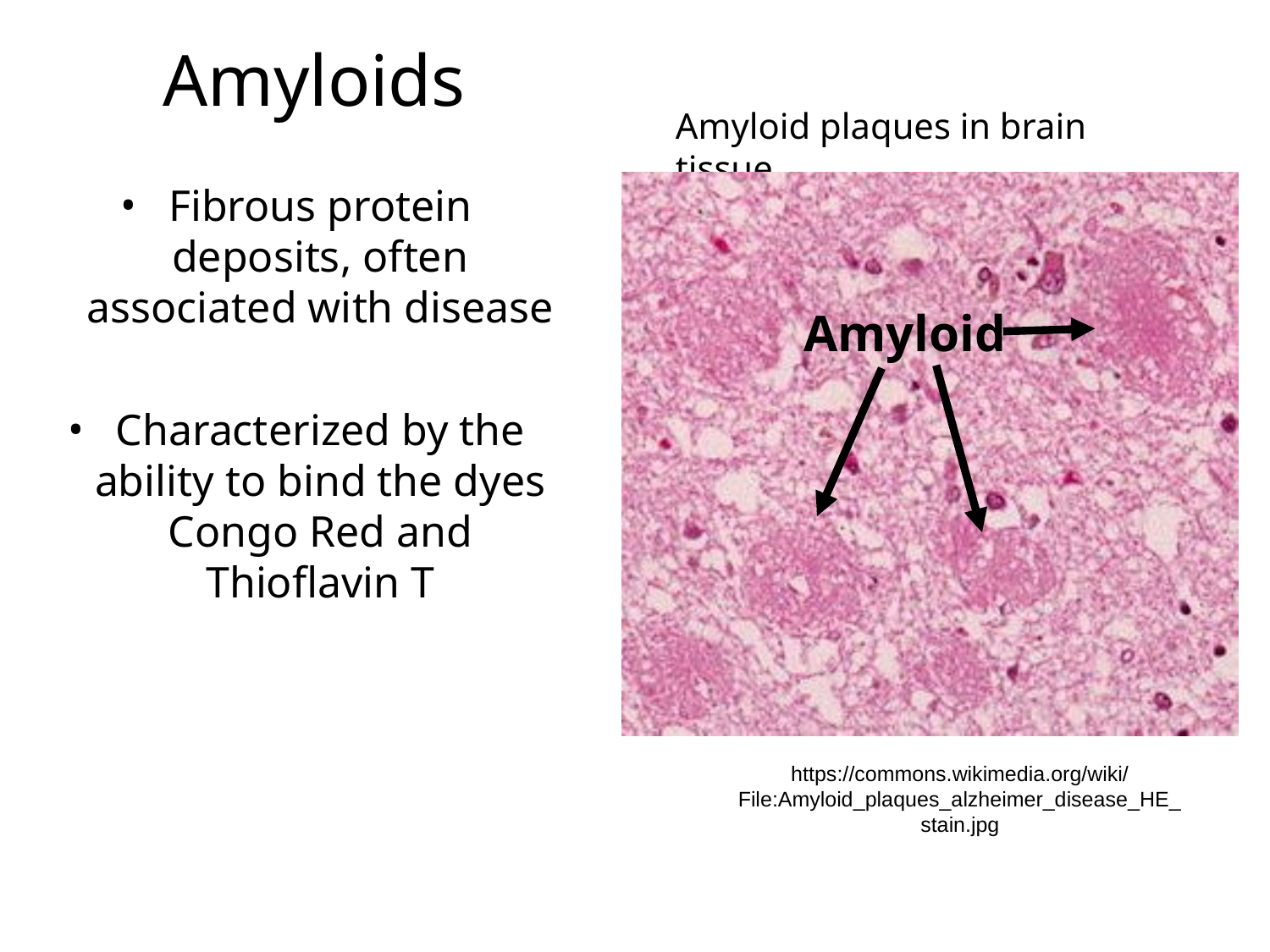

# Amyloids
Amyloid plaques in brain tissue
Fibrous protein deposits, often associated with disease
Characterized by the ability to bind the dyes Congo Red and Thioflavin T
Amyloid
https://commons.wikimedia.org/wiki/File:Amyloid_plaques_alzheimer_disease_HE_stain.jpg

## Slide 24
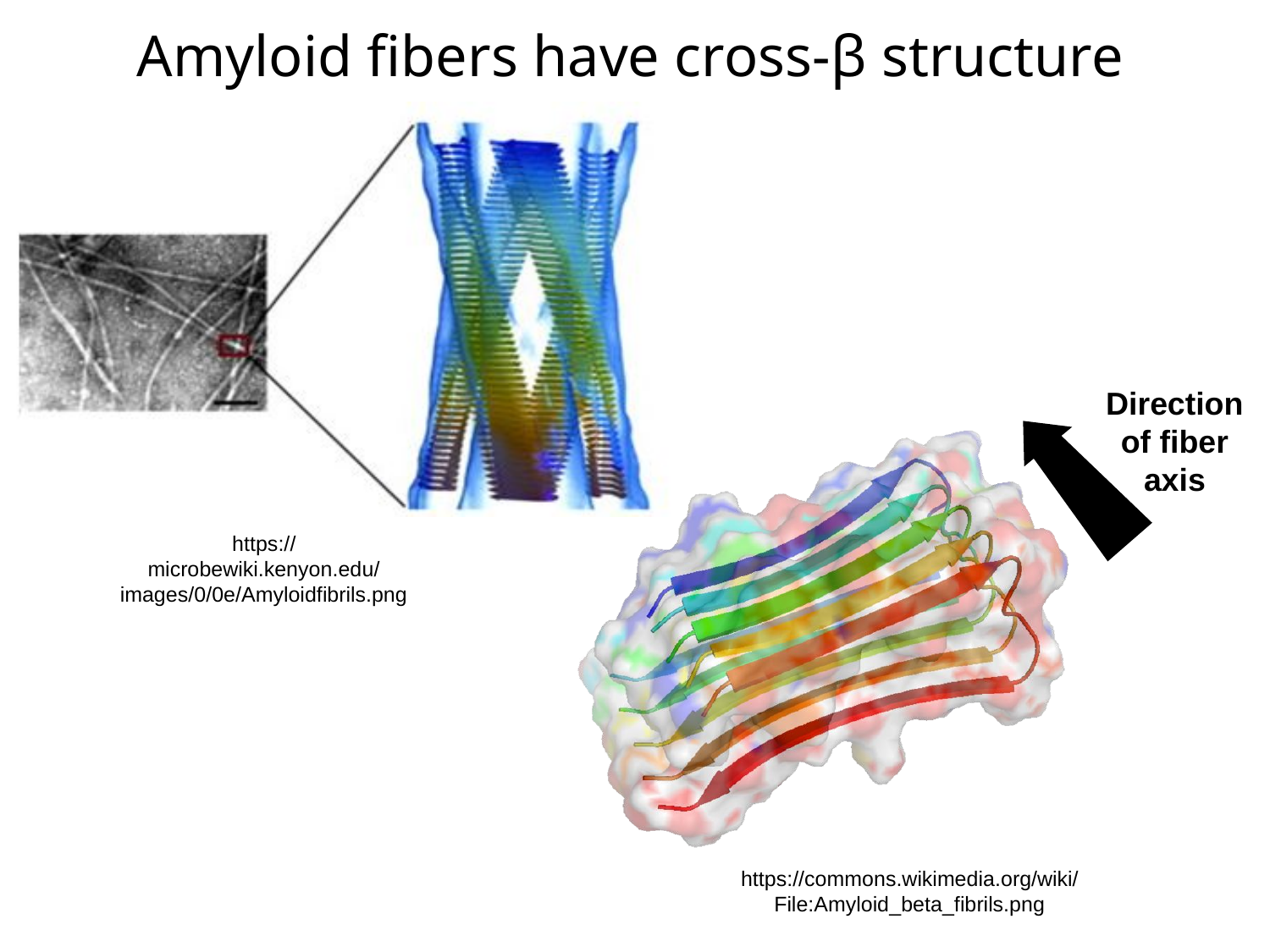

# Amyloid fibers have cross-β structure
Direction of fiber axis
https://microbewiki.kenyon.edu/images/0/0e/Amyloidfibrils.png
https://commons.wikimedia.org/wiki/File:Amyloid_beta_fibrils.png

## Slide 25
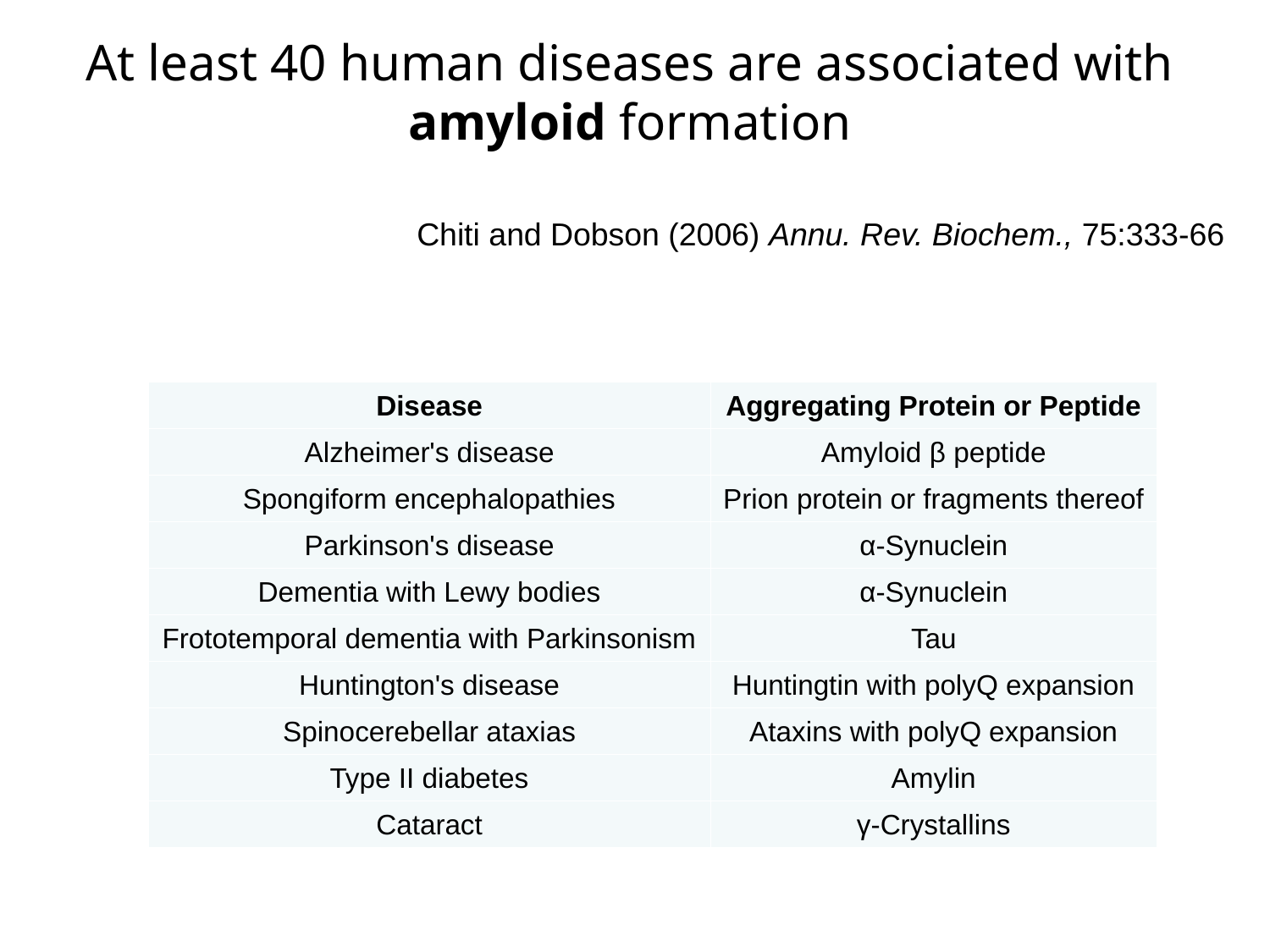

# At least 40 human diseases are associated with amyloid formation
Chiti and Dobson (2006) Annu. Rev. Biochem., 75:333-66
| Disease | Aggregating Protein or Peptide |
| --- | --- |
| Alzheimer's disease | Amyloid β peptide |
| Spongiform encephalopathies | Prion protein or fragments thereof |
| Parkinson's disease | α-Synuclein |
| Dementia with Lewy bodies | α-Synuclein |
| Frototemporal dementia with Parkinsonism | Tau |
| Huntington's disease | Huntingtin with polyQ expansion |
| Spinocerebellar ataxias | Ataxins with polyQ expansion |
| Type II diabetes | Amylin |
| Cataract | γ-Crystallins |

## Slide 26
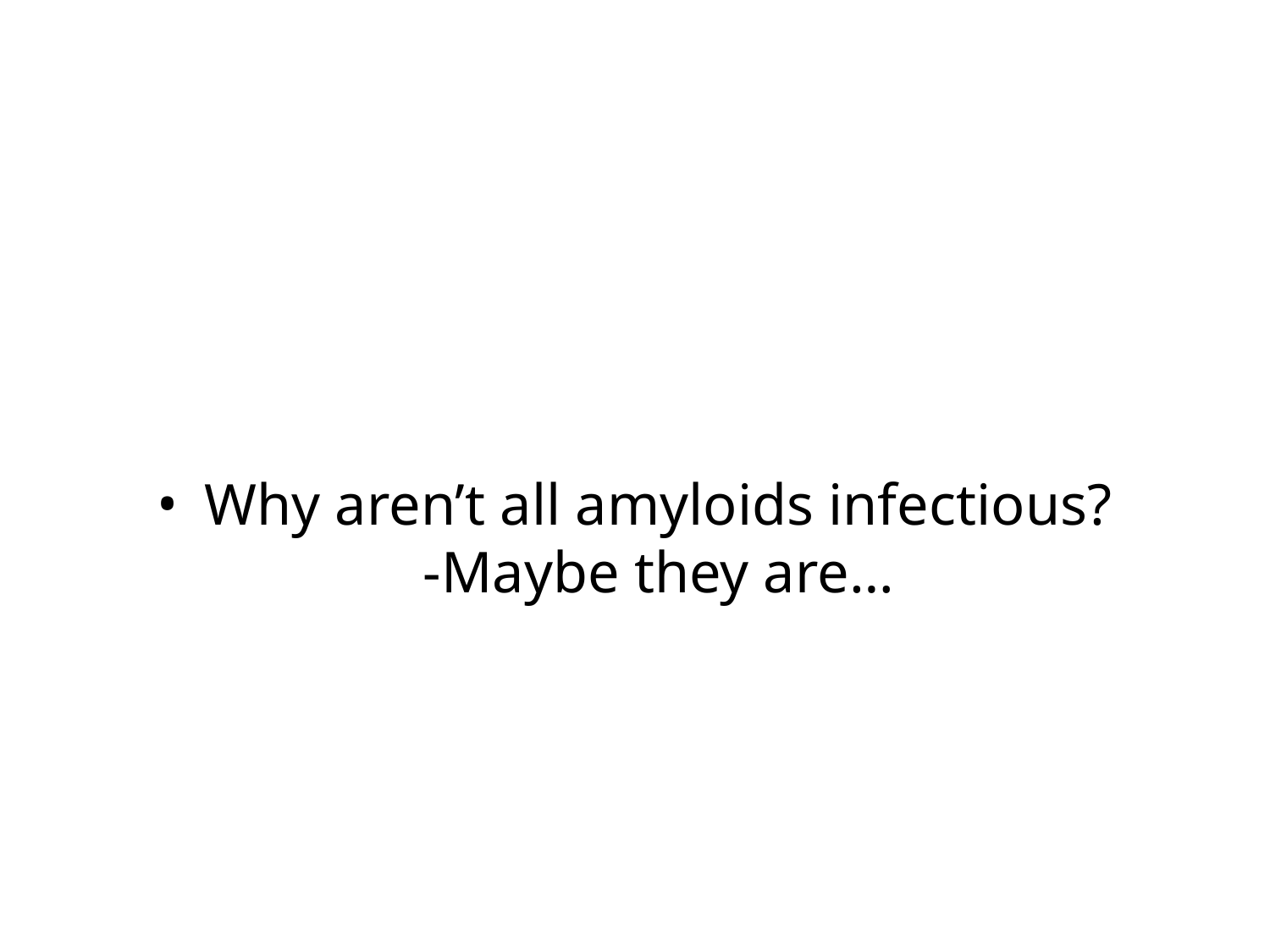

#
Why aren’t all amyloids infectious?
-Maybe they are…

## Slide 27
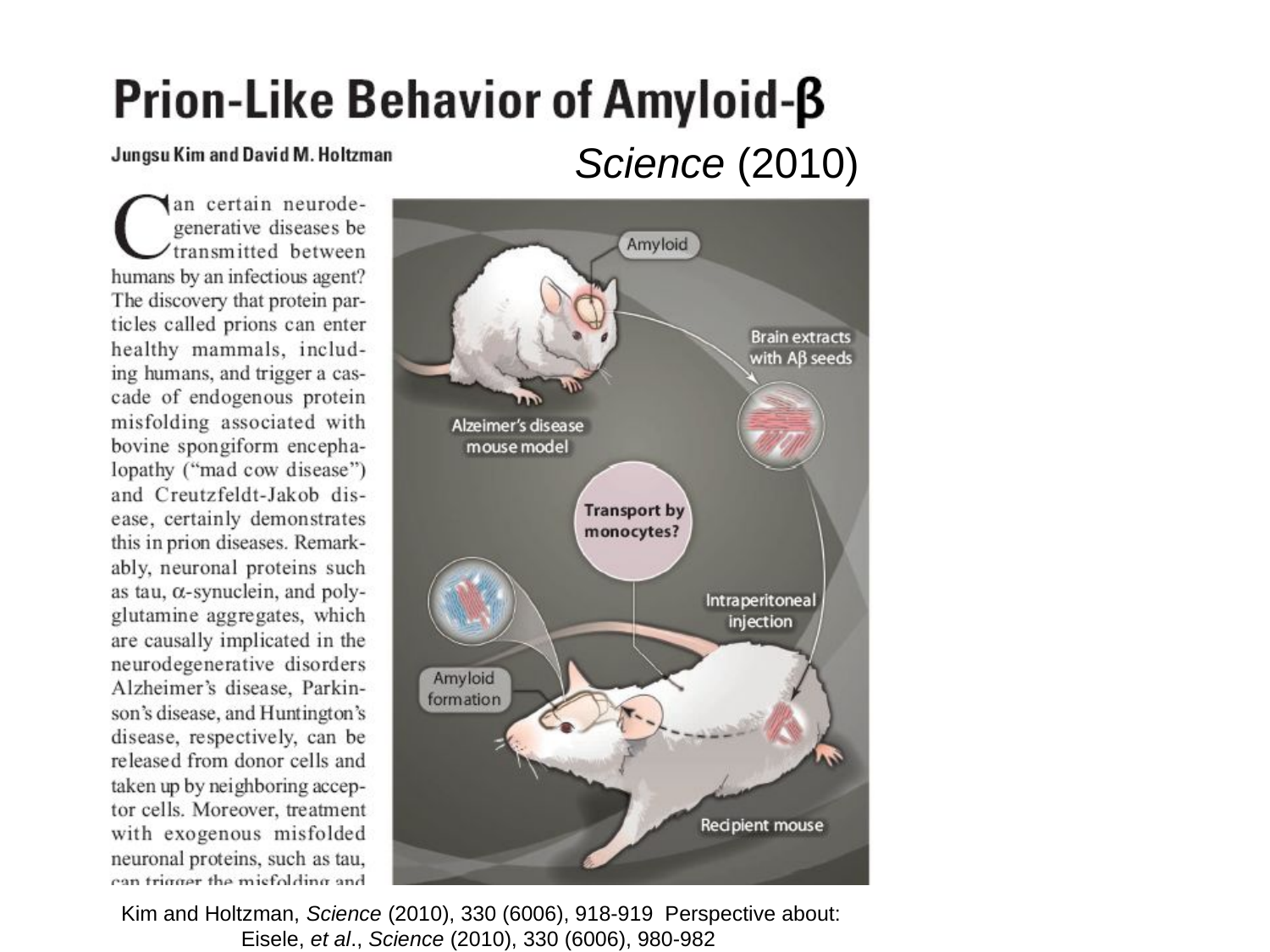

Science (2010)
Kim and Holtzman, Science (2010), 330 (6006), 918-919 Perspective about:
Eisele, et al., Science (2010), 330 (6006), 980-982

## Slide 28
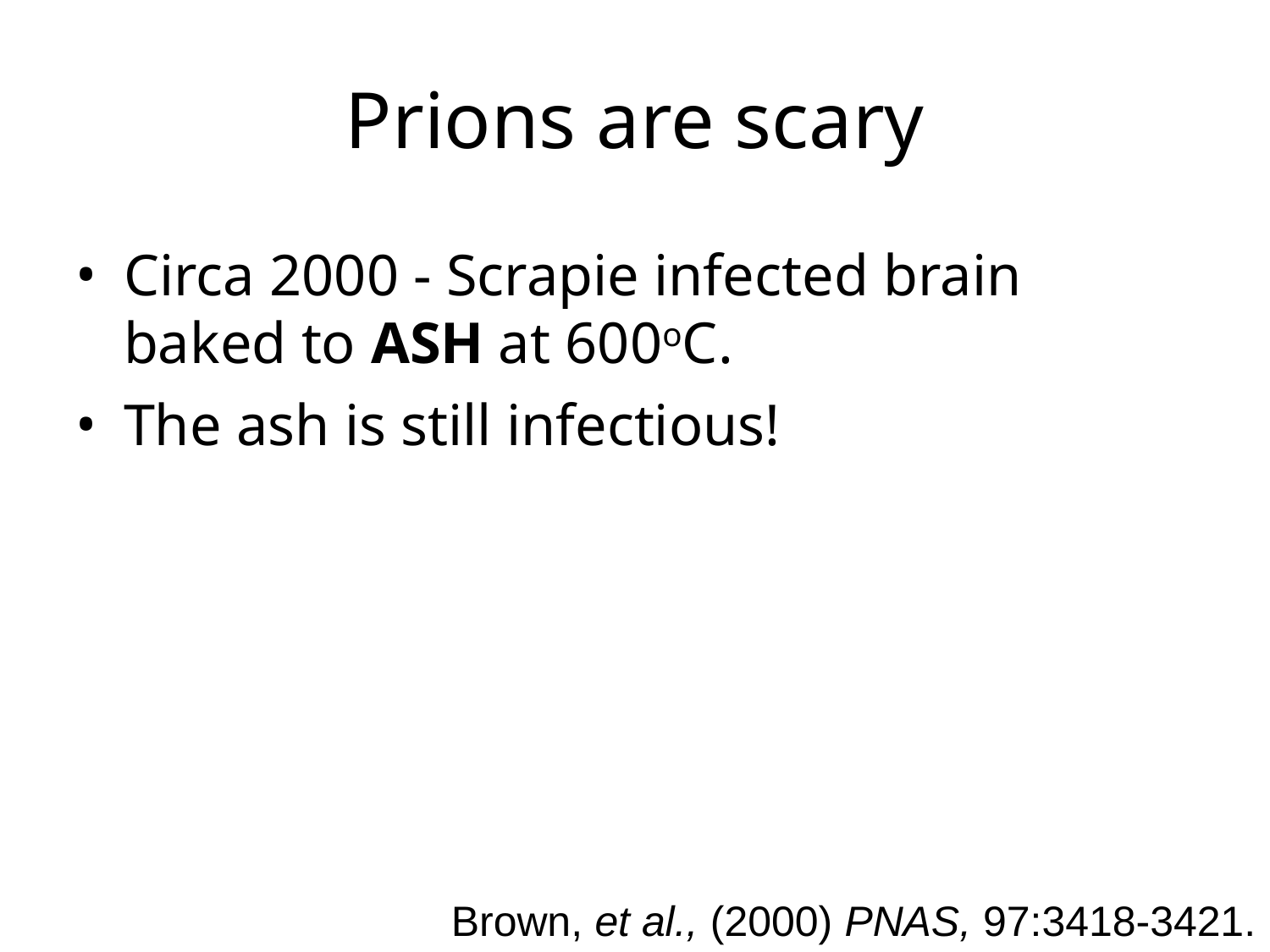

# Prions are scary
Circa 2000 - Scrapie infected brain baked to ASH at 600oC.
The ash is still infectious!
Brown, et al., (2000) PNAS, 97:3418-3421.

## Slide 29
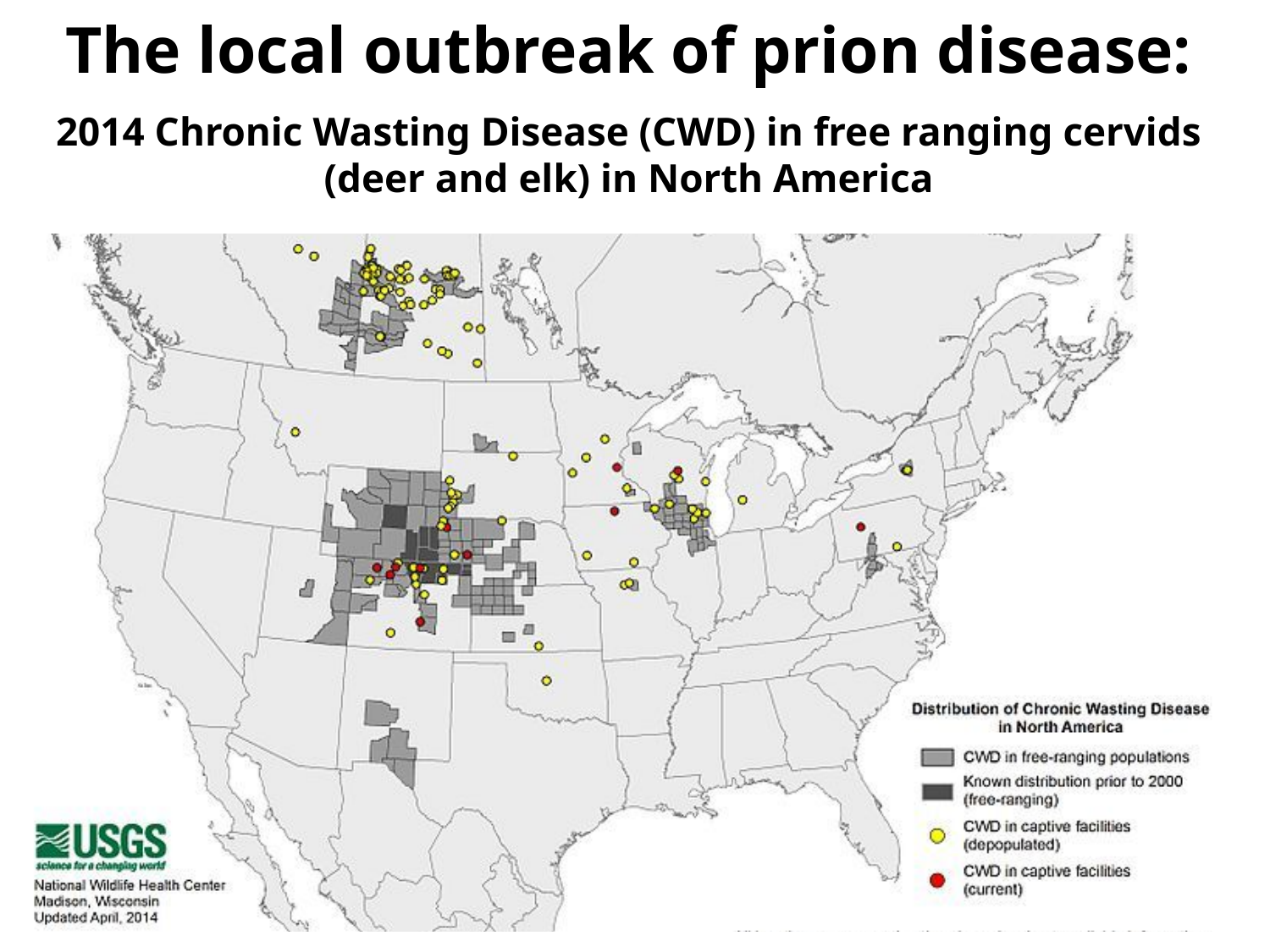

# The local outbreak of prion disease: 2014 Chronic Wasting Disease (CWD) in free ranging cervids (deer and elk) in North America

## Slide 30
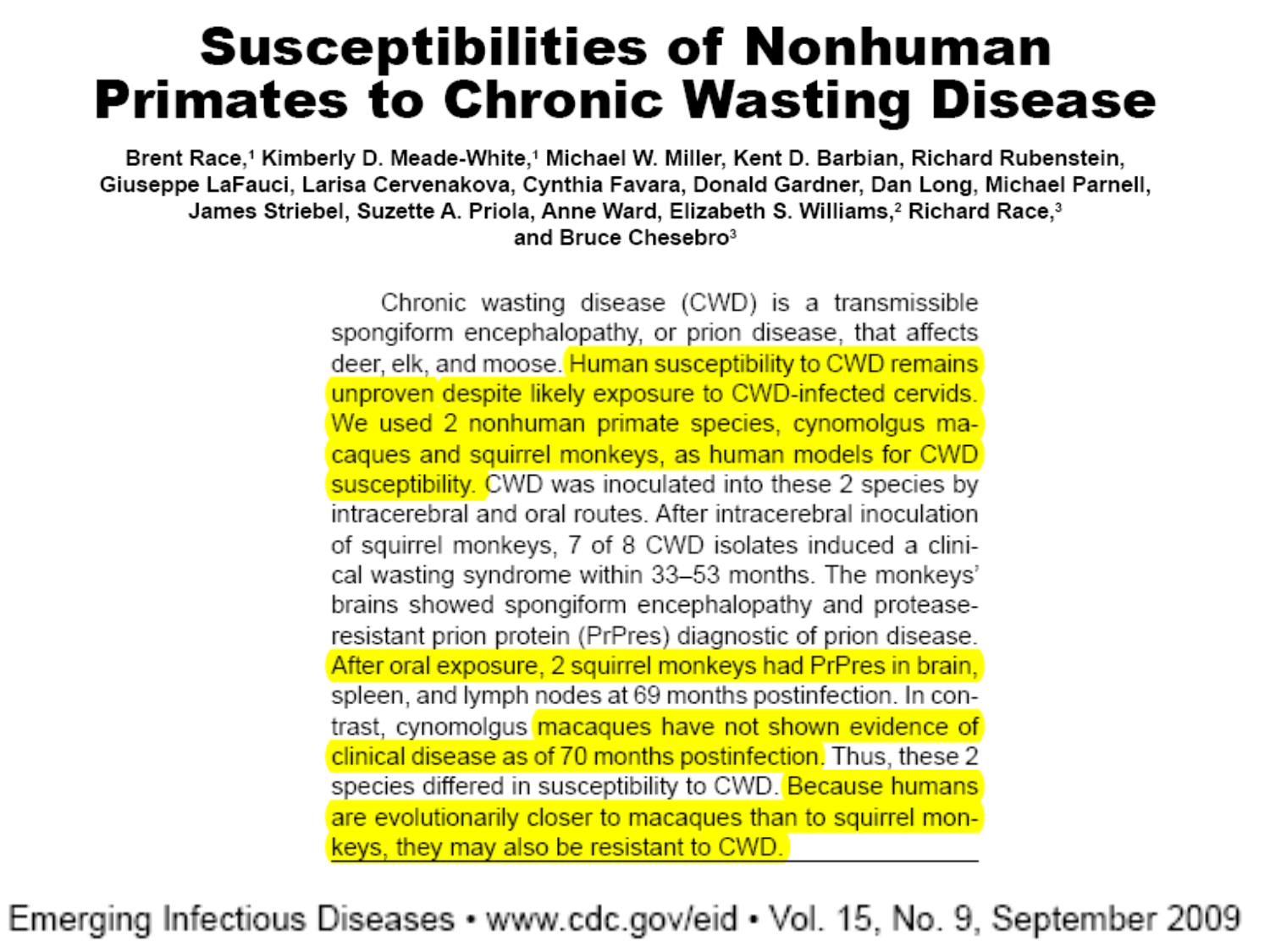

Supplement: S2 PowerPoint — (PPT) [file pbio.1002425.s002.ppt]
